# Peptide Multifunctionalization via Modular Construction of *Trans*‐AB_2_C Porphyrin on Resin

**DOI:** 10.1002/advs.202409771

**Published:** 2025-02-19

**Authors:** Yue Wu, Yuen‐Ting Wong, Yik‐Hoi Yeung, Pak‐Lun Lam, Ho‐Fai Chau, Wing‐Sze Tam, Qian Zhang, William C. S. Tai, Ka‐Leung Wong

**Affiliations:** ^1^ Department of Applied Biology and Chemical Technology The Hong Kong Polytechnic University 11 Yuk Choi Rd, Hung Hom Hong Kong SAR China; ^2^ Department of Chemistry Hong Kong Baptist University 224 Waterloo Rd, Kowloon Tong Hong Kong SAR China

**Keywords:** multifunctional peptide, peptide ligation, porphyrin, porphyrin‐peptide conjugates, solid‐phase peptide synthesis

## Abstract

Peptide multifunctionalization is a crucial technique to develop peptide‐based agents for various purposes. Porphyrin‐peptide conjugates are a class of popular multifunctional peptides renowned for their multifunctional and multimodal properties. However, the tedious synthetic works for porphyrin building blocks are involved in most previous studies. In this work, a modular solid‐phase synthetic approach is reported to construct *trans*‐AB_2_C porphyrin on peptide chains without presynthesized porphyrin building blocks. The products from this approach, which inherit both functionalities from the porphyrins and the modules employed for constructing porphyrins, show potential in biomedical and biomaterial applications. Furthermore, by extending this synthetic approach, the first example of “resin‐to‐resin” reaction is reported to link two peptides together along the construction of porphyrin motifs to give porphyrin‐peptide conjugates with two different peptide chains.

## Graphical abstract

A modular solid‐phase synthetic protocol is reported to directly construct *trans‐*AB_2_C porphyrin moieties on resin‐bound peptides from only simple modules. The synthesized porphyrin‐peptide conjugates gain functionalities from both porphyrin and module. Various modules are compatible with this approach to make the desired products easily diversifiable.

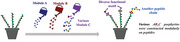

## 1 Introduction

Peptide derivatives are becoming more popular as they constituted over 10 % of novel drugs approved by The United States Food and Drug Administration (US FDA) in the past 2 years.^[^ ^1^ ^]^ In drug discovery, the targets based on protein‐protein interactions (PPIs) were considered “undruggable” (difficult to develop small molecule drugs for them) due to their large and shallow interface. Peptide derivatives are proven as promising therapeutic and diagnostic agents for PPIs‐based targets.^[^ ^2^, ^3^ ^]^ Additionally, adequate biocompatibility of peptides makes them ideal platforms for developing drug delivery systems^[^ ^4^ ^]^ and biocompatible materials.^[^ ^5^ ^]^ Peptide functionalization endows the peptides with additional and unique functionalities, such as luminescence,^[^ ^6^, ^7^ ^]^ radioactivity,^[^ ^8^, ^9^ ^]^ magnetic relaxivity,^[^ ^10^, ^11^ ^]^ additional target specificity,^[^ ^12^ ^]^ enhanced therapeutic efficacy,^[^ ^13^, ^14^ ^]^ alternative self‐assembly tendency,^[^ ^15^ ^]^ improved stability, and cell‐penetration,^[^ ^16^, ^17^ ^]^ etc. In some cases, peptides with multiple above‐mentioned functionalities are needed. For example, many bimodal imaging probes combining a radiotracer and a fluorescent dye have been designed to utilize the advantage of both radiology (deep tissue penetration) and optical imaging (high‐resolution).^[^ ^18^, ^19^, ^20^, ^21^, ^22^ ^]^ Other combinations, like multiple radiotracers,^[^ ^23^, ^24^ ^]^ or fluorescent dyes, and an additional targeting motif,^[^ ^25^ ^]^ were also reported. Moreover, to design protease sensors, multiple fluorescent dyes were deployed onto a peptide that could be cleaved at a specific site by the corresponding protease to dramatically change its fluorescence by switching the energy transfer between two dyes.^[^ ^27^, ^28^, ^29^ ^]^ Furthermore, to achieve dual‐targeting imaging, some studies have fused two targeting peptides together with a radiotracer or fluorescent dyes.^[^ ^30^, ^31^, ^32^ ^]^ The advancement of techniques for peptide multifunctionalization, which enable the production of the above‐mentioned compounds, has largely contributed to the flourishing of peptide derivatives.^[^ ^33^, ^34^ ^]^

**Figure 1:**
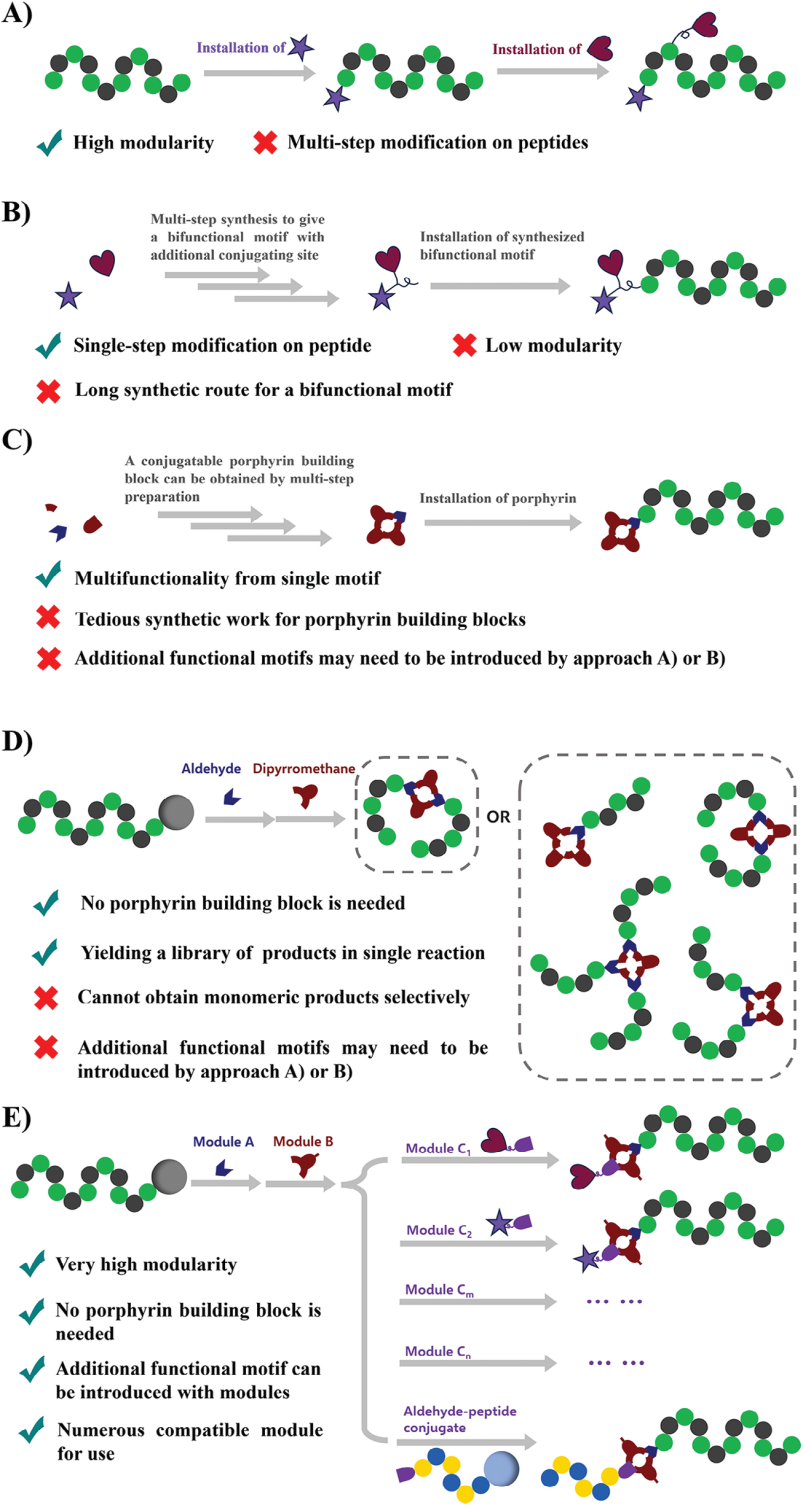
The approaches for peptide multifunctionalization. A) Multi‐time functionalization of a peptide by solid‐phase conjugation or site‐specific solution phase conjugation; B) Functionalization of a peptide with a multi‐component functional motif that was presynthesize in advance via a multi‐step synthetic route; C) Functionalization of a peptide with a multifunctional motif like porphyrin; D) Our previous work: solid‐phase construction of porphyrin‐peptide conjugates; E) This work: modular construction of porphyrin on peptide chain. The modules can be easily diversified to introduce additional functional motifs on demand.

Peptides are usually synthesized by solid‐phase peptide synthesis (SPPS) that incorporates partially protected amino acids (e.g. the amino acid with Fmoc‐protected α‐amine and unprotected α‐COOH) into a peptide chain modularly and sequentially.^[^ ^35^, ^36^ ^]^ In each cycle, excess amino acid building blocks are used to ensure complete incorporation, and the Fmoc protecting group is then removed to release an amine group for incorporating the next amino acid. Peptides are frequently functionalized on their N‐terminal (also known as capping) by incorporating SPPS‐compatible functional building blocks (e.g., acid‐resistant fluorescent dyes, chelators, and drug molecules with one unprotected ‐COOH) in the last step of SPPS. Using orthogonally protected amino acids, peptides can even be functionalized by multiple functional motifs during SPPS after selectively removing a side‐chain protecting group of amino acids (e.g., lysine).^[^ ^37^ ^]^ Besides, site‐specific conjugation reactions allow unprotected peptides to be regioselectively modified at a late stage, examples include copper‐catalyzed azide‐alkyne cycloaddition (CuAAC),^[^ ^38^ ^]^ strain‐promoted azide‐alkyne cycloaddition (SPAAC),^[^ ^39^ ^]^ thiol‐maleimide ligation,^[^ ^40^ ^]^ etc. Although synthetic approaches based on the combination of above‐mentioned techniques have been widely used to prepare multifunctional peptides, most of the previous works conjugate a single functional motif in each step, which means multi‐step functionalization was inevitable (**Figure** **1**A). Alternatively, many previous studies presynthesized multifunctional motifs by covalently linking multiple functional compartments before conjugating to peptides (Figure 1B). Although this approach reduced the step applied on peptides, the synthetic routes are usually much longer, and the low modularity of this approach makes it incompatible with modern strategies in the discovery of drugs and materials like late‐stage functionalization (LSF),^[^ ^41^ ^]^ and diversity‐oriented synthesis (DOS).^[^ ^42^ ^]^ Therefore, developing more efficient and less tedious modular protocols that offer multiple functionalities to a peptide still attracts much research interest.

Porphyrin‐peptide conjugates,^[^ ^43^, ^44^ ^]^ regarded as a family of multifunctional peptides due to their multifunctionality inherited from porphyrin motifs, have been used for targeting photodynamic therapy (PDT),^[^ ^45^, ^46^, ^47^, ^48^ ^]^ photothermal therapy (PTT),^[^ ^49^ ^]^ fluorescent cellular imaging,^[^ ^50^, ^51^, ^52^ ^]^ catalysis (e.g., artificial enzyme),^[^ ^53^, ^54^ ^]^ metal sensor, and chelating,^[^ ^55^, ^56^, ^57^ ^]^ and self‐assembly.^[^ ^58^, ^59^, ^60^, ^61^, ^62^, ^63^ ^]^ For making porphyrin‐peptide conjugates, porphyrin building blocks with conjugating site(s) on the *meso* positions (1,5,10,15‐positions) of the porphyrin were usually prepared (Figure 1C).^[^ ^64^ ^]^ Although the synthetic protocols for porphyrin are long‐established, the synthesis of porphyrin building blocks with high degree of asymmetricity (e.g., A_3_B, *trans*‐AB_2_C) still suffers from low yields and tedious purification steps as statistical porphyrin synthesis leads to a mixture of porphyrins with different configurations. Moreover, some previous studies introduced functional motifs in addition to porphyrin to expand the application of desired products,^[^ ^65^, ^66^ ^]^ additional synthetic steps were therefore involved to make those multi‐component porphyrin‐peptide conjugates.

To circumvent the tedious synthetic procedures of “pyrrole chemistries” (e.g. the condensation between pyrroles and electrophiles to yield dipyrrins/BODIPYs, porphyrins, corroles), our recent studies successfully combined these reactions with solid‐phase syntheses to offer a facile platform to remove excess reactants/reagents and unattached by‐products from the resin‐bound products.^[^ ^67^, ^68^, ^69^, ^70^ ^]^ Although the porphyrin‐peptide conjugates have been successfully constructed during solid‐phase synthesis in our previous study,^[^ ^69^ ^]^ the reaction led to either *trans*‐bis‐substituted product (*trans*‐A_2_B_2_) or a mixture of mono‐ (AB_3_), bis‐ (*trans* and *trans*‐A_2_B_2_), tris‐ (A_3_B) substituted products (Figure 1D). As mono‐substituted porphyrin‐peptide conjugates (AB_3_) are the most attractive/common in applications, we therefore continued to seek a synthetic approach toward them in a more controllable and selective manner. Furthermore, we desire to introduce a functional motif during the construction of porphyrin to fulfill the demand for additional functionalities that cannot be provided by porphyrins themselves.

In this work, we developed a modular synthetic methodology to construct *trans‐*AB_2_C‐porphyrins on peptide chains by orderly introducing three modules (Figure 1E). Upon incorporating the first two modules (A and B), an aldehyde building block (module C) can be further introduced to complete the construction of porphyrin motifs. Notably, the abundant and diverse sources of aldehydes enable easy product diversification. By changing the modules, diverse porphyrin‐peptide conjugates can be prepared effectively. Moreover, additional functional groups can be introduced to give extra functionality (e.g., bioorthogonal chemistry, surface modification of nanomaterials, formation of polymers, etc.) without prolonging the synthetic route. Furthermore, another resin‐bound peptide could serve as module C upon functionalizing by an aldehyde; this unprecedented “resin‐to‐resin” reaction can fuse two peptides together with a porphyrin motif. The synthetic approach in this study is totally compatible with the concepts of late‐stage functionalization (LSF) and diversity‐oriented synthesis (DOS), and demonstrates a framework to effectively and efficiently produce a library of porphyrin‐peptide conjugates with different functionalities.

## 2 Results and Discussion

**Figure 2:**
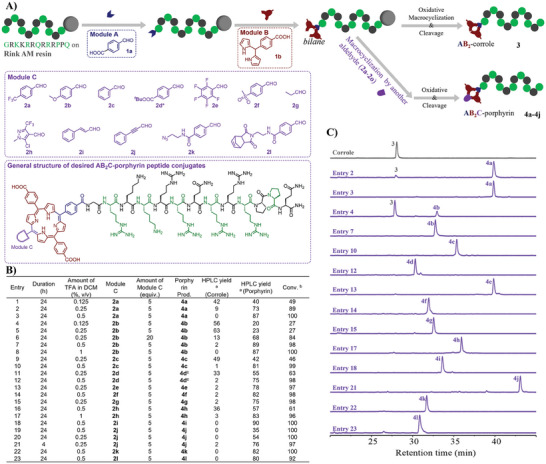
The modular construction of *trans*‐AB_2_C porphyrin on resin‐bound Tat peptide GRKKRRQRRRPPQ. A) The general synthetic approach and the synthetic building blocks (Modules A: a ‐COOH‐bearing aldehyde; Module B: a ‐COOH‐bearing dipyrromethane; Module C: an aldehyde) for constructing *trans*‐AB_2_C porphyrin on resin‐bound peptide chains; ^*^ The *^t^* Bu on **2d** was removed in the corresponding product **4d**; B) The screening and optimization of a condensation reaction between module C and bilane on‐resin, different module C and various reaction conditions were tried; ^a^ The HPLC yield of the corrole and porphyrin were estimated by analytical HPLC based on absorbance at 220 nm that is mainly contributed by peptide bonds; ^b^ The conversion in this paper, which reflect the degree of competition of condensation step between module C and on‐resin bilane, is defined as the percentage of HPLC yield of porphyrin product (**4a**‐**4k**) to the total HPLC yield of both porphyrin product (**4a**‐**4k**) and corrole product (**3**); C) Selected chromatograms of crude post‐cleavage mixtures. More detailed chromatograms for all trials can be found in Figures S1–S23 (Supporting Information).

Initially, the reaction was conducted on resin‐bound Tat peptide (GRKKRRQRRRPPQ) which is based on a commonly used cell‐penetrating peptide (CPP) derived from HIV‐1 Tat protein.^[^ ^40^ ^]^ As in the previous protocol, the 4‐formylbenzoic acid (**1a**, module A) was incorporated into the N‐terminus of the peptide at the first step. The carboxyl‐bearing dipyrromethane building block 4‐(di(1*H*‐pyrrol‐2‐yl)methyl)benzoic acid (**1b**, module B) was then condensed with an aldehyde group introduced by **1a** on the peptide in DCM with 0.125 % TFA (v/v) for 16 h to form a bilane motif on resin‐bound peptide chains. The corresponding corrole‐peptide conjugate (**3**) was obtained upon oxidation and cleavage as in the previous study.^[^ ^67^ ^]^ By condensing the bilane on resin and another aldehyde (module C) in solution phase under appropriate conditions, a porphyrinogen motif was formed by introducing the last *meso* substituent (**Figure** **2**A). We attempted to condense the electron‐withdrawing aldehyde 4‐(trifluoromethyl)benzaldehyde (**2a**) with resin‐bound bilane in DCM with different concentrations of TFA. The desired porphyrin product **4a** was detected by HPLC with a longer retention time compared with the corrole product **3** due to the introduction of another hydrophobic *meso* substituent. The HPLC yield of **4a** (estimated by absorbance at 220 nm) was 40% and 73 % after treating the bilane‐peptide conjugate on resin with 5 equiv. **2a** in DCM with 0.125 % and 0.25 % (v/v) TFA, respectively (Figure 2B, entries 1 and 2), while 42 % and 9 % of corrole **3** remained in these two trials. To evaluate the completeness of the bilane‐aldehyde condensation, we defined the percentage of desired porphyrin product in the total amount of porphyrin and corrole products as “conversion” in this study. The conversion of entries 1 and 2 were therefore calculated as 49 % and 89 %, respectively. By increasing the concentration of TFA to 0.5 %, the conversion approached 100 % as corrole **3** was not detected, and the HPLC yield of **4a** reached 87 % (Figure 2B, entry 3). For electron‐donating aldehyde 4‐methoxy benzaldehyde (**2b**), only ≈27 % conversion was observed under 0.125 % and 0.25 % of TFA in DCM (v/v) (Figure 2B, entries 4 and 5), while full conversion can be achieved when the volume ratio of TFA reached 0.5 % and 1.0 % (Figure 2B, entries 7 and 8). The unsubstituted benzaldehyde (**2c**), ester‐substituted benzaldehyde (**2d**), and 4‐(methylsulfonyl)benzaldehyde (**2f**) also required 0.5 % of TFA in DCM (v/v) to fully convert the corrole to porphyrin (Figure 2B, entries 9‐13), while the highly electron‐deficient pentafluorobenzaldehyde **2e** fully reacted in 0.25 % of TFA in DCM (v/v) (Figure 2B, entry 14). These experiments revealed that lower electron density of benzaldehyde derivatives leads to higher reactivity, as reported in literature. Interestingly, the reactivity of non‐aromatic propionaldehyde **2g** is also very high as the full conversion can be achieved in 0.25 % of TFA in DCM (v/v) (Figure 2B, entry 15). The heterocyclic aromatic example **2h** showed low reactivity, but full conversion can be still achieved in 1.0 % of TFA in DCM (v/v) (Figure 2B, entries 16 and 17). Alkene/Alkyne‐linked aldehydes **2i** and **2j** showed higher reactivity than benzaldehyde derivatives, especially **2j**, and a low TFA concentration (0.25 %) and a short reaction time (4 h) were adopted to prevent the formation of undesired products (Figure 2B, entries 18‐21).

**Figure 3:**
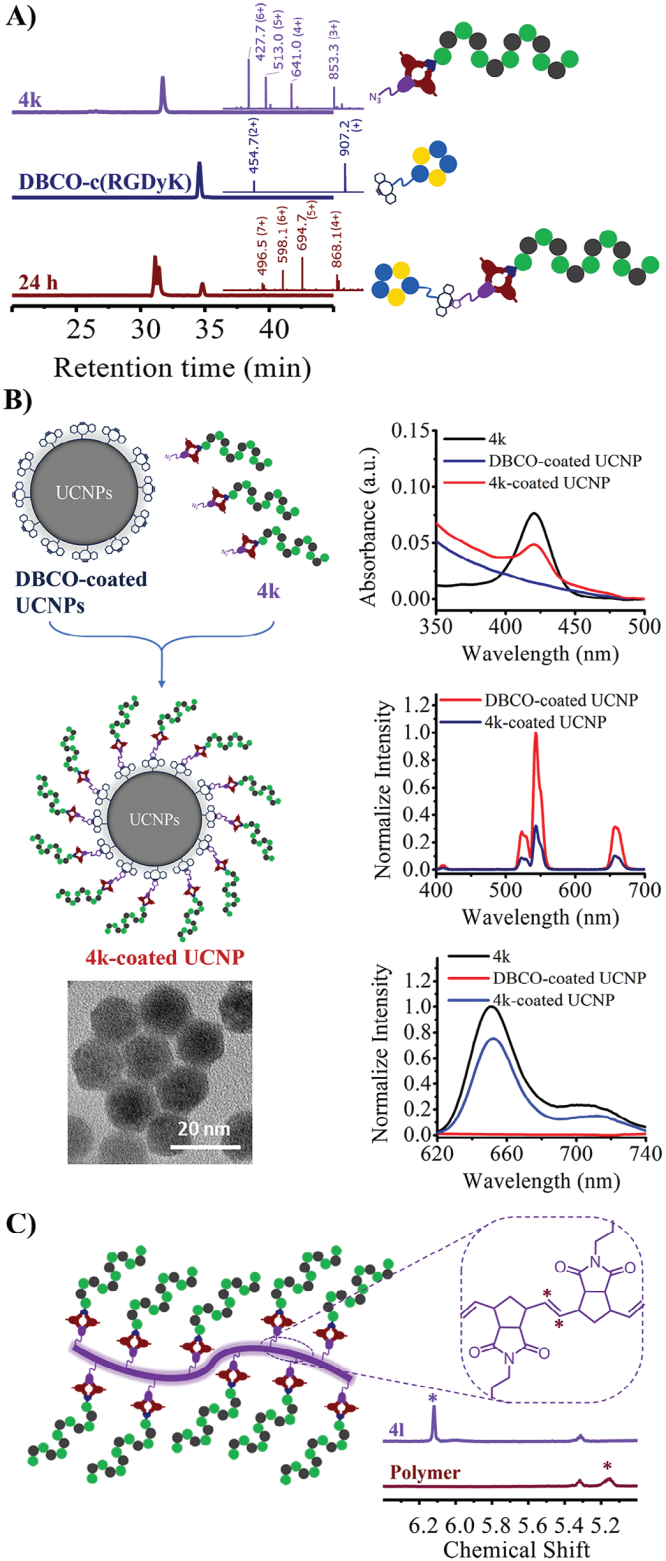
The potential application of yielded products with diverse Module C. A) Azido‐bearing **4k** was used for further conjugation via SPAAC with DBCO‐cyclo(RGDyK) conjugates; The HPLC showed the formation of clicked product after 6 h and 30 h incubation in PBS buffer; B) Azido‐bearing **4k** was used for surface modification of DBCO‐coated UCNPs. Successful surface modification was proven by finding the absorption peak and fluorescence of porphyrin in modified UCNPs. The upconversion luminescence of UCNPs(excited by 980 nm laser) was also decreased over surface modification; C) Norbornene‐bearing **4l** was used for synthesis polymer by ROMP. The change of ^1^H‐NMR spectrum indicated the successful polymerization of **4l**.

As various aldehyde building blocks have been proven to be compatible candidates as module C, we further demonstrated the potential application of their products. For example, the azido‐containing porphyrin‐peptide conjugate **4k**, which was synthesized when *N*‐(2‐azidoethyl)‐4‐formylbenzamide (**2k**) was used as module C (Figure 2B, entry 22), was expected to be a reactant for SPAAC reaction with the dibenzocyclooctyne (DBCO)‐containing counterpart. The reaction between **4k** and DBCO‐cyclo(RGDyK) conjugate **5** (αvβ3‐targeting) was investigated (**Figure** **3**A). By incubating the mixture of **4k** (1.0 mm) and **5** (1.5 mm) in phosphate buffer (10 mm, pH 7.4) at room temperature for 24 h, **4k** was completely consumed, and two Z/E isomers of the desired clicked product **4k‐cRGD** were found with similar retention times as **4k**. The conversion was confirmed complete by mass spectroscopy after isolating the fraction of the corresponding peaks at 31–32 min. This experiment demonstrated the potential of **4k**, an azido‐containing porphyrin‐peptide conjugate yielded from our synthetic methodology, for further bioconjugation or bioorthogonal^[^ ^71^, ^72^, ^73^ ^]^ application as a cancer‐targeting vector.

Moreover, the versatile **4k** was used for surface modification of DBCO‐coated upconversion nanoparticles (UCNPs)^[^ ^74^ ^]^ (Figure 3B). A suspension of **4k** (30 µg) and DBCO‐coated UCNP (5 mg) in 500 µL phosphate buffer (10 mm, pH 7.4) was stirred at room temperature for 5 h, and the successful conjugation was verified by typical absorption and emission pattern of porphyrin and reduced upconversion luminescence was observed from the colloid of fully washed UCNPs. As nanomaterials coated with both targeting motifs and photosensitizers/dyes are proven as multifunctional “smart” nanomaterials in many previous studies,^[^ ^75^, ^76^, ^77^, ^78^ ^]^ our approach provides a quick route to such materials. The norbornene‐containing product **4l** was also synthesized when a norbornene‐containing aldehyde (**2l**) was employed as module C (Figure 2B, entry 23)**. 4l** was tried for ring‐opening metathesis polymerization (ROMP) by incubating with the 2nd generation Grubbs catalyst (10 % mol) in DMF overnight under inert atmosphere (Figure 3C). Successful polymerization was proven by ^1^H‐NMR as the signal of the norbornene alkene shifted from ≈6.1 to ≈5.2 ppm, same as in previous studies.^[^ ^79^, ^80^ ^]^ As peptide‐based polymers have been used in many previous studies as biocompatible materials with good cellular permeability and higher protease resistance, this result also indicates the products from our synthetic approach can be used for making biocompatible multifunctional polymers directly.^[^ ^81^, ^82^ ^]^

**Figure 4:**
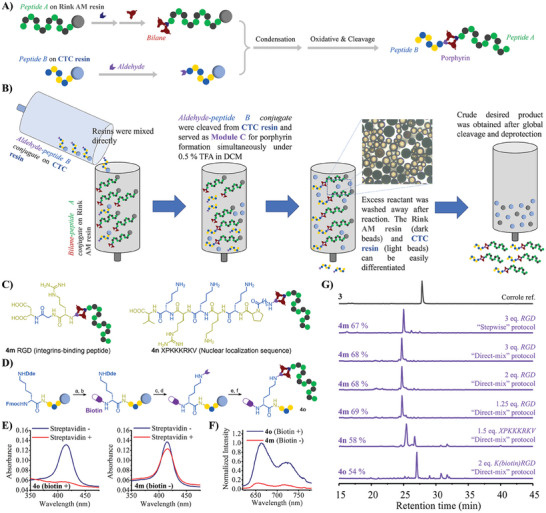
Peptide “ligation” by modular construction of *trans*‐AB_2_C porphyrin on resin‐bound peptide. A) The general synthetic strategy of peptide ligation: The aldehyde‐functionalized peptide B on CTC resin can serve as module C for constructing *trans*‐AB_2_C porphyrin. B) “Direct‐mix“protocol: The aldehyde‐functionalized peptide B on CTC resin and bilane‐bearing peptide A on Rink AM resin is mixed directly, and the cleavage of aldehyde‐functionalized peptide B from CTC resin (as module C) and the condensation of module C and bilane motif on peptide A can be conducted in the same condition. C) The structure of ligated products with different peptide B; D) Orthogonally protected lysine was incorporated to accommodate an additional biotin motif over peptide ligation to give biotin‐bearing ligated porphyrin‐peptide conjugates with two different peptide chains (**4o**); E) The change of absorption spectra of supernatants upon incubating of **4o** (2 µm, biotin +) and **4m** (2 µm, biotin ‐) with Streptavidin‐coated magnetic bead; F) The comparison of emission spectrum of suspension of Streptavidin‐coated magnetic bead after incubating with **4o** and **4m**; G) Chromatograms of crude post‐cleavage mixtures of ligated porphyrin‐peptide conjugates. More detailed chromatograms for all trials can be found in Figures S24–S29 (Supporting Information).

We further tried to apply this protocol to fuse two peptides along with the formation of an AB_2_C porphyrin by employing a side‐chain protected aldehyde‐peptide conjugate as module C (**Figure** **4**A). As the peptide chains on 2‐chlorotrityl chloride (CTC) resin can be cleaved by mildly acidic conditions, such as 0.5 %–1.0 % TFA in DCM (v/v) or 20 % HFIP in DCM (v/v), the permanent protecting groups on side chains (e.g., *^t^* Bu, Boc, Pbf, etc.) and newly attached aldehyde group on N‐terminus remain unaffected. The protected aldehyde‐RGD peptide conjugate was obtained by incorporating **1a** on the N‐terminus of RGD peptide on CTC resin followed by treating the washed resin with 20 % HFIP in DCM (v/v). The structure and purity of post‐cleavage protected aldehyde‐RGD peptide conjugate [**1a**‐Arg(Pbf)‐Gly‐Asp(^t^Bu)‐OH] were confirmed by NMR spectra (Figures S38–S42, Supporting Information) after drying under vacuum overnight. The dried **1a**‐Arg(Pbf)‐Gly‐Asp(^t^Bu)‐OH (3 equiv.) was then used as module C directly without purification. As a result, complete conversion was achieved after 24 h of condensation to give a new product with a shorter retention time than corrole‐peptide conjugate **3** due to the introduction of a hydrophilic RGD peptide. Interestingly, as 0.5 % TFA in DCM (v/v) is a suitable condition for both cleavage of CTC resin and condensation between bilane and aldehyde, we therefore tried a “direct‐mix” protocol by directly mixing a CTC resin‐bound aldehyde‐peptide conjugate with a bilane‐peptide conjugate on Rink amide resin (Figure 4B) in 0.5 % TFA in DCM (v/v) for 24 h. Surprisingly, a similar HPLC yield can be achieved by using the same amount of aldehyde‐RGD peptide conjugate (67 % and 70 % for the formerly mentioned stepwise protocol and ‘‘direct‐mix protocol). To reduce the consumption of peptide B, we also tried to reduce the amount of CTC resin‐bound RGD peptide to 2 equiv. and 1.25 equiv., and complete conversion was still achieved. In addition, NLS peptide XPKKKRKV (X refer to 6‐Aminohexanoic Acid, Ahx) was tried as an alternative peptide B by ‘‘direct‐mix protocol, which also gives a 58 % HPLC yield. These examples elucidated that our synthetic approach is not limited to yield *trans‐*AB_2_C porphyrin‐peptide conjugates with additional small functional group brought by simple module C. By recruiting aldehyde‐peptide conjugates, we achieved a “peptide ligation” ‐like multifunctionalization by fusing two peptides together with a newly formed porphyrin motif. To the best of our knowledge, we are also reporting the first example to functionalize peptide by directly mixing their protected form on solid support.

We further extended the application of this “resin‐to‐resin” ligation by introducing an additional functional motif in addition to a native peptide B (Figure 4E). In other words, peptide B was not only a ligated peptide but also a carrier for other functional motifs, like drug molecules. By incorporating an orthogonally protected amino acid Fmoc‐Lys(Dde)‐OH onto CTC resin‐bound RGD peptide, two amine groups of the lysine can be functionalized with different motifs. The Fmoc protecting group was removed to release its α‐amine, and a ‐COOH‐containing drug building block was installed. The Dde protecting group on the side‐chain amine group was then removed for deploying aldehyde building block **1a**, such a bisfunctionalized peptide on CTC resin therefore becomes a feasible carrier for incorporating additional motifs as the module C of porphyrin. We used biotin as an example in this experiment to give the desired product **4o** which bears a porphyrin, two different peptide chains, and a biotin motif. As the biotin motif is well‐known to be a strong ligand for Avidin or Streptavidin, **4o** and **4m** were incubated with a Streptavidin‐coated magnetic bead (Dynabeads MyOne Streptavidin C1, Thermo Fisher Scientific) to see the change of absorbance in their supernatants. As a result, the absorption peak of porphyrin of **4o** disappeared after incubation, while that of **4m** only slightly decreased, which demonstrated the function of biotin motif in **4o** (Figure 4F). The fluorescence of the porphyrin motif from the fully washed bead also showed significantly different intensity as more **4o** was attached to the Streptavidin on bead by its biotin motif (Figure 4G).

## 3 Conclusion

In this study, we developed a modular solid‐phase synthetic approach that directly assembled porphyrin on peptides by orderly introducing three simple modules. A series of *trans*‐AB_2_C porphyrin‐peptide conjugates were obtained by this approach without preparing porphyrin building blocks in advance. The diverse module C imparts various additional functionalities on the desired products easily, demonstrating potential application in different fields like bioorthogonal chemistry, surface modification of nanomaterial, and preparation of peptide‐based polymers. Moreover, by using CTC‐resin‐bound aldehyde‐peptide conjugates as module C, two peptides were successfully linked together by a newly formed porphyrin motif, which offers the functionalities of both porphyrin and peptide B (as well as the additional functional motif on peptide B) to peptide A by a simple “resin‐to‐resin” reaction.

## Conflict of interest

The authors declare no conflict of interest.

## Supporting information



Supporting Information

## Data Availability

The data that support the findings of this study are available in the supplementary material of this article.
