# Supplementary material for: Peptide Multifunctionalization via Modular Construction of Trans‐AB2C Porphyrin on Resin
Source: Adv Sci (Weinh). 2025 Feb 19;12(14):2409771. doi: 10.1002/advs.202409771 (PMC11984925; doi:10.1002/advs.202409771)
Supplement: Supplementary file 1 — Supporting Information [file ADVS-12-2409771-s001.pdf]

ADVANCED SCIENCE
Open Access

Supporting Information

for *Adv. Sci.*, DOI 10.1002/advs.202409771

Peptide Multifunctionalization via Modular Construction of *Trans*-AB_2C Porphyrin on Resin

Yue Wu, Yuen-Ting Wong, Yik-Hoi Yeung, Pak-Lun Lam, Ho-Fai Chau, Wing-Sze Tam, Qian Zhang, William C. S. Tai and Ka-Leung Wong**

Content

Experiments	S2-S36
References	S37
NMR spectra of products	S38-S44
HPLC chromatograms and MALDI-TOF HRMS spectra of products	S45-S60

Experiments

Reagents. All amino acid building blocks, the coupling reagent PyBOP (benzotriazol-1-yloxytripyrrolidinophosphonium hexafluorophosphate), and 2-chlorotrityl chloride (CTC) resin were purchased from Bidepharm. The Rink AM resin was purchased from GLBioChem. All solvents and other commonly used reagents were purchased and used without further purification.

Analytical HPLC. Analytical HPLC was performed on an Agilent 1100 series HPLC system (Agilent Technologies, Stockport, UK) equipped with a diode-array detection (DAD) detector and an Agilent C18 column (250 mm x 4.6 mm, particle size: 5 µm). Mobile phases: A, H_2O + 0.1 % TFA; B: MeCN + 0.1 % TFA. Flow rate: 0.5 mL/min. Gradient: 0 min, 10 % B; 40 min, 60 % B; 41 min, 100 % B; 55 min, 100 % B. For the post-cleavage mixture, the absorbance at 220 nm, mainly contributed by peptide bonds, was used to estimate the relative abundance of each composition. The absorbance at 420 nm (mainly contributed by corrole or porphyrin products) was also used for the preliminary identification of formed tetrapyrrolic products from the post-cleavage mixture. The purity of the purified products was reported by the absorbance at 220 nm.

Preparative HPLC. The purifications of crude products were carried out on Waters semi-preparative system with Waters 2707 Autosampler, Water 1525 Binary HPLC Pump, Waters 2998 Photodiode Array Detector, Waters Fraction Collector III and Atlantis® T3 Prep OBD™ C18 column (250 mm x 19 mm, particle size: 5 µm). As the length and particle size of the analytical column and the preparative column are identical, the same gradient was usually used as its on analytical HPLC but with a flow rate of 8.53 mL/min which calculated from the ratio of base area of them. The retention time of the desired products was usually similar on preparative HPLC as on analytical HPLC. The fractions of the desired product were collected and verified by ESI-MS.

Mass spectrometry. High-resolution mass spectra, reported as m/z, were obtained from Bruker Autoflex MALDI-TOF mass spectrometer. Low-resolution mass spectra, which were conducted by SCIEX 3200Q ESI mass spectrometer, were also used for reaction monitoring and fractions determination during the purification.

Nuclear magnetic resonance. NMR spectra were recorded on a Bruker Ultrashield 400 Plus NMR spectrometer (^{1}H NMR on 400 MHz, ^{13}C NMR on 101 MHz. The ^{1}H NMR (2.50 for DMSO-d_6) and ^{13}C NMR (39.52 for DMSO-d_6) chemical shifts were referenced to the corresponding solvent peak. The following abbreviations were used to explain the multiplicities: s = singlet, d = doublet, t = triplet, q = quartet, dd = doublet of doublets, m = multiplet, br = broad.

The general procedure of solid-phase peptide synthesis. Standard Fmoc-SPPS was carried out manually in an empty SPE tube with frits. The Rink AM resin (0.5 – 1.0 mmol) was used for Tat peptide (peptide A) in this study. To load the first amino acid onto Rink AM resin, Fmoc-deprotected resin was shaken with amino acid building block (4 equiv.), PyBOP (4 equiv.), and DIPEA (8 equiv.) in DMF (4 mL/0.1 mmol) overnight. Also, the CTC resin (0.5 – 1.0 mmol) was used for all peptide B in this study. To load the first amino acid onto CTC resin, the resin was shaken in the solution of amino acid building block (4 equiv.) and DIPEA (8 equiv.) in anhydrous DCM (10 mL/g resin) for 30 mins, the resin was then washed with DMF, DCM, and 50% MeOH in DCM sequentially followed by shaking in a mixture of DCM/MeOH/DIPEA, v/v/v, 17/2/1 for one hour. During peptide elongation, the resin was shaken with the amino acid building block (4 equiv.), PyBOP (4 equiv.) and DIPEA (8 equiv.) in DMF (4 mL/0.1 mmol)

for 2 – 16 h. To remove Fmoc protecting group during SPPS, the resin was shaken with a solution of 20 % 4-methylpiperidine in DMF (v/v) for 25 min. The resin was fully washed (~ 4 ml/0.1 mmol, 3 – 4 times) with DMF after every single step. Before removing the last Fmoc protecting group, the resin was fully washed with DMF and DCM, and diethyl ester. The resin was fully dried under vacuum and the substitution value of resin-bound peptide was determined by the method from literature.[1]

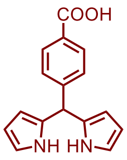

Preparation 4-(di(1*H*-pyrrol-2-yl)methyl)benzoic acid (1b) as Module B. To a mixture of 4-formylbenzoic acid (**1a**, 1 equiv.) and pyrrole (30 equiv., served as solvent), $InCl_3$ was added (0.1 equiv., served as catalyst). The resulting mixture was stirred at r.t. for around 120 min. Once **1a** was no longer detected by TLC, excess pyrrole was allowed to be removed under vacuum. The residue was then purified by flash column chromatography on silica gel to give the desired dipyrromethene building block. Yield 73 %; Grey powder; ^{1}H NMR (400 MHz, DMSO-*d*$_6$) δ 12.84 (s, 1H), 10.62 (s, 2H), 7.87 (d, *J* = 8.3 Hz, 2H), 7.27 (d, *J* = 8.1 Hz, 2H), 6.63 (q, *J* = 2.3 Hz, 2H), 5.92 (q, *J* = 2.7 Hz, 2H), 5.68 (dd, *J* = 3.7, 2.1 Hz, 2H), 5.44 (s, 1H); ^{13}C NMR (101 MHz, DMSO-*d*$_6$) δ 167.3, 148.9, 132.4, 129.3, 128.7, 128.3, 117.1, 107.0, 106.3, 43.4.

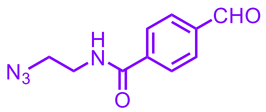

Preparation of *N*-(2-azidoethyl)-4-formylbenzamide (azido-containing building block, 2k) as Module C. A mixture of 2-azidoethan-1amine (2.4 mmol, 1 equiv., prepared in our previous study[2]) 4-formylbenzaldehyde (**1a**, 3 mmol, 1.25 equiv.), HATU (2.4 mmol, 1 equiv.) and DIPEA (3 mmol, 1.25 equiv.) in 25 mL DMF was allowed to stir overnight at r.t.. The reaction mixture was concentrated by rotary evaporator to reduce the amount of DMF, followed by adding large excess water. The mixture was then extracted with ethyl acetate for three times. The combined organic layer was washed by saline and dried by anhydrous Na_2SO_4. The solvent was removed by rotary evaporator. The compound was purified by flash column chromatography with hexane and ethyl acetate, followed by reprecipitation by hexane. Yield 51%; White powder; ^{1}H NMR (400 MHz, $CDCl_3$) δ 10.08 (s, 1H), 7.98 – 7.91 (m, 4H), 6.56 (s, 1H), 3.69 – 3.64 (m, 2H), 3.62 – 3.57 (m, 2H); ^{13}C NMR (101 MHz, $CDCl_3$) δ 191.60, 166.73, 139.30, 138.50, 130.05, 127.83, 50.96, 39.74.

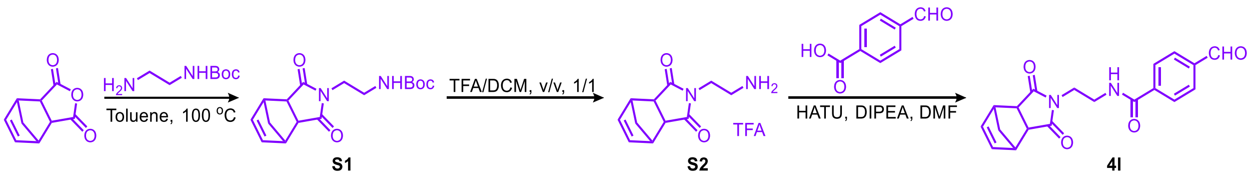

Scheme S1. Synthetic route for **4l**.

Preparation of *N*-(2-(1,3-dioxo-1,3,3a,4,7,7a-hexahydro-2*H*-4,7-methanoisoindol-2-yl)ethyl)-4-formylbenzamide (norbornene-containing building block, 2l) as Module C. The building block **2l** was synthesized as the route on **Scheme S1**. A suspension of isobenzofuran-1,3-dione (1.64 g, 10 mmol) and N-Boc-ethylenediamine (1.76 g, 11 mmol) in toluene (30 mL) was refluxed overnight. The solvent was removed by rotary evaporation, and the residue was redissolved in DCM, followed by washing with saturated NH_4Cl aqueous solution three times. The resulting organic layer was dried by Na_2SO_4, followed by filtration. The filtrate was concentrated to around 10 mL, then, 100 mL hexane was added to form white precipitate. The precipitate was collected by filtration and dried under vacuum to give *tert*-butyl (2-(1,3-dioxo-1,3,3a,4,7,7a-hexahydro-2*H*-4,7-methanoisoindol-2-yl)ethyl)carbamate (**S1**). Yield 79 %. White powder. ^{1}H NMR (400 MHz, $CDCl_3$) δ 6.11 (t, *J* = 1.9 Hz, 2H), 4.66 (s, 1H), 3.48 (t, *J* = 5.7 Hz, 2H), 3.38 (dp, *J* = 3.4, 1.7 Hz, 2H), 3.26 (dd, *J* = 2.9, 1.6 Hz, 2H), 3.22 (q, *J* = 5.5 Hz, 2H), 1.73 (dt, *J* = 8.8, 1.7 Hz, 1H), 1.54 (dt, *J* = 8.8, 1.5 Hz, 1H), 1.41 (s, 9H); ^{13}C NMR (101 MHz, $CDCl_3$) δ 177.9, 155.7, 134.5, 79.4,

52.2, 45.8, 44.9, 39.0, 37.8, 28.4. The **S1** (1.53g, 5 mmol) was further dissolved a 20 mL solution of TFA/DCM, v/v, 1/1 and stirred for 4 h. Upon completion, 10 mL toluene was added, and all solvent was removed by rotary evaporation. After drying the residue completely under vacuum, a solution of 4-formylbenzoic acid (751 mg, 5 mmol), PyBOP (2.60 g, 5 mmol) and DIPEA (2.6 mL, 15 mmol) in 15 mL DMF was added and allowed to stir at r.t. overnight. After that, the reaction mixture was concentration by rotary evaporation, followed by adding excess water and extracting with DCM three times. The combined organic layer was washed with brine three times, followed by drying with Na_2SO_4. The dried organic layer was concentrated and purified by column chromatography to give **4l** as final product. Yield 69 %. White powder. ^{1}H NMR (400 MHz, DMSO-d_6) δ 10.07 (s, 1H), 8.67 (t, J = 5.8 Hz, 1H), 8.04 – 7.91 (m, 4H), 5.99 (s, 2H), 3.43 (t, J = 6.1 Hz, 2H), 3.34 – 3.28 (m, 4H), 3.20 (dq, J = 3.2, 1.6 Hz, 2H), 1.53 (t, J = 1.6 Hz, 2H); ^{13}C NMR (151 MHz, DMSO-d_6) δ 192.9, 177.5, 165.6, 139.5, 137.8, 134.3, 129.5, 127.9, 51.7, 45.4, 44.2, 37.1, 37.0.

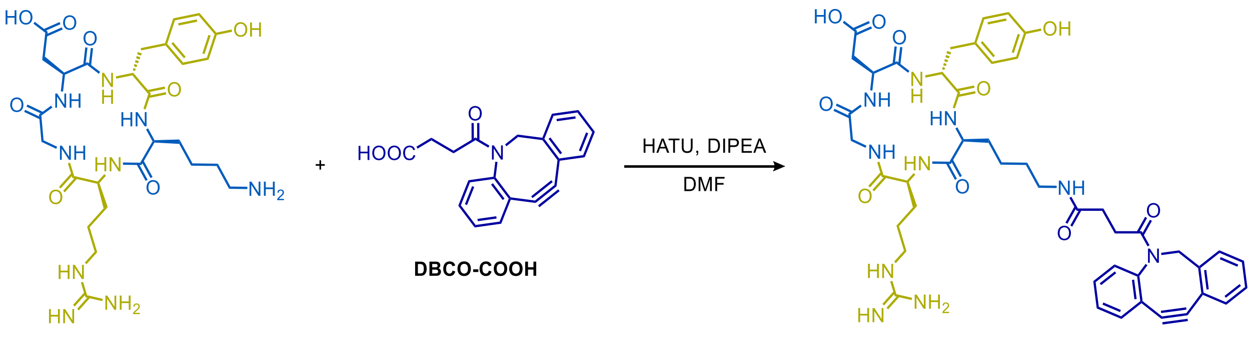

Scheme S2. Synthetic route for **DBCO-cRGD**.

Preparation of DBCO-cyclo(RGDyK) conjugate (DBCO-cRGD). As shown on **Scheme 2**, a mixture of cyclo(RGDyK) peptide (0.1 mmol, 1 equiv., with a free amine on the side chain of lysine), DBCO-COOH (0.1 mmol, 1 equiv.), HATU (0.1 mmol, 1 equiv.) and DIPEA (0.3 mmol, 3 equiv.) in 1 mL DMF was allowed to stir for 4 h at r.t.. The reacting mixture was purified by preparative HPLC to give a purified **DBCO-cRGD**. Yield 39 %. White powder. Analytic HPLC: retention time: 34.6 min, purity: 94.1 %. HRMS(MALDI-TOF): calc. for $C_{46}H_{54}N_{10}O_{10}^+$ $[M+H]^+$ 907.4097, found 907.4737.

The general procedure to modularly construct *trans*-AB$_2$C porphyrin on resin-bound peptides. The resin-bound Tat peptide (GRKKRRQRRRPPQ, 0.01-0.05 mmol) was placed into a 10 mL syringe with frit (http://www.torviq.com/) and swelled in DMF for 5-30 min. The Fmoc protecting group was removed as the protocol in the general procedure of solid-phase peptide synthesis. Module A was incorporated by standard coupling protocol with a solution of 4-formylbenzoic acid (**1a**, 5 equiv.), PyBOP (5 equiv.), and DIPEA (10 equiv.) in DMF (~ 1 ml/0.02 mmol) for 6 h. After installation of Module A, the resin was fully washed with DMF, then was further flushed with DCM three times. The resin was fully dried by compressed air, and the powder of Module B (**1b**, 5 equiv.) was added into syringe to directly mix with dried resin. A solution of TFA (0.25 %, v/v) in DCM (~ 1 ml/0.02 mmol) was put into syringe, and the syringe was allowed to shake in dark overnight (~ 16 h). The color of resin turned from pale yellow to brown after installation of Module B, the resin was fully washed with DMF, then was further flushed with DCM and fully dried by compressed air, and a solution of Module C (**2a**–**2l**, 5 equiv.) in DCM (~ 1 ml/0.02 mmol resin) containing 0.25 - 1 % TFA (v/v) was put into syringe to mix with dried resin, and the syringe was shaken in dark for 24 h , unless otherwise specified. The color of resin turned from brown to red during the installation of Module C. Upon completion, the resin was fully washed with NMP, and a solution of p-

chloranil (10 equiv.) in NMP (~ 1 ml/0.02 mmol) was put into syringe for oxidation step. The syringe was fully washed with DMF after 3 h shaking in dark, then was further flushed with DCM. The resin was fully dried by compressed air, and the global cleavage and deprotection was carried out with a fresh-prepared cleavage cocktail (TFA/TIPS/H_2O, v/v/v, 95/2.5/2.5) for 3-4 h. The post-cleavage mixture was collected in centrifuge tubes, and the resin was washed several more times with fresh-prepared cleavage cocktail. The excess diethyl ester or methyl *tert*-butyl ether (over 10/1, v/v) was added into combined post-cleavage solutions, and the formed precipitate was collected by centrifugation (10000 rpm, 5 min). The precipitate was re-dissolved in methanol, then repeated precipitation and centrifugation for one more time. The crude product was re-dissolved in H_2O/MeCN and analyzed by analytical HPLC (**Figure S1**–**S21**). The absorbance at 220 nm, which is mainly contributed by peptide bonds, was used for estimating the HPLC yield of each composition in post-cleavage mixtures. The absorbance at 420 nm (corrole or porphyrin products) was also used for preliminary identification of formed products. The desired products were isolated by preparative HPLC, and the purity was reported by analytical HPLC with absorbance at 220 nm.

4a. 4-(Trifluoromethyl)benzaldehyde (**2a**, 5 equiv.) was used as Module C. 0.125 %, 0.25 %, and 0.5 % of TFA in DCM (v/v) were tried as solvent in *Entry 1–3* (HPLC chromograms are presented on **Figure S1–S3**) on **Figure 1B**, respectively. Isolated yield: 11 % (From *Entry 3*). Green powder. Analytic HPLC: retention time: 39.8 min, purity: 98.5 %. HRMS(MALDI-TOF): calc. for $C_{118}F_3H_{160}N_{40}O_{20}^+$ $[M+H]^+$ 2514.2679, found 2514.2962.

4b. 4-Methoxybenzaldehyde (**2b**) was used as Module C. Various conditions were tried in *Entry 4–8* (HPLC chromograms are presented on **Figure S4–S8**) on **Figure 1B** as following: *Entry 4,* 5 equiv. **2b**, 0.125 % TFA in DCM (v/v); *Entry 5,* 5 equiv. **2b**, 0.25 % TFA in DCM (v/v); *Entry 6,* 20 equiv. **2b**, 0.25 % TFA in DCM (v/v); *Entry 7,* 5 equiv. **2b**, 0.5 % TFA in DCM (v/v); *Entry 8,* 5 equiv. **2b**, 1.0 % TFA in DCM (v/v). Isolated yield: 12 % (From combined post-cleavage mixture of *Entry 7* and *8*). Green powder. Analytic HPLC: retention time: 32.9 min, purity: 97.1 %. HRMS(MALDI-TOF): calc. for $C_{118}H_{163}N_{40}O_{21}^+$ $[M+H]^+$ 2476.2911, found 2476.3781.

4c. Benzaldehyde (**2c**, 5 equiv.) was used as Module C. 0.25 % and 0.5 % of TFA in DCM (v/v) were tried as solvent in *Entry 9* and *10* (HPLC chromograms are presented on **Figure S9** and **S10**) on **Figure 1B**, respectively. Isolated yield: 12 % (From *Entry 10*). Green powder. Analytic HPLC: retention time: 35.5 min, purity: 96.1 %. HRMS(MALDI-TOF): calc. for $C_{117}H_{161}N_{40}O_{20}^+$ $[M+H]^+$ 2446.2805, found 2446.3972.

4d. *Tert*-butyl 4-formylbenzoate (**2d**, 5 equiv.) was used as Module C. 0.25 % and 0.5 % of TFA in DCM (v/v) were tried as solvent in *Entry 11* and *12* (HPLC chromograms are presented on **Figure S11** and **S12**) on **Figure 1B**, respectively. Isolated yield: 12 % (From *Entry 12*). Green powder. Analytic HPLC: retention time: 30.4 min, purity: 96.9 %. HRMS(MALDI-TOF): calc. for $C_{118}H_{161}N_{40}O_{22}^+$ $[M+H]^+$ 2490.2704, found 2490.3673.

4e. 2,3,4,5,6-Pentafluorobenzaldehyde (**2e**, 5 equiv.) was used as Module C. 0.25 % TFA in DCM (v/v) was tried as solvent in *Entry 13* (HPLC chromograms are presented on **Figure S13**) on **Figure 1B**. Isolated yield: 13 %. Green powder. Analytic HPLC: retention time: 39.4 min, purity: 97.9 %. HRMS(MALDI-TOF): calc. for $C_{117}F_5H_{156}N_{40}O_{20}^+$ $[M+H]^+$ 2536.2334, found 2536.3172.

4f. 4-(Methylsulfonyl)benzaldehyde (**2f**, 5 equiv.) was used as Module C. 0.5 % TFA in DCM (v/v) was tried as solvent in *Entry 14* (HPLC chromograms are presented on **Figure S14**) on **Figure 1B**. Isolated yield: 11 %. Green powder. Analytic HPLC: retention time: 31.9 min, purity: 98.5 %. HRMS(MALDI-TOF): calc. for $C_{118}H_{163}N_{40}O_{22}S^+$ $[M+H]^+$ 2524.2581, found 2524.3340.

4g. Propionaldehyde (**2g**, 5 equiv.) was used as Module C. Isolated yield: 14 %. 0.25 % TFA in DCM (v/v) was tried as solvent in *Entry 15* (HPLC chromograms are presented on **Figure S15**) on **Figure 1B**. Green

powder. Analytic HPLC: retention time: 32.5 min, purity: 98.8 %. HRMS(MALDI-TOF): calc. for $C_{113}H_{161}N_{40}O_{20}^+$ $[M+H]^+$ 2398.2805, found 2398.3825.

4h. 5-Chloro-1-methyl-3-(trifluoromethyl)-1*H*-pyrazole-4-carbaldehyde (**2h**, 5 equiv.) was used as Module C. 0.5 % and 1.0 % of TFA in DCM (v/v) were tried as solvent in *Entry 16* and *17* (HPLC chromograms are presented on **Figure S16** and **S17**) on **Figure 1B**, respectively. Isolated yield: 10 % (From *Entry 17*). Green powder. Analytic HPLC: retention time: 35.8 min, purity: 97.3 %. HRMS(MALDI-TOF): calc. for $C_{116}ClF_3H_{159}N_{42}O_{20}^+$ $[M+H]^+$ 2552.2351, found 2552.3151.

4i. Cinnamaldehyde (**2i**, 5 equiv.) was used as Module C. 0.5 % TFA in DCM (v/v) was tried as solvent in *Entry 18* (HPLC chromograms are presented on **Figure S18**) on **Figure 1B**. Isolated yield: 15 %. Green powder. Analytic HPLC: retention time: 33.6 min, purity: 97.0 %. HRMS(MALDI-TOF): calc. for $C_{119}H_{163}N_{40}O_{20}^+$ $[M+H]^+$ 2472.2962, found 2472.3855.

4j. 3-Phenylpropiolaldehyde (**2j**, 5 equiv.) was used as Module C. 0.5 % and 0.25 % of TFA in DCM (v/v) were tried as solvent in *Entry 19* and *20* (HPLC chromograms are presented on **Figure S19** and **S20**) on **Figure 1B**, respectively. As large number of undesired products were formed in *Entry 19* and *20*, shorter reacting time (4 h) was tried in *Entry 21* to give an improved result (**Figure S23**). Isolated yield: 10 % (From *Entry 17*). Green powder. Analytic HPLC: retention time: 43.1 min, purity: > 99 %. HRMS(MALDI-TOF): calc. for $C_{119}H_{161}N_{40}O_{20}^+$ $[M+H]^+$ 2470.2805, found 2470.3538.

4k. *N*-(2-azidoethyl)-4-formylbenzamide (**2k**) was used as Module C. 0.5 % TFA in DCM (v/v) was tried as solvent in *Entry 22* (HPLC chromograms are presented on **Figure S22**) on **Figure 1B**. Isolated yield: 11 %. Green powder. Analytic HPLC: retention time: 31.3 min, purity: 98.9 %. HRMS(MALDI-TOF): calc. for $C_{120}H_{165}N_{44}O_{21}^+$ $[M+H]^+$ 2558.3191, found 2558.4107.

4l. *N*-(2-(1,3-dioxo-1,3,3a,4,7,7a-hexahydro-2*H*-4,7-methanoisoindol-2-yl)ethyl)-4-formylbenzamide (**2l**) was used as Module C. 0.5 % TFA in DCM (v/v) was tried as solvent in *Entry 23* (HPLC chromograms are presented on **Figure S23**) on **Figure 1B**. Isolated yield: 10 %. Green powder. Analytic HPLC: retention time: 30.8 min, purity: > 99 %. HRMS(MALDI-TOF): calc. for $C_{129}H_{173}N_{42}O_{23}^+$ $[M+H]^+$ 2678.3653, found 2678.4548.

The general procedure to conduct peptide ligation by modularly construct *trans*-AB$_2$C porphyrin on resin-bound peptide. The installation of Module A and Module B on Rink AM resin-bound peptide A are identical with the general procedure to modularly construct trans-AB$_2$C porphyrin on resin-bound peptides. A CTC resin-bound aldehyde-functionalized peptide B was prepared as Module C (1.25 – 5 equiv.) for peptide ligation. Briefly, after completion of SPPS for peptide B on CTC resin, 4-formylbenzoic acid was incorporated by standard coupling protocol onto the N-terminal of peptide B with a solution of 4-formylbenzoic acid (**1a**, 5 equiv.), PyBOP (5 equiv.), and DIPEA (10 equiv.) in DMF (~ 1 ml/0.02 mmol) for 3-16 h. The resin was fully washed with DMF, then was further flushed with DCM and fully dried by compressed air. Two following protocols were tried for applying aldehyde-functionalized peptide B as Module C:

"Stepwise" protocol: The aldehyde-functionalized peptide B was cleaved from CTC resin by shaking with solution of 20 % HFIP in DCM (v/v, ~ 1 ml/0.02 mmol) for 20 min. Upon completion, the solution phase was collected, and the resin was fully washed by 20 % HFIP in DCM, and the combined solution phase was vaporized under airflow and vacuum to get crude aldehyde-peptide B conjugate. As the solubility of aldehyde-peptide B conjugates in DCM were usually not good, most of dried powder of aldehyde-peptide B conjugates were therefore transferred into syringe to directly mix with dried resin of bilane-grafted peptide A. The remaining powder of aldehyde-peptide B conjugates was dissolved in a solution of TFA (0.5 - 1 %, v/v) in DCM (~ 1 ml/0.02 mmol) and put into syringe.

"Direct-mix" protocol: The aldehyde-functionalized peptide B on CTC resin was transferred into another syringe to directly mix with dried resin of bilane-grafted peptide A. DMF and DCM may be used to facilitate the transfer of resin. The mixed resin was further flushed with DCM and fully dried by compressed air. A solution of TFA (0.5 - 1 %, v/v) in DCM (~ 1 ml/0.02 mmol) and put into syringe.

The syringe was further shaken in dark for 24 h. and all following steps are identical with the *general procedure to modularly construct trans-AB$_2$C porphyrin on resin-bound peptide*s.

4m. H-Arg(Pbf)-Gly-Asp(^{t}Bu) (RGD) on CTC resin was used Peptide B. Isolated yield: 9 %. Green powder. Analytic HPLC: retention time: 24.8 min, purity: 98.2 %. HRMS(MALDI-TOF): calc. for C132H188N31O26+ [M+H]+ 2623.4336, found 2623.5154.

4n. H-Ahx-Pro-Lys(Boc)-Lys(Boc)-Lys(Boc)-Arg(Pbf)-Lys(Boc)-Val (XPKKKRKV) on CTC resin was used Peptide B. Isolated yield: 8 %. Green powder. Analytic HPLC: retention time: 25.5 min, purity: 98.2 %. HRMS(MALDI-TOF): calc. for C164H248N55O30+ [M+H]+ 3467.9566, found 3468.1242.

The procedure to functionalize peptide with drug motif by directly ligation between two resin-bound peptides during modular construction of *trans*-AB$_2$C porphyrin on resin-bound peptides. Fmoc-Lys(Dde)-OH was incorporated onto Peptide B (RGD as an example here) on CTC resin first. The Fmoc protecting group was remove to couple a -COOH bearing drug molecule (Biotin as an example) by standard coupling protocol with a solution of biotin (5 equiv.), PyBOP (5 equiv.), and DIPEA (10 equiv.) in DMF (~ 1 ml/0.02 mmol) for 3 h. The Dde protecting group was then removed by shaking with 2 % $NH_2NH_2 \cdot H_2O$ in DMF (v/v) for 3 min × 3. The CTC resin was fully washed with DMF, and the **1a** was incorporated by standard coupling protocol with a solution of 4-formylbenzoic acid (**1a**, 5 equiv.), PyBOP (5 equiv.), and DIPEA (10 equiv.) in DMF (~ 1 ml/0.02 mmol) for 3 h. "Direct-mix" protocol was used for applying aldehyde and drug motif-bisfunctionalized Peptide B as Module C. All following steps are identical with the general procedure to modularly construct trans-AB$_2$C porphyrin on resin-bound peptides. The product **4o** was made as an example. Isolated yield: 8 %. Green powder. Analytic HPLC: retention time: 26.9 min, purity: 93.2 %. HRMS(MALDI-TOF): calc. for $C_{146}H_{207}N_{50}O_{30}S^+$ $[M+H]^+$ 3172.5925, found 3172.7241.

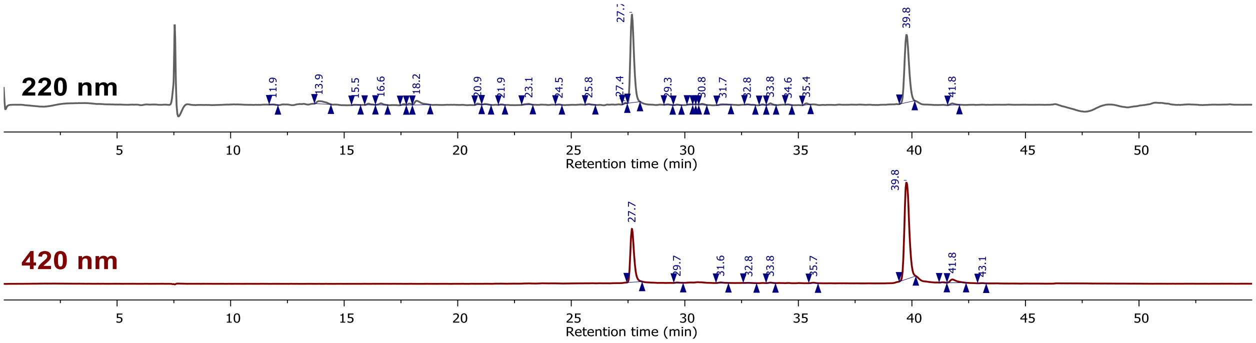

Figure S1. The HPLC chromogram of crude post-cleavage mixture of *Entry 1* on **Figure 1B** based on the absorbance at 220 nm (grey, peptide bond) and 420 nm (red, corrole/porphyrins). The composition (absorbance at 220 nm) of crude post-cleavage mixture was presented as the following table:

Peak No.	t_R (min)	Area	Area (%) [a]	Proposed product
1	41.779	1.960	0.93	
2	39.766	84.206	40.12	Desired porphyrin-peptide conjugate (**4a**)
3	35.360	1.450	0.69	
4	34.554	0.404	0.19	
5	33.767	1.586	0.76	
6	33.420	0.633	0.30	
7	32.754	0.591	0.28	
8	31.681	1.171	0.56	
9	30.754	1.248	0.59	
10	30.521	0.438	0.21	
11	30.447	0.441	0.21	
12	30.321	0.551	0.26	
13	29.654	0.528	0.25	
14	29.261	0.629	0.30	
15	27.668	88.276	42.06	Corrole-peptide conjugate (**3**)
16	27.368	0.532	0.25	
17	25.775	0.926	0.44	
18	24.468	0.413	0.20	
19	23.135	1.416	0.67	
20	21.915	0.425	0.20	
21	21.208	0.923	0.44	
22	20.882	1.010	0.48	
23	18.189	7.477	3.56	
24	17.862	1.406	0.67	
25	17.622	0.842	0.40	
26	16.622	1.839	0.88	
27	16.069	1.376	0.66	
28	15.496	0.380	0.18	
29	13.896	6.383	3.04	
30	11.870	0.400	0.19	

[a.] "Area (%)" indicates the relative abundance of each composition in the post-cleavage mixture. It is calculated by (Peak area / Total peak area of all peaks) × 100

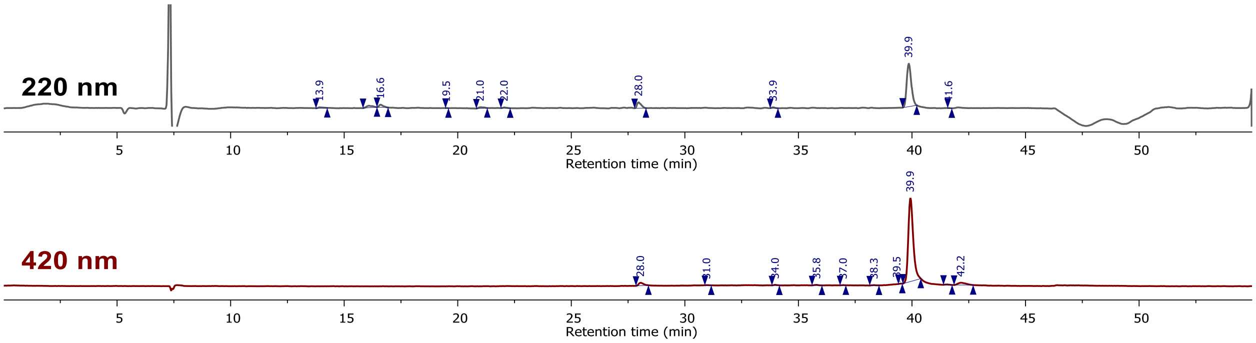

Figure S2. The HPLC chromogram of crude post-cleavage mixture of *Entry 2* on **Figure 1B** based on the absorbance at 220 nm (grey, peptide bond) and 420 nm (red, corrole/porphyrins). The composition (absorbance at 220 nm) of crude post-cleavage mixture was presented as the following table:

Peak No.	t_R (min)	Area	Area (%)	Proposed product
1	41.620	0.047	0.10	
2	39.857	36.611	75.74	Desired porphyrin-peptide conjugate (**4a**)
3	33.892	0.471	0.97	
4	27.967	4.575	9.47	Corrole-peptide conjugate (**3**)
5	22.049	0.460	0.95	
6	20.993	1.172	2.42	
7	19.504	0.046	0.09	
8	16.605	1.935	4.00	
9	16.077	2.258	4.67	
10	13.920	0.760	1.57	

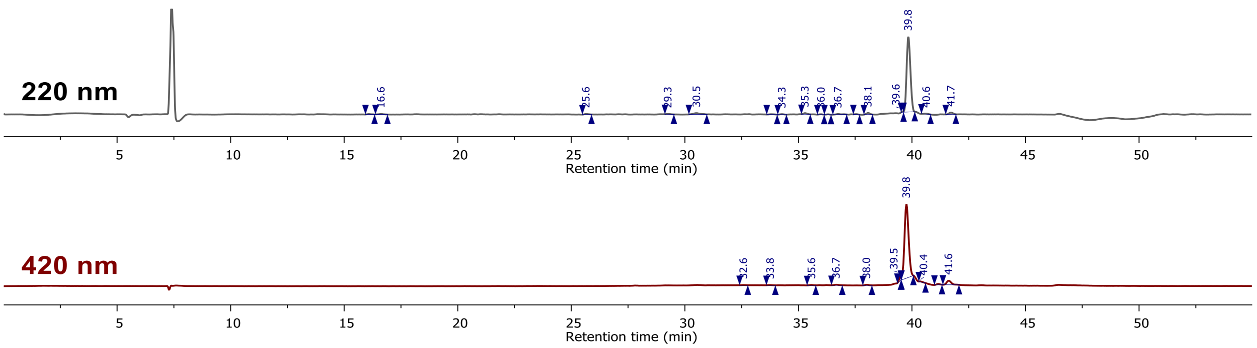

Figure S3. The HPLC chromogram of crude post-cleavage mixture of *Entry 3* on **Figure 1B** based on the absorbance at 220 nm (grey, peptide bond) and 420 nm (red, corrole/porphyrins). The composition (absorbance at 220 nm) of crude post-cleavage mixture was presented as the following table:

Peak No.	t_R (min)	Area	Area (%)	Proposed product
1	41.722	1.359	2.11	
2	40.640	0.411	0.64	
3	39.838	55.633	86.56	Desired porphyrin-peptide conjugate (**4a**)
4	39.611	0.271	0.42	
5	38.068	1.063	1.65	
6	37.560	0.200	0.31	
7	36.745	0.555	0.86	
8	36.311	0.178	0.28	
9	36.004	0.219	0.34	
10	35.295	0.772	1.20	
11	34.280	0.296	0.46	
12	33.826	0.319	0.50	
13	30.519	1.605	2.50	
14	29.256	0.246	0.38	
15	25.649	0.443	0.69	
16	16.624	0.405	0.63	
17	16.076	0.295	0.46	

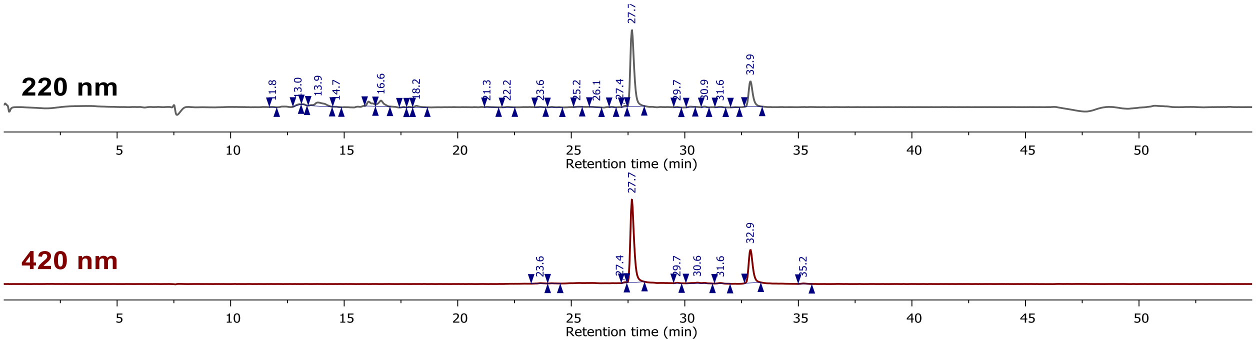

Figure S4. The HPLC chromogram of crude post-cleavage mixture of *Entry 4* on **Figure 1B** based on the absorbance at 220 nm (grey, peptide bond) and 420 nm (red, corrole/porphyrins). The composition (absorbance at 220 nm) of crude post-cleavage mixture was presented as the following table:

Peak No.	t_R (min)	Area	Area (%)	Proposed product
1	32.887	47.964	20.23	Desired porphyrin-peptide conjugate (**4b**)
2	32.207	0.353	0.15	
3	31.561	0.924	0.39	
4	30.867	1.028	0.43	
5	30.207	0.999	0.42	
6	29.661	0.582	0.25	
7	27.668	131.622	55.52	Corrole-peptide conjugate (**3**)
8	27.368	1.510	0.64	
9	26.794	0.397	0.17	
10	26.121	0.492	0.21	
11	25.248	0.436	0.18	
12	24.148	1.063	0.45	
13	23.642	0.587	0.25	
14	22.182	0.852	0.36	
15	21.288	0.652	0.28	
16	18.182	3.035	1.28	
17	17.849	0.690	0.29	
18	17.622	0.676	0.29	
19	16.622	13.929	5.88	
20	16.082	10.258	4.33	
21	14.656	0.355	0.15	
22	13.856	16.447	6.94	
23	13.223	0.529	0.22	
24	12.969	1.315	0.55	
25	11.850	0.385	0.16	

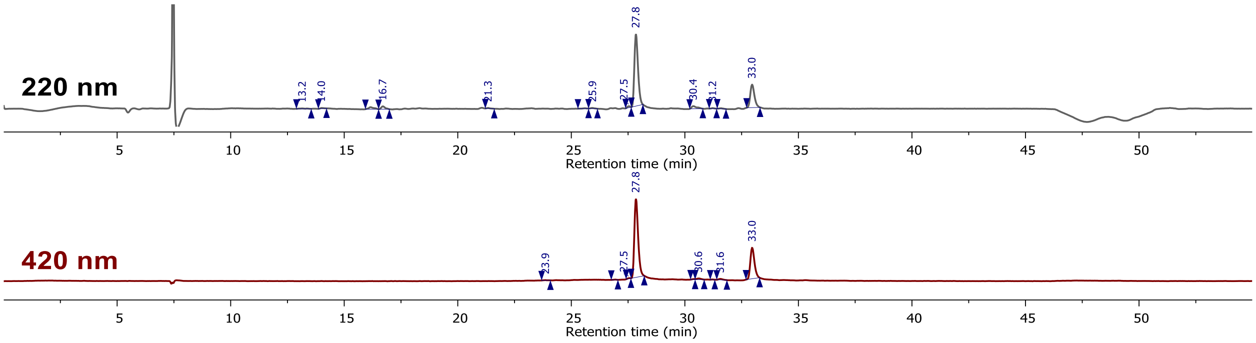

Figure S5. The HPLC chromogram of crude post-cleavage mixture of *Entry 5* on **Figure 1B** based on the absorbance at 220 nm (grey, peptide bond) and 420 nm (red, corrole/porphyrins). The composition (absorbance at 220 nm) of crude post-cleavage mixture was presented as the following table:

Peak No.	t_R (min)	Area	Area (%)	Proposed product
1	32.959	24.225	23.27	Desired porphyrin-peptide conjugate (**4b**)
2	31.599	0.552	0.53	
3	31.226	0.378	0.36	
4	30.372	3.538	3.40	
5	27.846	65.959	63.36	Corrole-peptide conjugate (**3**)
6	27.526	1.011	0.97	
7	25.940	0.680	0.65	
8	25.586	0.415	0.40	
9	21.340	0.540	0.52	
10	16.701	2.774	2.66	
11	16.161	2.684	2.58	
12	14.001	0.591	0.57	
13	13.154	0.753	0.72	

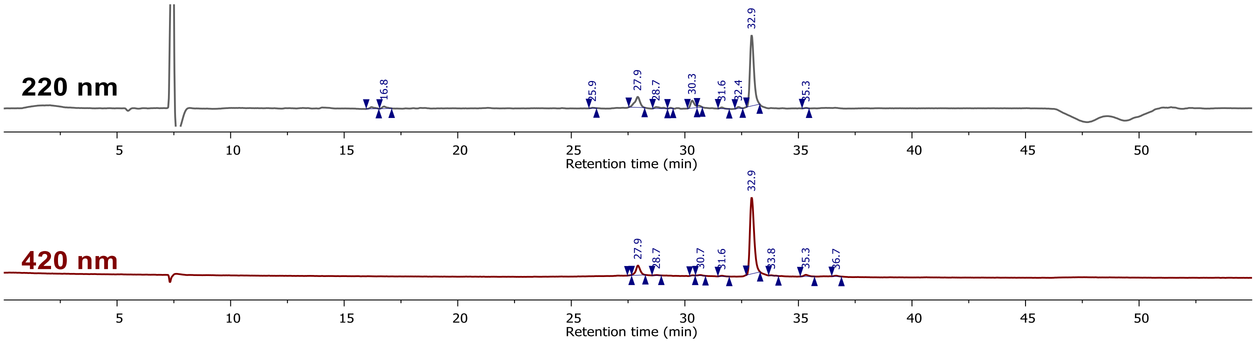

Figure S6. The HPLC chromogram of crude post-cleavage mixture of *Entry 6* on **Figure 1B** based on the absorbance at 220 nm (grey, peptide bond) and 420 nm (red, corrole/porphyrins). The composition (absorbance at 220 nm) of crude post-cleavage mixture was presented as the following table:

Peak No.	t_R (min)	Area	Area (%)	Proposed product
1	35.335	0.345	0.40	
2	32.942	57.986	67.76	Desired porphyrin-peptide conjugate (**4b**)
3	32.362	0.971	1.13	
4	31.629	0.939	1.10	
5	30.616	1.224	1.43	
6	30.322	6.037	7.06	
7	29.376	0.402	0.47	
8	28.736	2.153	2.52	
9	27.923	11.438	13.37	Corrole-peptide conjugate (**3**)
10	25.930	0.324	0.38	
11	16.757	2.047	2.39	
12	16.204	1.707	1.99	

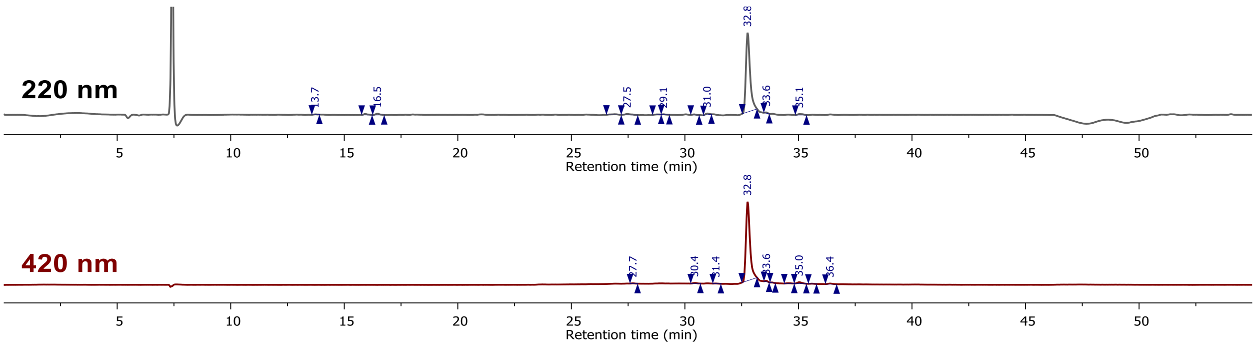

Figure S7. The HPLC chromogram of crude post-cleavage mixture of *Entry 7* on **Figure 1B** based on the absorbance at 220 nm (grey, peptide bond) and 420 nm (red, corrole/porphyrins). The composition (absorbance at 220 nm) of crude post-cleavage mixture was presented as the following table:

Peak No.	t_R (min)	Area	Area (%)	Proposed product
1	35.050	2.183	1.50	
2	33.597	0.813	0.56	
3	32.764	129.380	89.20	Desired porphyrin-peptide conjugate (**4b**)
4	30.984	1.792	1.24	
5	30.397	0.769	0.53	
6	29.064	0.444	0.31	
7	28.771	0.649	0.45	
8	27.464	2.630	1.81	Corrole-peptide conjugate (**3**)
9	26.904	1.936	1.33	
10	16.466	2.184	1.51	
11	15.912	1.796	1.24	
12	13.719	0.478	0.33	

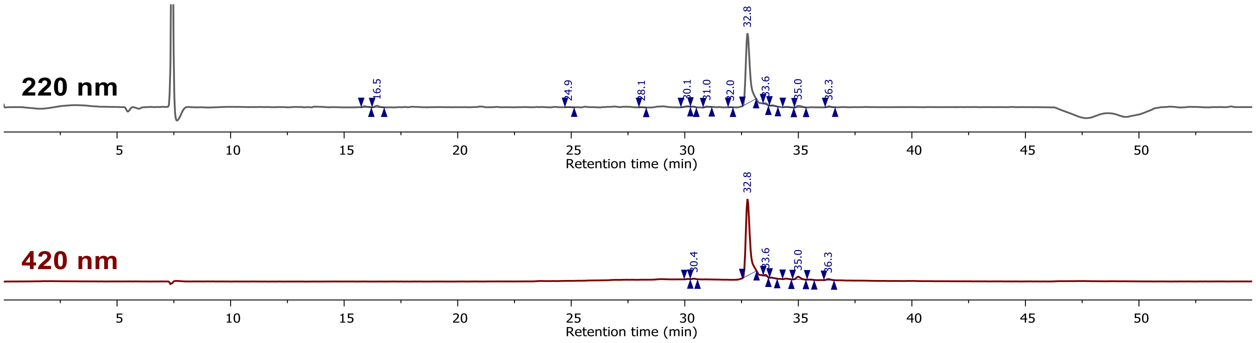

Figure S8. The HPLC chromogram of crude post-cleavage mixture of *Entry 8* on **Figure 1B** based on the absorbance at 220 nm (grey, peptide bond) and 420 nm (red, corrole/porphyrins). The composition (absorbance at 220 nm) of crude post-cleavage mixture was presented as the following table:

**Peak No.**	**t_R (min)**	**Area**	**Area (%)**	**Proposed product**
1	36.348	1.115	1.07	
2	34.995	2.674	2.57	
3	34.449	0.835	0.80	
4	33.869	0.938	0.90	
5	33.569	0.988	0.95	
6	32.762	90.852	87.27	Desired porphyrin-peptide conjugate (**4b**)
7	32.015	0.135	0.13	
8	30.976	0.889	0.85	
9	30.402	0.472	0.45	
10	30.109	0.953	0.92	
11	28.096	0.328	0.32	
12	24.876	0.260	0.25	

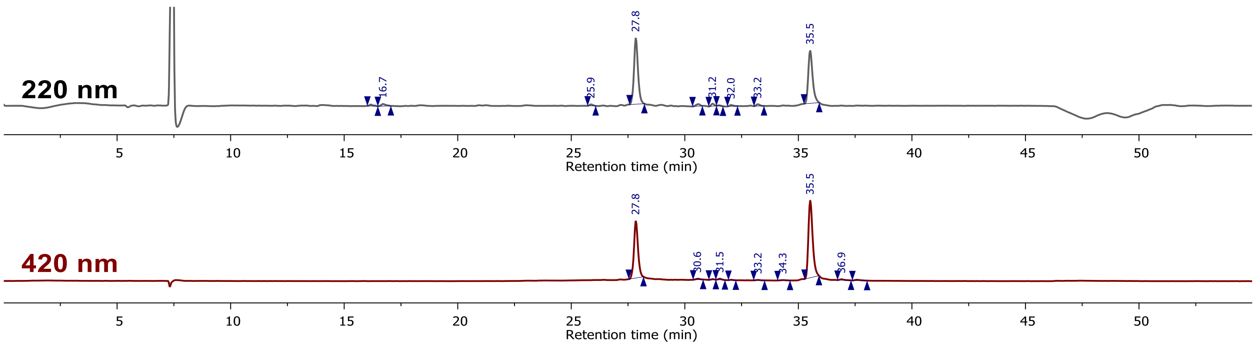

Figure S9. The HPLC chromogram of crude post-cleavage mixture of *Entry 9* on **Figure 1B** based on the absorbance at 220 nm (grey, peptide bond) and 420 nm (red, corrole/porphyrins). The composition (absorbance at 220 nm) of crude post-cleavage mixture was presented as the following table:

Peak No.	t_R (min)	Area	Area (%)	Proposed product
1	35.520	55.357	42.15	Desired porphyrin-peptide conjugate (**4c**)
2	33.214	1.602	1.22	
3	32.040	1.089	0.83	
4	31.514	0.505	0.38	
5	31.221	1.709	1.30	
6	30.587	2.004	1.53	
7	27.841	63.895	48.66	Corrole-peptide conjugate (**3**)
8	25.868	1.201	0.91	
9	16.709	2.505	1.91	
10	16.176	1.455	1.11	

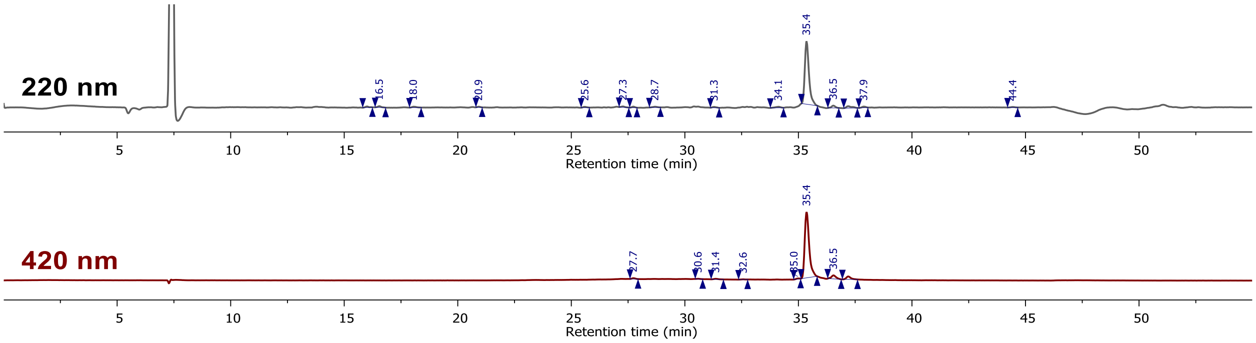

Figure S10. The HPLC chromogram of crude post-cleavage mixture of *Entry 10* on **Figure 1B** based on the absorbance at 220 nm (grey, peptide bond) and 420 nm (red, corrole/porphyrins). The composition (absorbance at 220 nm) of crude post-cleavage mixture was presented as the following table:

**Peak No.**	**t_R (min)**	**Area**	**Area (%)**	**Proposed product**
1	44.444	0.772	0.70	
2	37.878	1.484	1.35	
3	37.198	3.590	3.26	
4	36.538	2.981	2.71	
5	35.358	89.485	81.19	Desired porphyrin-peptide conjugate (**4c**)
6	34.119	1.892	1.72	
7	31.292	1.229	1.12	
8	28.679	0.960	0.87	
9	27.733	1.087	0.99	Corrole-peptide conjugate (**3**)
10	27.279	0.967	0.88	
11	25.600	1.041	0.94	
12	20.920	0.607	0.55	
13	18.047	1.357	1.23	
14	16.547	1.522	1.38	
15	16.007	1.241	1.13	

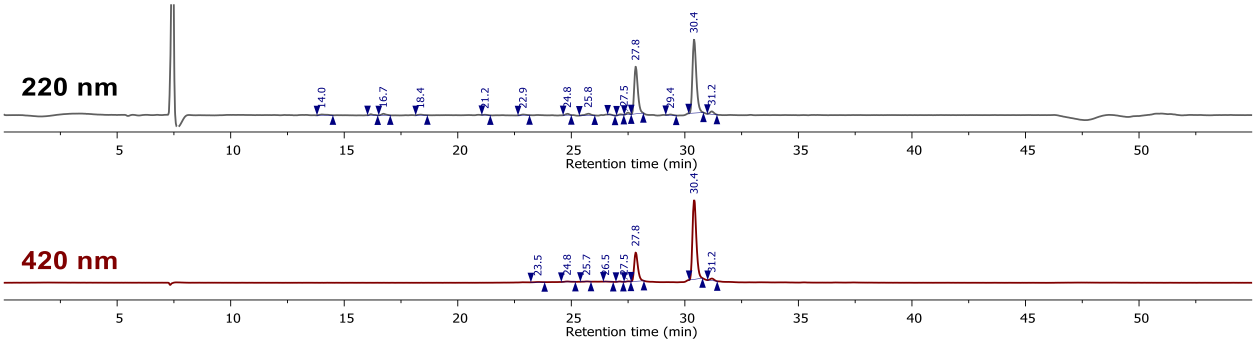

Figure S11. The HPLC chromogram of crude post-cleavage mixture of *Entry 11* on **Figure 1B** based on the absorbance at 220 nm (grey, peptide bond) and 420 nm (red, corrole/porphyrins). The composition (absorbance at 220 nm) of crude post-cleavage mixture was presented as the following table:

Peak No.	t_R (min)	Area	Area (%)	Proposed product
1	31.192	4.314	2.08	
2	30.406	114.068	54.93	Desired porphyrin-peptide conjugate (**4d**)
3	29.372	1.258	0.61	
4	27.839	68.284	32.88	Corrole-peptide conjugate (**3**)
5	27.493	2.216	1.07	
6	27.153	1.109	0.53	
7	26.706	0.555	0.27	
8	25.753	3.932	1.89	
9	24.833	1.931	0.93	
10	22.880	1.461	0.70	
11	21.207	0.594	0.29	
12	18.367	1.265	0.61	
13	16.727	2.618	1.26	
14	16.167	1.644	0.79	
15	14.014	2.424	1.17	

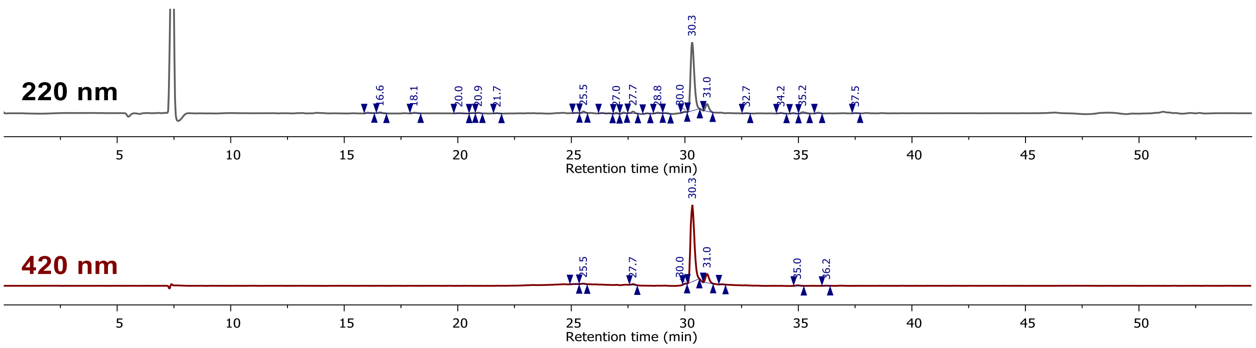

Figure S12. The HPLC chromogram of crude post-cleavage mixture of *Entry 12* on **Figure 1B** based on the absorbance at 220 nm (grey, peptide bond) and 420 nm (red, corrole/porphyrins). The composition (absorbance at 220 nm) of crude post-cleavage mixture was presented as the following table:

**Peak No.**	t_R **(min)**	**Area**	**Area (%)**	**Proposed product**
1	37.510	0.839	0.44	
2	35.857	1.468	0.76	
3	35.183	3.750	1.95	
4	34.750	1.255	0.65	
5	34.230	1.323	0.69	
6	32.710	0.697	0.36	
7	30.977	14.162	7.36	
8	30.317	144.342	74.96	Desired porphyrin-peptide conjugate (**4d**)
9	30.004	1.288	0.67	
10	29.177	0.997	0.52	
11	28.804	1.435	0.75	
12	28.344	0.986	0.51	
13	27.718	3.511	1.82	Corrole-peptide conjugate (**3**)
14	27.284	1.830	0.95	
15	26.984	1.520	0.79	
16	26.358	1.674	0.87	
17	25.538	2.608	1.35	
18	25.211	0.673	0.35	
19	21.732	0.718	0.37	
20	20.905	0.997	0.52	
21	20.639	0.652	0.34	
22	20.045	1.317	0.68	
23	18.072	1.527	0.79	
24	16.579	1.673	0.87	
25	16.032	1.303	0.68	

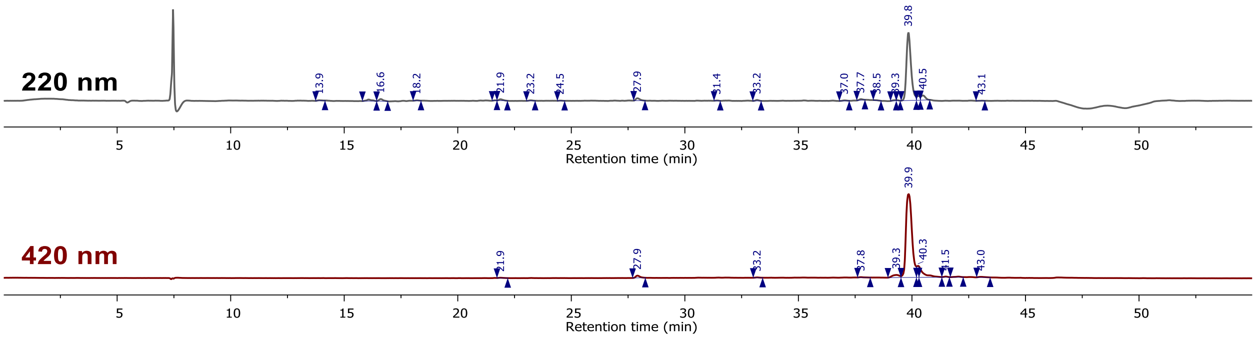

Figure S13. The HPLC chromogram of crude post-cleavage mixture of *Entry 13* on **Figure 1B** based on the absorbance at 220 nm (grey, peptide bond) and 420 nm (red, corrole/porphyrins). The composition (absorbance at 220 nm) of crude post-cleavage mixture was presented as the following table:

Peak No.	t_R (min)	Area	Area (%)	Proposed product
1	43.077	0.312	0.19	
2	40.484	8.371	5.11	
3	40.278	5.571	3.40	
4	39.845	128.112	78.18	Desired porphyrin-peptide conjugate (**4e**)
5	39.311	0.546	0.33	
6	39.278	1.021	0.62	
7	38.458	0.307	0.19	
8	37.745	1.388	0.85	
9	37.025	0.818	0.50	
10	33.179	1.243	0.76	
11	31.412	0.386	0.24	
12	27.913	3.979	2.43	Corrole-peptide conjugate (**3**)
13	24.513	0.313	0.19	
14	23.213	0.730	0.45	
15	21.887	2.280	1.39	
16	21.633	0.302	0.18	
17	18.200	0.568	0.35	
18	16.607	3.488	2.13	
19	16.074	3.294	2.01	
20	13.914	0.845	0.52	

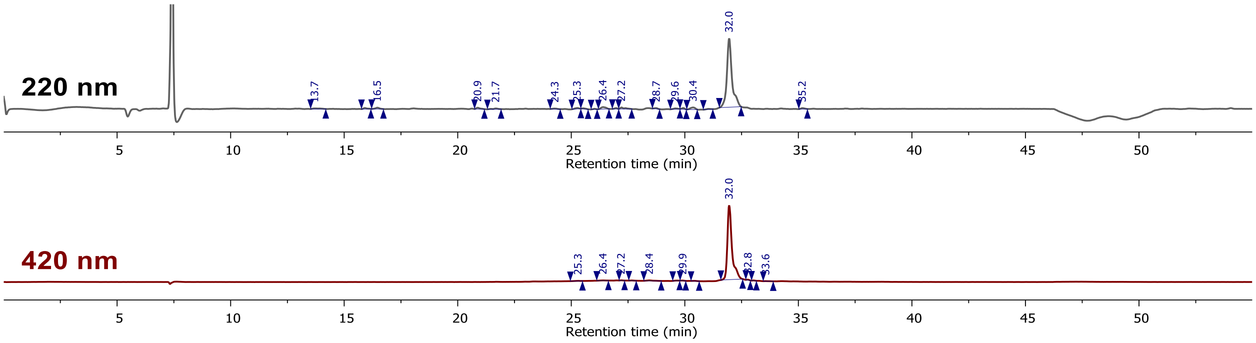

Figure S14. The HPLC chromogram of crude post-cleavage mixture of *Entry 14* on **Figure 1B** based on the absorbance at 220 nm (grey, peptide bond) and 420 nm (red, corrole/porphyrins). The composition (absorbance at 220 nm) of crude post-cleavage mixture was presented as the following table:

Peak No.	t_R (min)	Area	Area (%)	Proposed product
1	35.173	1.119	0.82	
2	31.954	112.558	82.27	Desired porphyrin-peptide conjugate (**4f**)
3	31.027	0.674	0.49	
4	30.367	3.128	2.29	
5	29.934	1.045	0.76	
6	29.574	0.892	0.65	
7	28.748	0.810	0.59	
8	27.214	2.280	1.67	Corrole-peptide conjugate (**3**)
9	26.968	0.755	0.55	
10	26.395	3.176	2.32	
11	26.015	0.422	0.31	
12	25.528	0.721	0.53	
13	25.261	1.197	0.87	
14	24.281	0.739	0.54	
15	21.675	1.137	0.83	
16	20.882	1.632	1.19	
17	16.462	1.743	1.27	
18	15.909	1.213	0.89	
19	13.703	1.576	1.15	

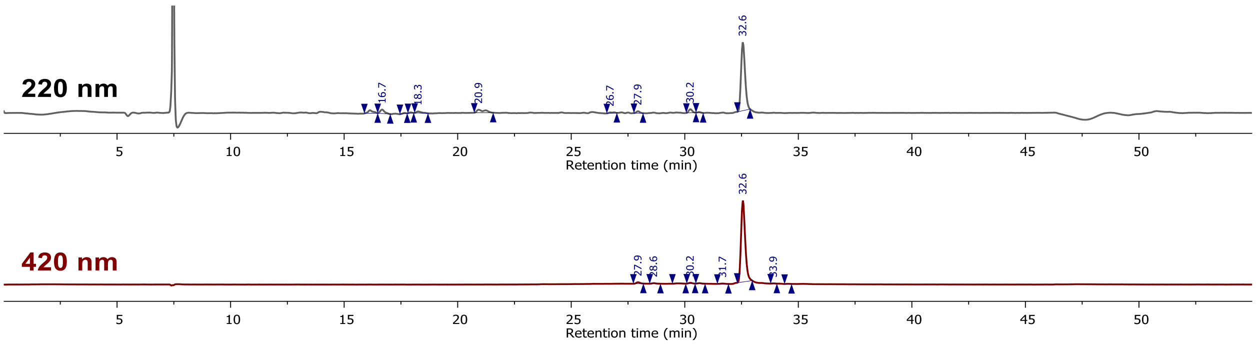

Figure S15. The HPLC chromogram of crude post-cleavage mixture of *Entry 15* on **Figure 1B** based on the absorbance at 220 nm (grey, peptide bond) and 420 nm (red, corrole/porphyrins). The composition (absorbance at 220 nm) of crude post-cleavage mixture was presented as the following table:

Peak No.	t_R (min)	Area	Area (%)	Proposed product
1	32.555	82.209	74.52	Desired porphyrin-peptide conjugate (**4g**)
2	30.629	0.712	0.65	
3	30.242	3.681	3.34	
4	27.936	1.876	1.70	Corrole-peptide conjugate (**3**)
5	26.716	1.228	1.11	
6	20.923	7.516	6.81	
7	18.257	2.259	2.05	
8	17.911	0.508	0.46	
9	17.637	0.651	0.59	
10	16.677	5.131	4.65	
11	16.137	4.545	4.12	

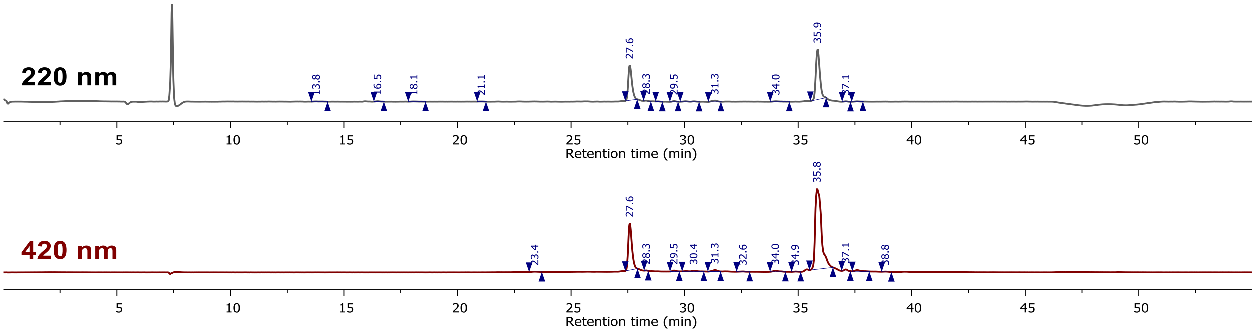

Figure S16. The HPLC chromogram of crude post-cleavage mixture of *Entry 16* on **Figure 1B** based on the absorbance at 220 nm (grey, peptide bond) and 420 nm (red, corrole/porphyrins). The composition (absorbance at 220 nm) of crude post-cleavage mixture was presented as the following table:

Peak No.	t_R (min)	Area	Area (%)	Proposed product
1	37.591	1.328	0.41	
2	37.098	1.210	0.37	
3	35.858	185.101	56.61	Desired porphyrin-peptide conjugate (**4h**)
4	34.025	2.617	0.80	
5	31.339	6.012	1.84	
6	30.046	3.517	1.08	
7	29.539	1.842	0.56	
8	28.853	0.368	0.11	
9	28.319	1.022	0.31	
10	27.586	118.910	36.37	Corrole-peptide conjugate (**3**)
11	21.060	0.626	0.19	
12	18.081	1.363	0.42	
13	16.487	1.241	0.38	
14	13.781	1.798	0.55	

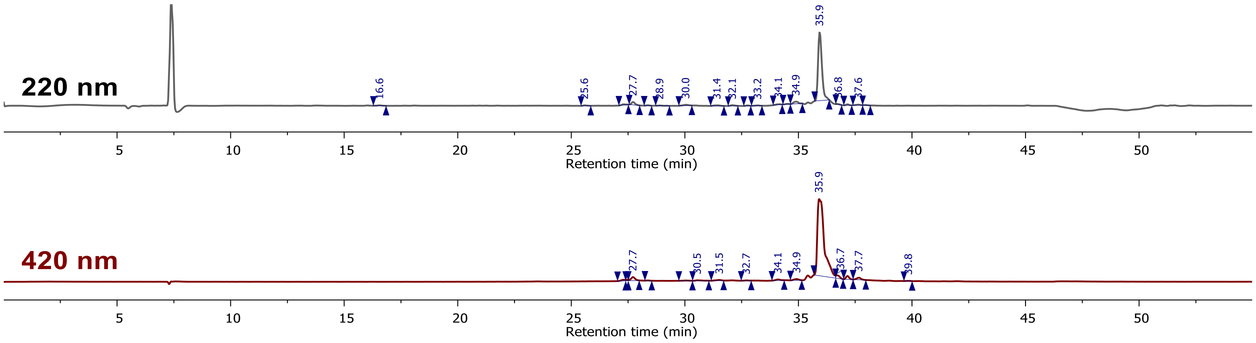

Figure S17. The HPLC chromogram of crude post-cleavage mixture of *Entry 17* on **Figure 1B** based on the absorbance at 220 nm (grey, peptide bond) and 420 nm (red, corrole/porphyrins). The composition (absorbance at 220 nm) of crude post-cleavage mixture was presented as the following table:

Peak No.	t_R (min)	Area	Area (%)	Proposed product
1	37.961	0.749	0.32	
2	37.648	1.630	0.69	
3	37.162	2.100	0.89	
4	36.762	0.808	0.34	
5	35.935	195.905	82.86	Desired porphyrin-peptide conjugate (**4h**)
6	34.895	7.662	3.24	
7	34.495	0.901	0.38	
8	34.122	1.752	0.74	
9	33.209	1.125	0.48	
10	32.749	0.976	0.41	
11	32.095	2.143	0.91	
12	31.416	2.820	1.19	
13	30.042	3.360	1.42	
14	28.896	1.058	0.45	
15	28.383	0.984	0.42	
16	27.723	7.435	3.14	Corrole-peptide conjugate (**3**)
17	27.283	2.268	0.96	
18	25.583	1.384	0.59	
19	16.564	1.360	0.58	

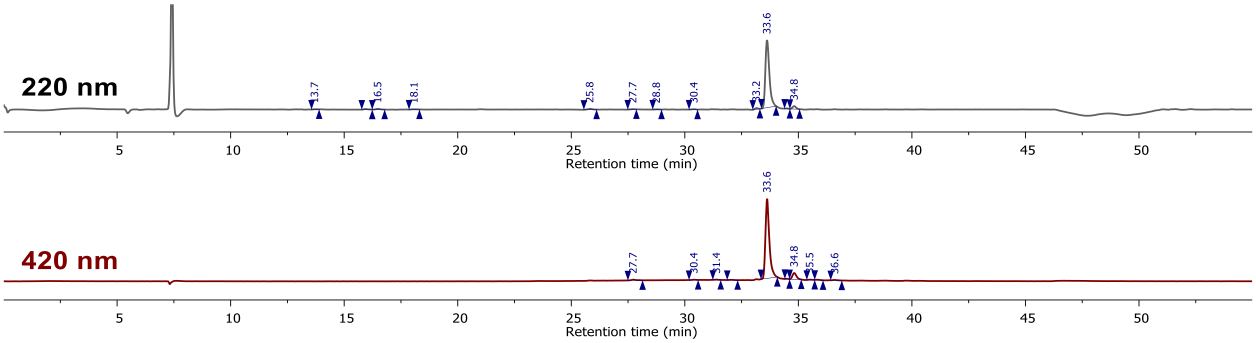

Figure S18. The HPLC chromogram of crude post-cleavage mixture of *Entry 18* on **Figure 1B** based on the absorbance at 220 nm (grey, peptide bond) and 420 nm (red, corrole/porphyrins). The composition (absorbance at 220 nm) of crude post-cleavage mixture was presented as the following table:

**Peak No.**	**t_R (min)**	**Area**	**Area (%)**	**Proposed product**
1	34.820	6.648	3.50	
2	34.507	0.525	0.28	
3	33.620	170.780	89.90	Desired porphyrin-peptide conjugate (**4i**)
4	33.154	2.214	1.17	
5	30.407	0.929	0.49	
6	28.754	0.599	0.32	
7	27.714	0.734	0.39	
8	25.821	2.370	1.25	
9	18.082	0.805	0.42	
10	16.482	2.171	1.14	
11	15.942	1.639	0.86	
12	13.703	0.542	0.29	

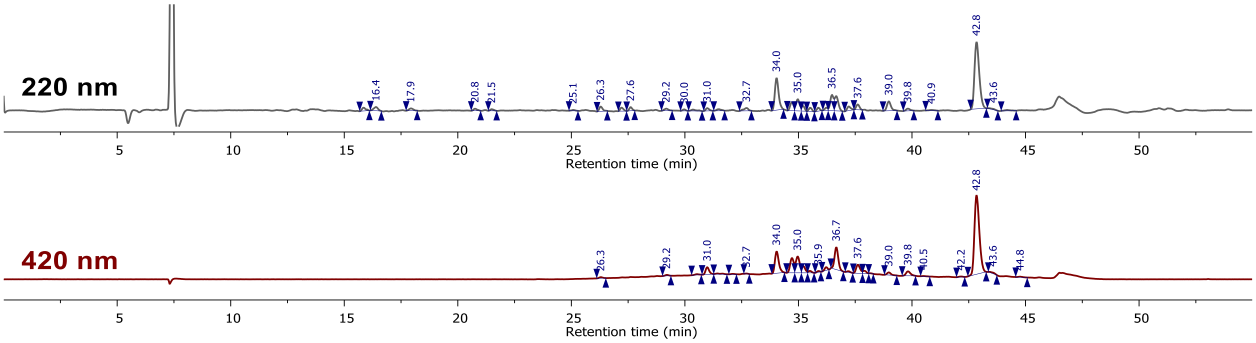

Figure S19. The HPLC chromogram of crude post-cleavage mixture of *Entry 19* on **Figure 1B** based on the absorbance at 220 nm (grey, peptide bond) and 420 nm (red, corrole/porphyrins). The composition (absorbance at 220 nm) of crude post-cleavage mixture was presented as the following table:

Peak No.	t_R (min)	Area	Area (%)	Proposed product
1	44.196	1.294	0.65	
2	43.596	0.985	0.49	
3	42.843	70.345	35.19	Desired porphyrin-peptide conjugate (**4j**)
4	40.863	0.766	0.38	
5	39.816	2.036	1.02	
6	38.983	8.401	4.20	
7	37.616	3.918	1.96	
8	37.210	3.273	1.64	
9	36.663	12.031	6.02	
10	36.477	12.054	6.03	
11	36.230	1.745	0.87	
12	35.870	2.198	1.10	
13	35.517	2.538	1.27	
14	35.203	3.328	1.66	
15	34.970	9.738	4.87	
16	34.710	6.513	3.26	
17	34.037	26.835	13.42	
18	32.717	3.018	1.51	
19	31.504	1.768	0.88	
20	30.984	2.090	1.05	
21	30.351	1.631	0.82	
22	29.977	1.038	0.52	
23	29.177	1.823	0.91	
24	27.611	2.350	1.18	
25	27.211	2.522	1.26	
26	26.291	3.512	1.76	
27	25.078	0.758	0.38	
28	21.492	1.264	0.63	
29	20.759	1.675	0.84	
30	17.932	2.160	1.08	
31	16.399	3.518	1.76	
32	15.846	2.783	1.39	

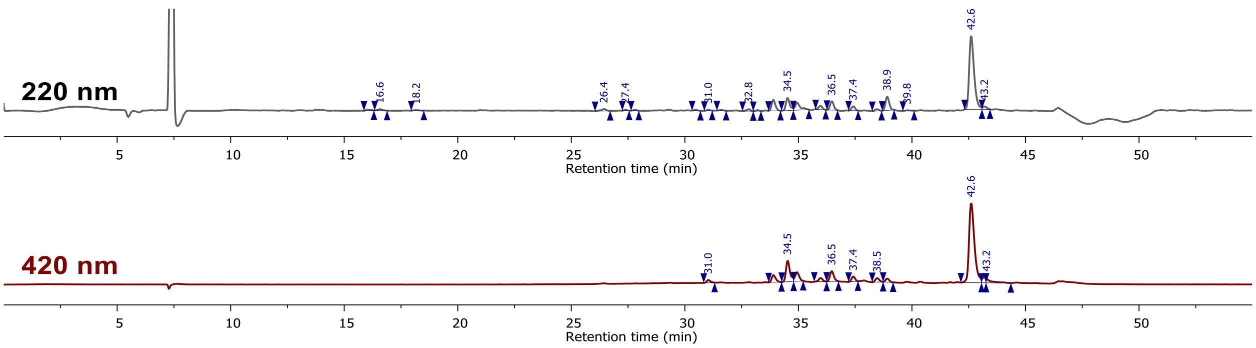

Figure S20. The HPLC chromogram of crude post-cleavage mixture of *Entry 20* on **Figure 1B** based on the absorbance at 220 nm (grey, peptide bond) and 420 nm (red, corrole/porphyrins). The composition (absorbance at 220 nm) of crude post-cleavage mixture was presented as the following table:

Peak No.	t_R (min)	Area	Area (%)	Proposed product
1	43.197	3.598	1.77	
2	42.611	109.130	53.66	Desired porphyrin-peptide conjugate (**4j**)
3	39.791	0.777	0.38	
4	38.911	15.647	7.69	
5	38.451	2.078	1.02	
6	37.411	4.606	2.26	
7	36.472	9.750	4.79	
8	35.978	4.222	2.08	
9	34.945	11.369	5.59	
10	34.525	14.977	7.36	
11	33.899	12.549	6.17	
12	33.199	0.855	0.42	
13	32.812	2.266	1.11	
14	31.606	0.857	0.42	
15	31.039	0.994	0.49	
16	30.472	0.895	0.44	
17	27.793	0.773	0.38	
18	27.386	0.662	0.33	
19	26.439	3.200	1.57	
20	18.180	0.980	0.48	
21	16.581	1.877	0.92	
22	16.021	1.320	0.65	

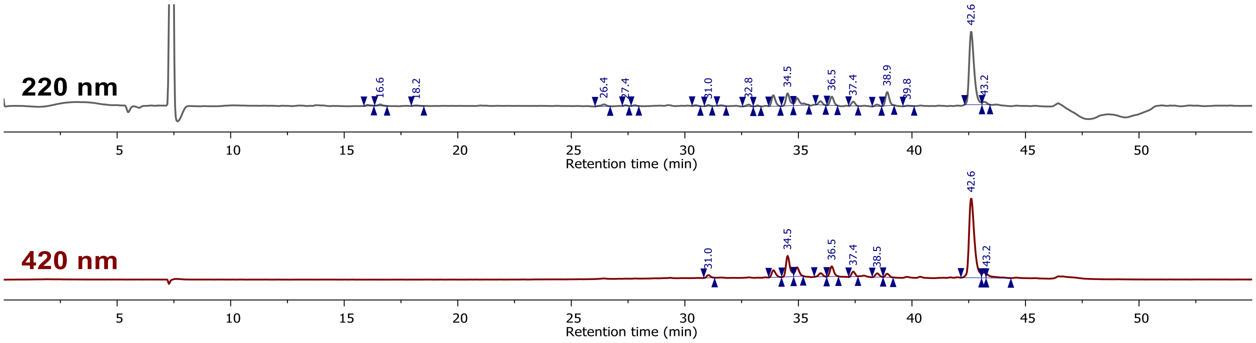

Figure S21. The HPLC chromogram of crude post-cleavage mixture of *Entry 21* on **Figure 1B** based on the absorbance at 220 nm (grey, peptide bond) and 420 nm (red, corrole/porphyrins). The composition (absorbance at 220 nm) of crude post-cleavage mixture was presented as the following table:

Peak No.	t_R (min)	Area	Area (%)	Proposed product
1	43.769	5.369	2.04	
2	43.076	188.211	71.36	Desired porphyrin-peptide conjugate (**4j**)
3	38.990	13.512	5.12	
4	38.243	0.853	0.32	
5	37.143	1.626	0.62	
6	36.437	2.693	1.02	
7	35.677	6.894	2.61	
8	35.190	6.582	2.50	
9	34.803	4.164	1.58	
10	32.290	3.712	1.41	
11	29.817	0.924	0.35	
12	29.044	1.126	0.43	
13	26.958	5.611	2.13	
14	26.644	0.814	0.31	
15	26.305	0.784	0.30	
16	25.831	4.572	1.73	
17	24.225	0.468	0.18	
18	21.098	0.469	0.18	
19	17.099	4.256	1.61	
20	15.779	1.458	0.55	
21	15.393	9.651	3.66	

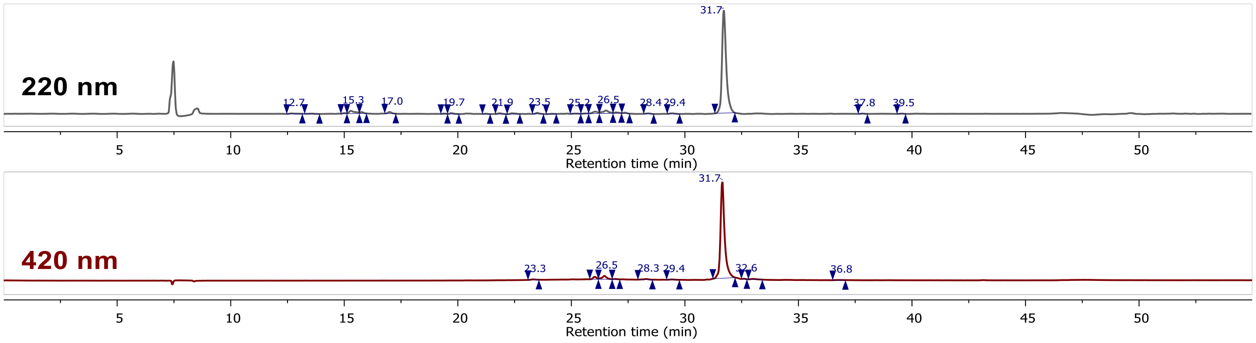

Figure S22. The HPLC chromogram of crude post-cleavage mixture of *Entry 22* on **Figure 1B** based on the absorbance at 220 nm (grey, peptide bond) and 420 nm (red, corrole/porphyrins). The composition (absorbance at 220 nm) of crude post-cleavage mixture was presented as the following table:

**Peak No.**	**t_R (min)**	**Area**	**Area (%)**	**Proposed product**
1	39.529	0.174	0.21	
2	37.799	0.195	0.24	
3	31.713	66.441	81.88	Desired porphyrin-peptide conjugate (**4k**)
4	29.442	0.480	0.59	
5	28.393	0.373	0.46	
6	27.371	0.356	0.44	
7	26.963	0.542	0.67	
8	26.529	2.731	3.37	
9	26.061	1.554	1.92	
10	25.641	0.184	0.23	
11	25.246	0.226	0.28	
12	24.144	0.165	0.20	
13	23.496	0.583	0.72	
14	22.387	0.683	0.84	
15	21.859	0.502	0.62	
16	21.231	0.162	0.20	
17	19.722	0.482	0.59	
18	19.401	0.171	0.21	
19	17.003	0.896	1.10	
20	15.774	0.720	0.89	
21	15.293	2.551	3.14	
22	15.025	0.312	0.38	
23	13.596	0.259	0.32	
24	12.674	0.408	0.50	

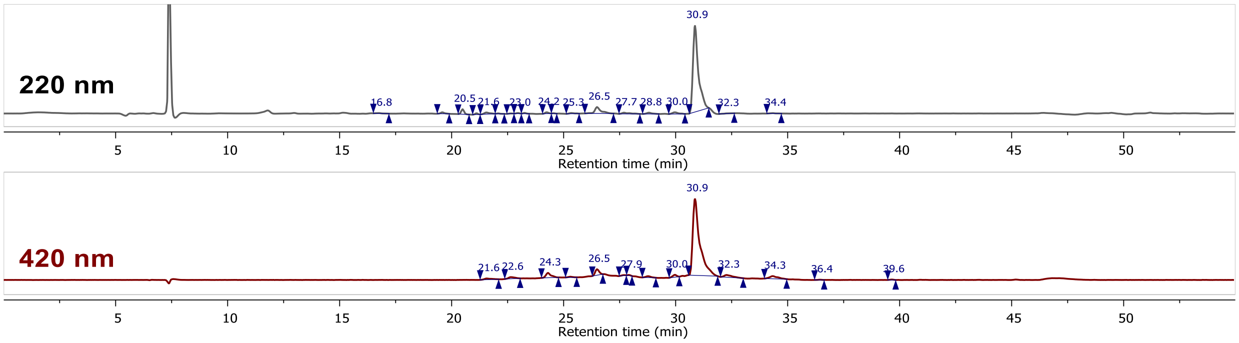

Figure S23. The HPLC chromogram of crude post-cleavage mixture of *Entry 23* on **Figure 1B** based on the absorbance at 220 nm (grey, peptide bond) and 420 nm (red, corrole/porphyrins). The composition (absorbance at 220 nm) of crude post-cleavage mixture was presented as the following table:

Peak No.	t_R (min)	Area	Area (%)	Proposed product
1	34.357	0.947	0.35	
2	32.250	0.620	0.23	
3	30.864	217.216	79.83	Desired porphyrin-peptide conjugate (**4l**)
4	29.964	4.624	1.70	
5	28.804	3.105	1.14	
6	27.691	4.294	1.58	
7	26.491	18.075	6.64	Corrole-peptide conjugate (**3**)
8	25.338	1.686	0.62	
9	24.558	0.330	0.12	
10	24.238	2.201	0.81	
11	23.258	1.130	0.42	
12	22.952	0.959	0.35	
13	22.612	0.445	0.16	
14	22.078	0.544	0.20	
15	21.552	4.149	1.52	
16	21.105	1.568	0.58	
17	20.485	6.820	2.51	
18	19.585	2.559	0.94	
19	16.759	0.817	0.30	

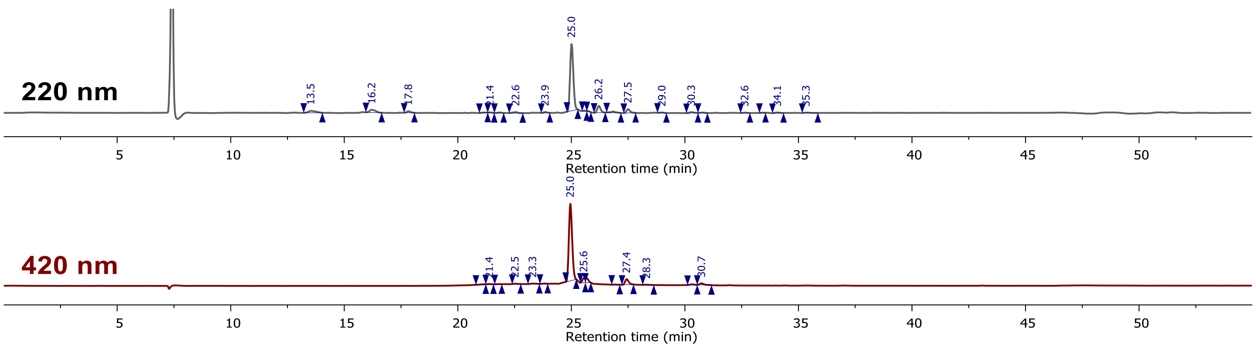

Figure S24. The HPLC chromogram of crude post-cleavage mixture of stepwise peptide ligation with 3 equiv. aldehyde-functionalized RGD peptide (row 2, **Figure 3G**) based on the absorbance at 220 nm (grey, peptide bond) and 420 nm (red, corrole/porphyrins). The composition (absorbance at 220 nm) of crude post-cleavage mixture was presented as the following table:

Peak No.	t_R (min)	Area	Area (%)	Proposed product
1	35.341	0.700	0.66	
2	34.078	0.882	0.84	
3	33.437	0.290	0.27	
4	32.642	0.408	0.39	
5	30.798	0.640	0.61	
6	30.290	1.304	1.23	
7	28.994	0.486	0.46	
8	27.498	4.465	4.23	
9	26.850	1.963	1.86	
10	26.208	7.299	6.91	
11	25.754	0.315	0.30	
12	25.587	0.320	0.30	
13	25.013	70.723	66.96	Desired porphyrin-peptide conjugate (**4m**)
14	23.850	1.121	1.06	
15	22.554	1.538	1.46	
16	21.859	0.638	0.60	
17	21.425	0.364	0.35	
18	21.171	0.420	0.40	
19	17.831	1.834	1.74	
20	16.214	5.120	4.85	
21	13.529	4.782	4.53	

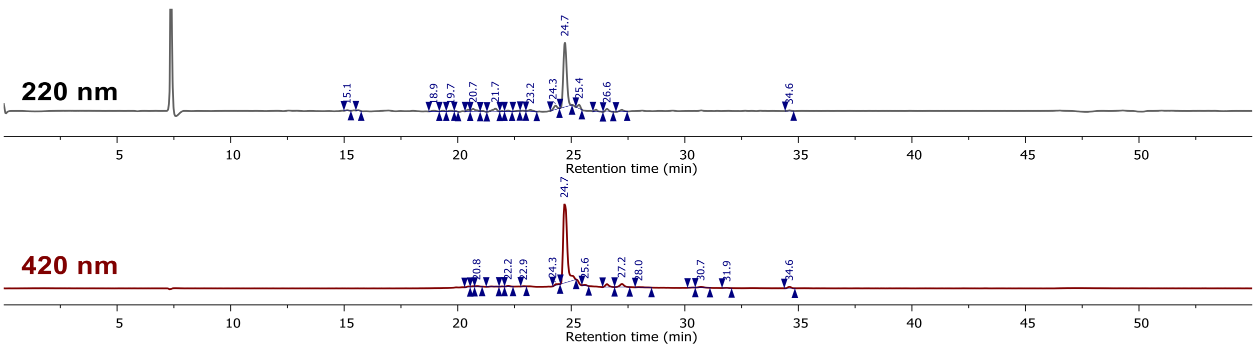

Figure S25. The HPLC chromogram of crude post-cleavage mixture of “direct-mix” peptide ligation with 3 equiv. aldehyde-functionalized RGD peptide (row 3, **Figure 3G**) based on the absorbance at 220 nm (grey, peptide bond) and 420 nm (red, corrole/porphyrins). The composition (absorbance at 220 nm) of crude post-cleavage mixture was presented as the following table:

Peak No.	t_R (min)	Area	Area (%)	Proposed product
1	34.607	1.463	0.71	
2	27.221	4.004	1.96	
3	26.574	6.023	2.94	
4	26.088	3.392	1.66	
5	25.355	6.181	3.02	
6	24.721	138.640	67.76	Desired porphyrin-peptide conjugate (**4m**)
7	24.301	8.268	4.04	
8	23.208	3.661	1.79	
9	22.862	0.628	0.31	
10	22.655	0.565	0.28	
11	22.215	1.713	0.84	
12	21.948	0.621	0.30	
13	21.662	9.108	4.45	
14	21.108	1.794	0.88	
15	20.682	8.366	4.09	
16	20.462	3.234	1.58	
17	19.929	0.415	0.20	
18	19.702	1.449	0.71	
19	19.349	1.303	0.64	
20	18.949	1.883	0.92	
21	15.636	0.952	0.47	
22	15.129	0.955	0.47	

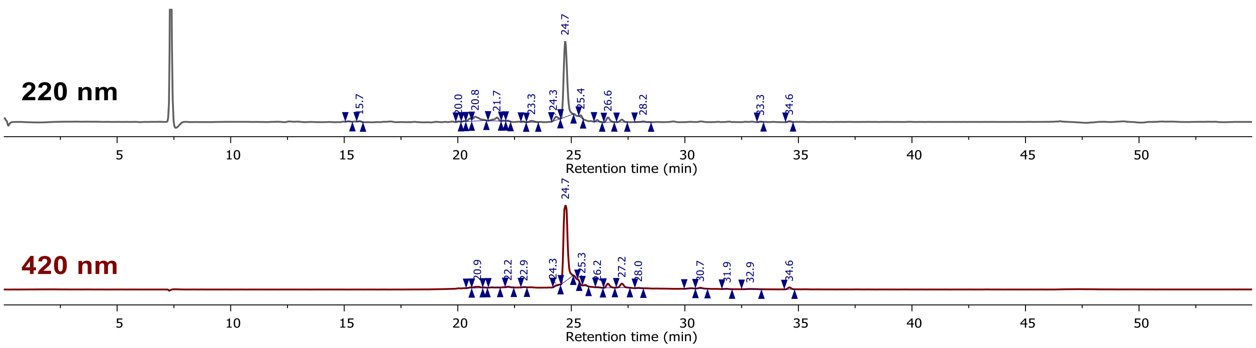

Figure S26. The HPLC chromogram of crude post-cleavage mixture of “direct-mix” peptide ligation with 2 equiv. aldehyde-functionalized RGD peptide (row 4, **Figure 3G**) based on the absorbance at 220 nm (grey, peptide bond) and 420 nm (red, corrole/porphyrins). The composition (absorbance at 220 nm) of crude post-cleavage mixture was presented as the following table:

**Peak No.**	**t_R (min)**	**Area**	**Area (%)**	**Proposed product**
1	34.605	1.572	0.69	
2	33.332	0.684	0.30	
3	28.173	1.992	0.87	
4	27.226	5.098	2.23	
5	26.619	9.510	4.17	
6	26.140	2.995	1.31	
7	25.426	2.661	1.17	
8	24.733	155.837	68.28	Desired porphyrin-peptide conjugate (**4m**)
9	24.320	7.132	3.12	
10	23.253	3.668	1.61	
11	22.893	0.894	0.39	
12	22.220	0.897	0.39	
13	22.013	0.586	0.26	
14	21.727	9.361	4.10	
15	20.774	16.012	7.02	
16	20.534	5.251	2.30	
17	20.307	1.024	0.45	
18	20.034	0.851	0.37	
19	15.681	1.287	0.56	
20	15.188	0.936	0.41	

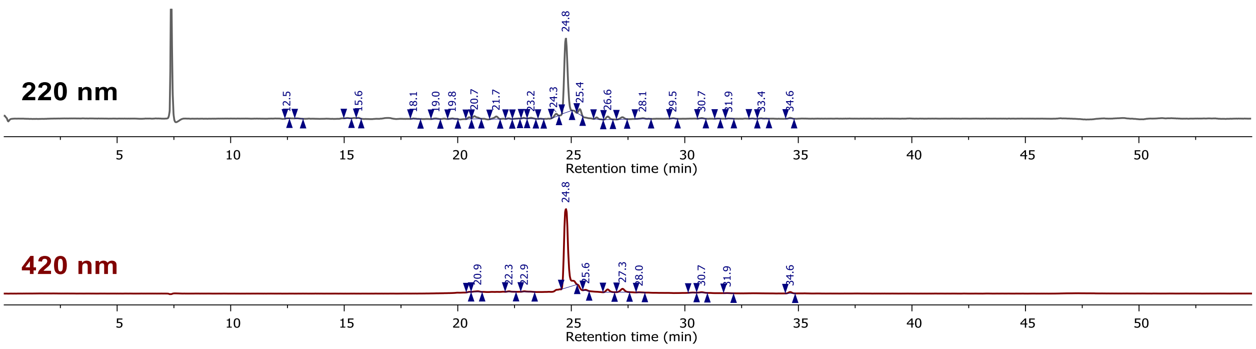

Figure S27. The HPLC chromogram of crude post-cleavage mixture of "direct-mix" peptide ligation with 1.25 equiv. aldehyde-functionalized RGD peptide (row 5, **Figure 3G**) based on the absorbance at 220 nm (grey, peptide bond) and 420 nm (red, corrole/porphyrins). The composition (absorbance at 220 nm) of crude post-cleavage mixture was presented as the following table:

Peak No.	t_R (min)	Area	Area (%)	Proposed product
1	34.636	1.557	0.84	
2	33.383	1.137	0.62	
3	33.003	0.530	0.29	
4	31.936	0.733	0.40	
5	31.449	0.347	0.19	
6	30.749	1.316	0.71	
7	29.476	0.985	0.53	
8	28.149	1.677	0.91	
9	27.249	4.035	2.18	
10	26.609	5.202	2.82	
11	26.123	2.529	1.37	
12	25.383	7.346	3.98	
13	24.763	128.369	69.48	Desired porphyrin-peptide conjugate (**4m**)
14	24.329	4.199	2.27	
15	23.663	0.390	0.21	
16	23.229	1.924	1.04	
17	22.903	1.033	0.56	
18	22.683	0.494	0.27	
19	22.229	0.993	0.54	
20	21.696	5.286	2.86	
21	20.736	6.187	3.35	
22	20.503	2.673	1.45	
23	19.763	1.231	0.67	
24	19.043	1.354	0.73	
25	18.056	1.045	0.57	
26	15.643	0.741	0.40	
27	15.149	0.642	0.35	
28	12.936	0.458	0.25	
29	12.476	0.348	0.19	

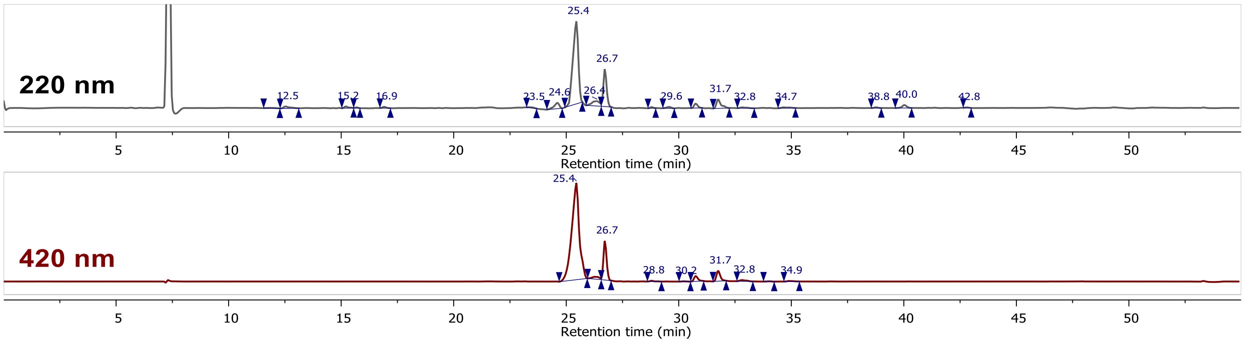

Figure S28. The HPLC chromogram of crude post-cleavage mixture of "direct-mix" peptide ligation with 1.5 equiv. aldehyde-functionalized XPKKKRKV peptide (row 6, **Figure 3G**) based on the absorbance at 220 nm (grey, peptide bond) and 420 nm (red, corrole/porphyrins). The composition (absorbance at 220 nm) of crude post-cleavage mixture was presented as the following table:

Peak No.	t_R (min)	Area	Area (%)	Proposed product
1	42.804	1.066	0.21	
2	40.018	8.949	1.80	
3	38.778	1.880	0.38	
4	34.665	4.111	0.82	
5	32.832	3.497	0.70	
6	31.746	26.658	5.35	
7	30.746	10.336	2.07	
8	29.572	4.128	0.83	
9	28.793	2.186	0.44	
10	26.713	79.349	15.92	Corrole-peptide conjugate (**3**)
11	26.366	24.640	4.94	
12	25.440	291.552	58.50	Desired porphyrin-peptide conjugate (**4n**)
13	24.600	19.341	3.88	
14	23.467	2.111	0.42	
15	16.914	2.842	0.57	
16	15.687	1.222	0.25	
17	15.208	4.573	0.92	
18	12.521	7.863	1.58	
19	11.921	2.104	0.42	

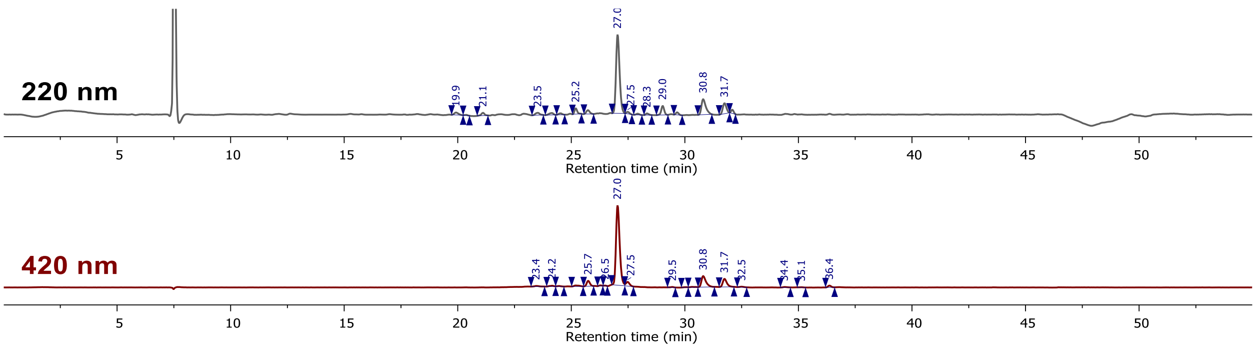

Figure S29. The HPLC chromogram of crude post-cleavage mixture of "direct-mix" peptide ligation with 2 equiv. aldehyde and biotin bifunctionalized KRGD peptide (row 7, **Figure 3G**) based on the absorbance at 220 nm (grey, peptide bond) and 420 nm (red, corrole/porphyrins). The composition (absorbance at 220 nm) of crude post-cleavage mixture was presented as the following table:

**Peak No.**	**t_R (min)**	**Area**	**Area (%)**	**Proposed product**
1	32.089	1.915	1.77	
2	31.742	7.865	7.28	
3	30.809	14.169	13.11	
4	29.662	1.488	1.38	
5	29.023	5.683	5.26	
6	28.336	1.040	0.96	
7	27.923	0.789	0.73	
8	27.483	1.221	1.13	
9	27.036	58.568	54.18	Desired porphyrin-peptide conjugate (**4o**)
10	25.730	2.761	2.55	
11	25.196	3.274	3.03	
12	24.530	1.067	0.99	
13	24.150	1.779	1.65	
14	23.523	2.105	1.95	
15	21.110	1.863	1.72	
16	20.390	0.338	0.31	
17	19.924	2.175	2.01	

References

(1) Eissler, S.; Kley, M.; Bächle, D.; Loidl, G.; Meier, T.; Samson, D. Substitution Determination of Fmoc-Substituted Resins at Different Wavelengths. *J. Pept. Sci.* **2017**, *23* (10), 757–762.

(2) Chau, H. F.; Wu, Y.; Fok, W. Y.; Thor, W.; Cho, W. C. S.; Ma, P.; Lin, J.; Mak, N. K.; Bünzli, J. C. G.; Jiang, L.; Long, N. J.; Lung, H. L.; Wong, K. L. Lanthanide-Based Peptide-Directed Visible/Near-Infrared Imaging and Inhibition of LMP1. *JACS Au* **2021**, *1* (7), 1034–1043.

NMR spectra of products

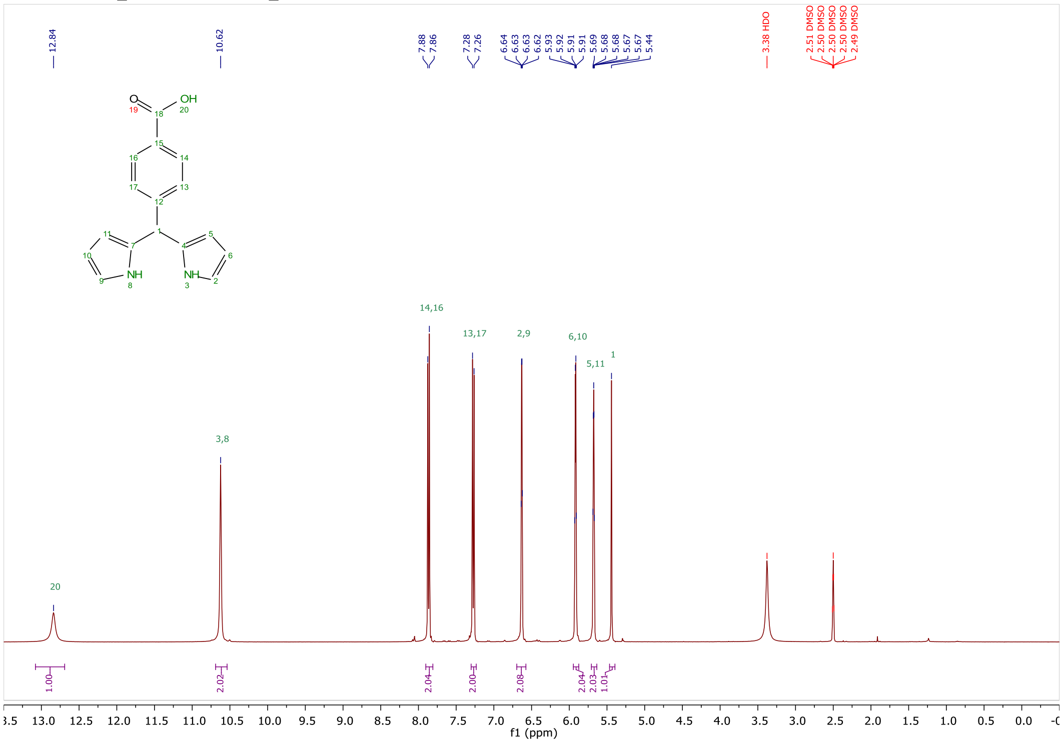

Figure S30. ^{1}H NMR spectrum of **1b** in DMSO-d_6.

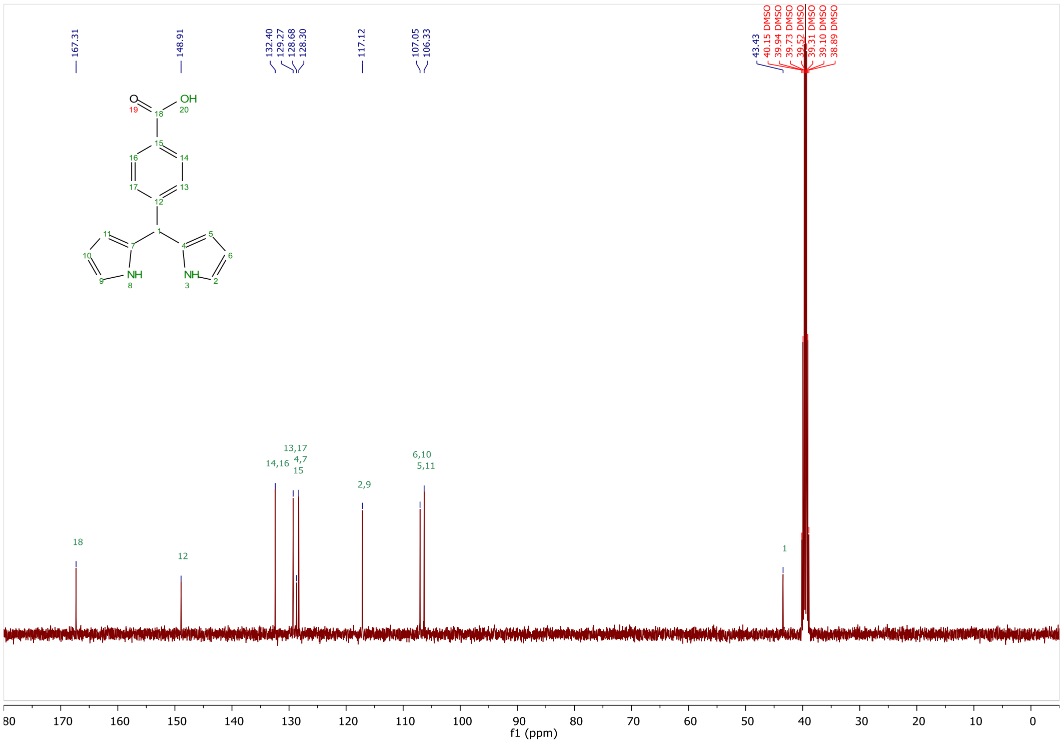

Figure S31. ^{13}C NMR spectrum of **1b** in DMSO-d_6.

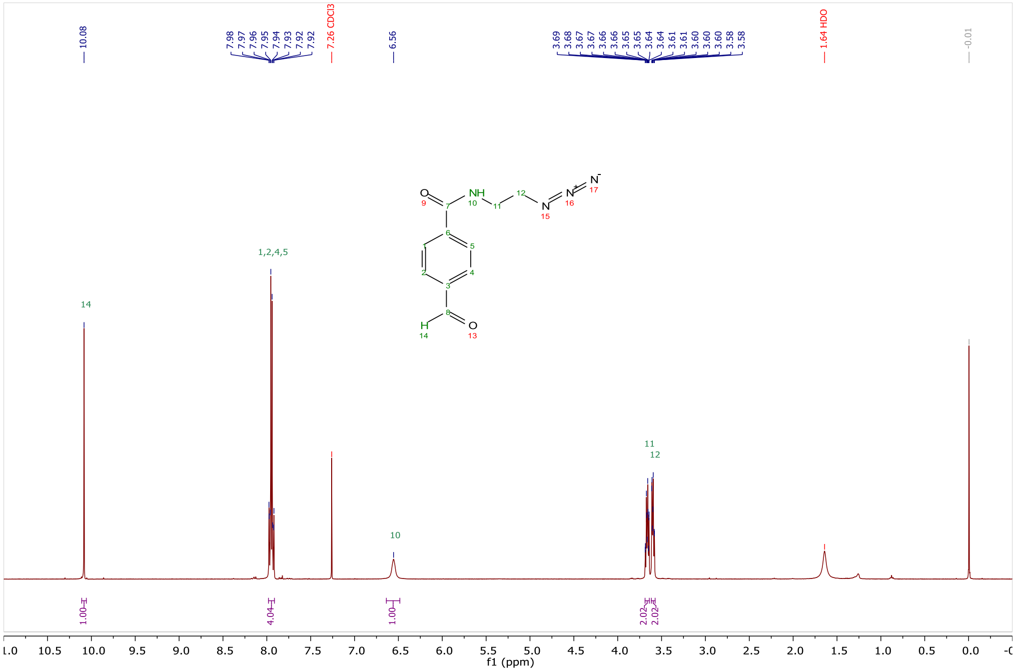

Figure S32. ^{1}H NMR spectrum of **2k** in $CDCl_3$.

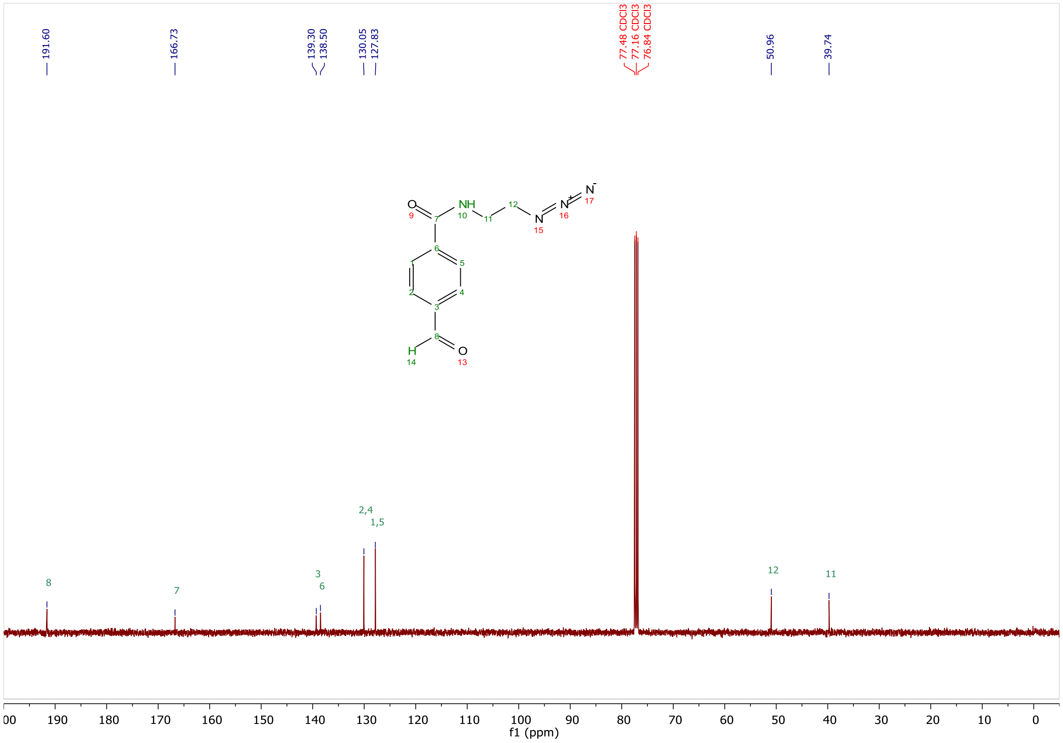

Figure S33. ^{13}C NMR spectrum of **2k** in $CDCl_3$.

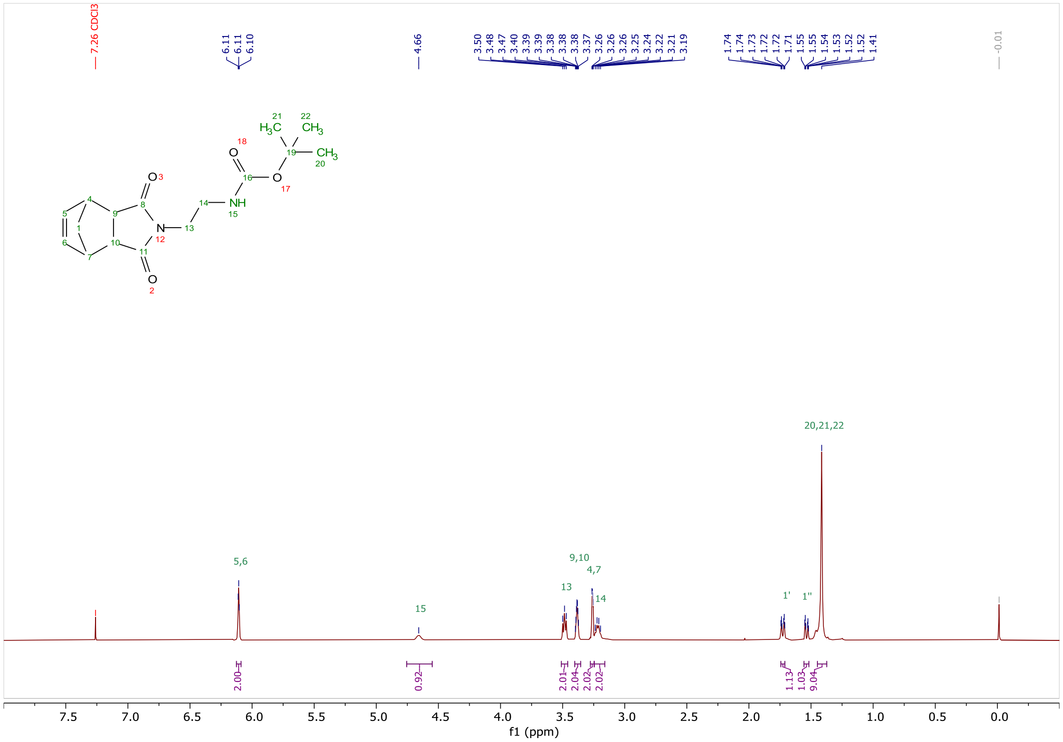

Figure S34. ^{1}H NMR spectrum of **S1** in $CDCl_3$.

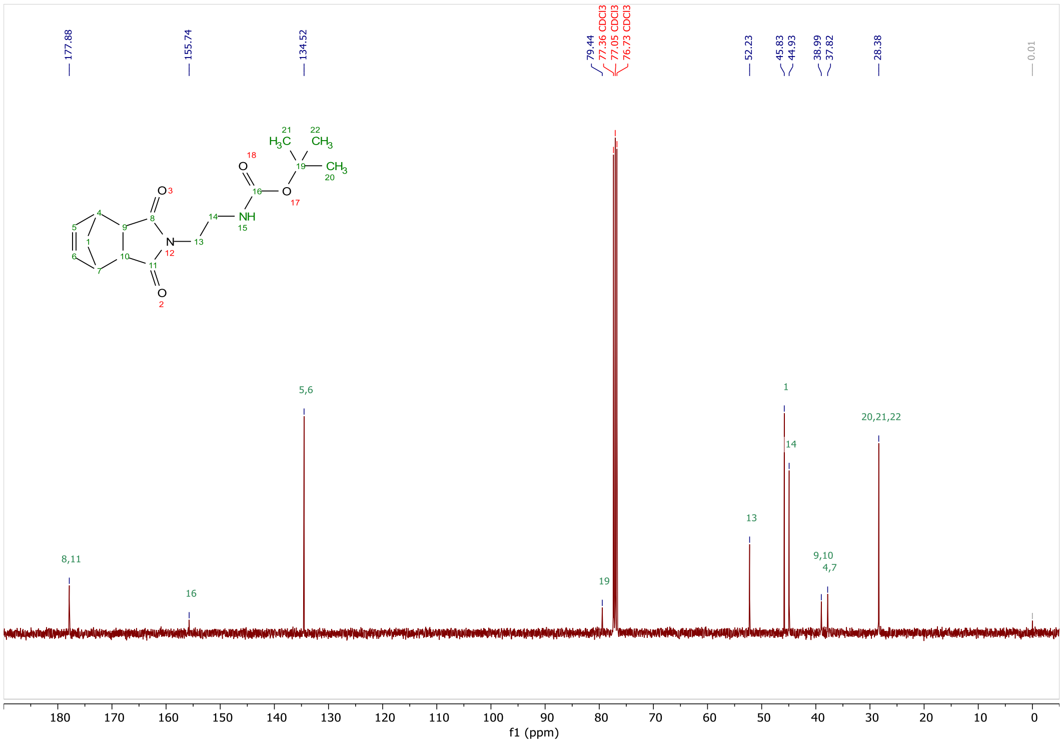

Figure S35. ^{13}C NMR spectrum of **S1** in $CDCl_3$.

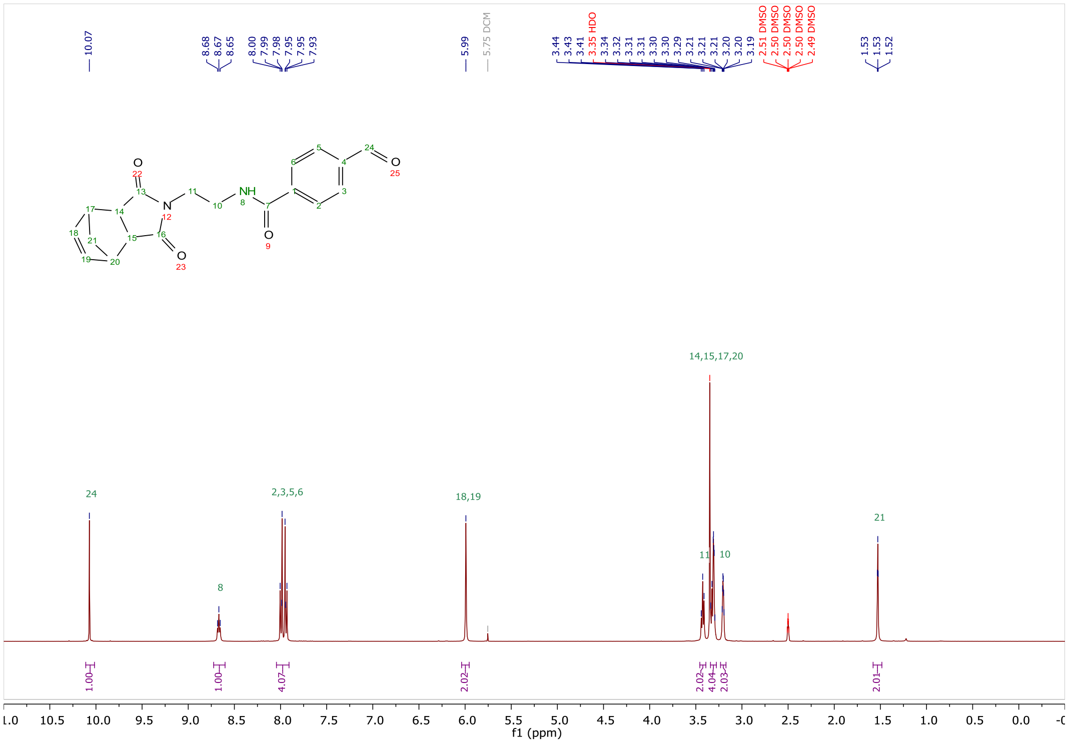

Figure S36. ^{1}H NMR spectrum of **2l** in DMSO-d_6.

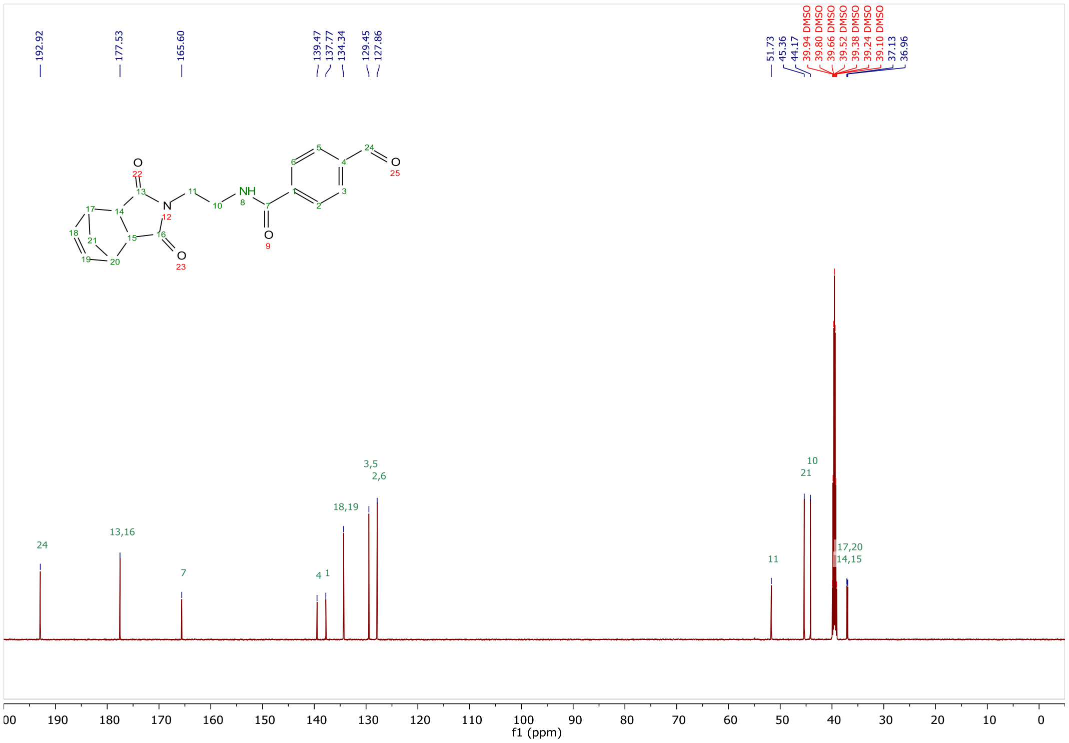

Figure S37. ^{13}C NMR spectrum of **2l** in DMSO-d_6.

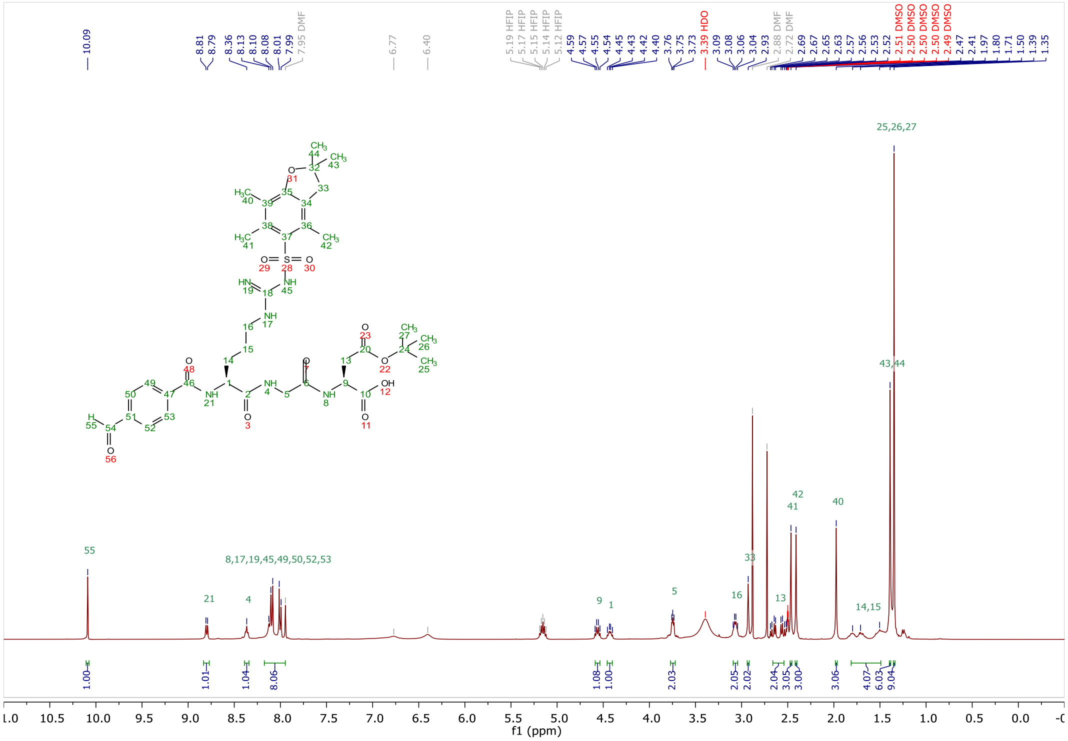

Figure S38. ^{1}H NMR spectrum of crude **1a**-Arg(Pbf)-Gly-Asp(^{t}Bu)-OH in DMSO-d_6.

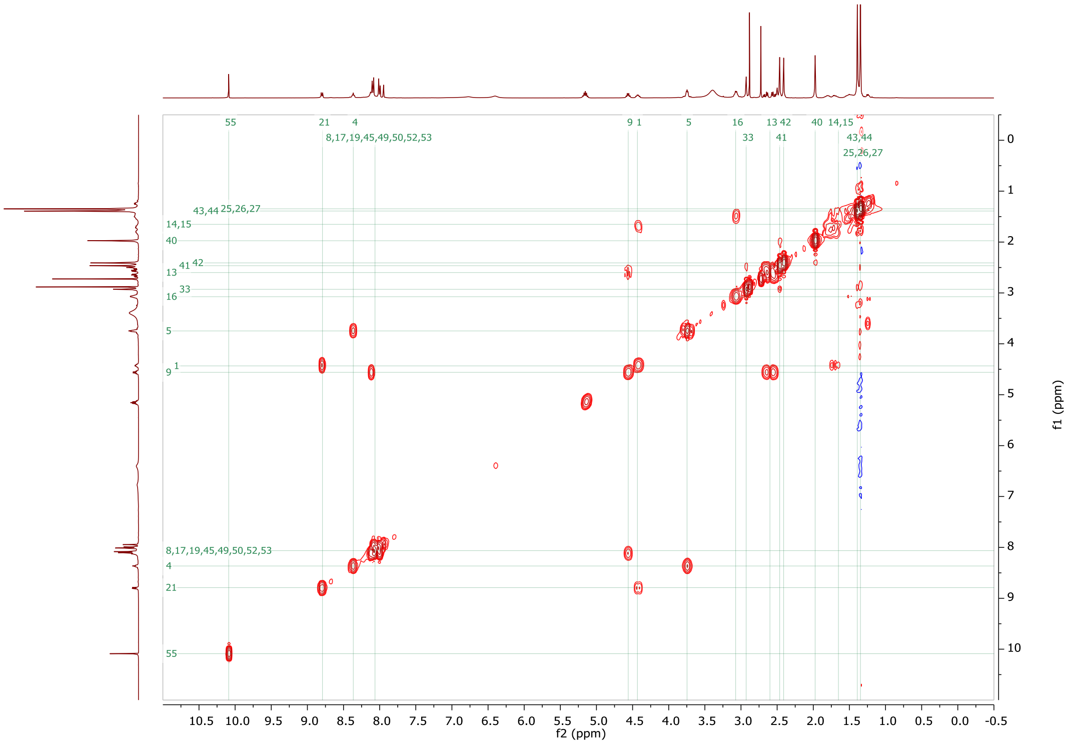

Figure S39. COSY spectrum of crude **1a**-Arg(Pbf)-Gly-Asp(^{t}Bu)-OH in DMSO-d_6.

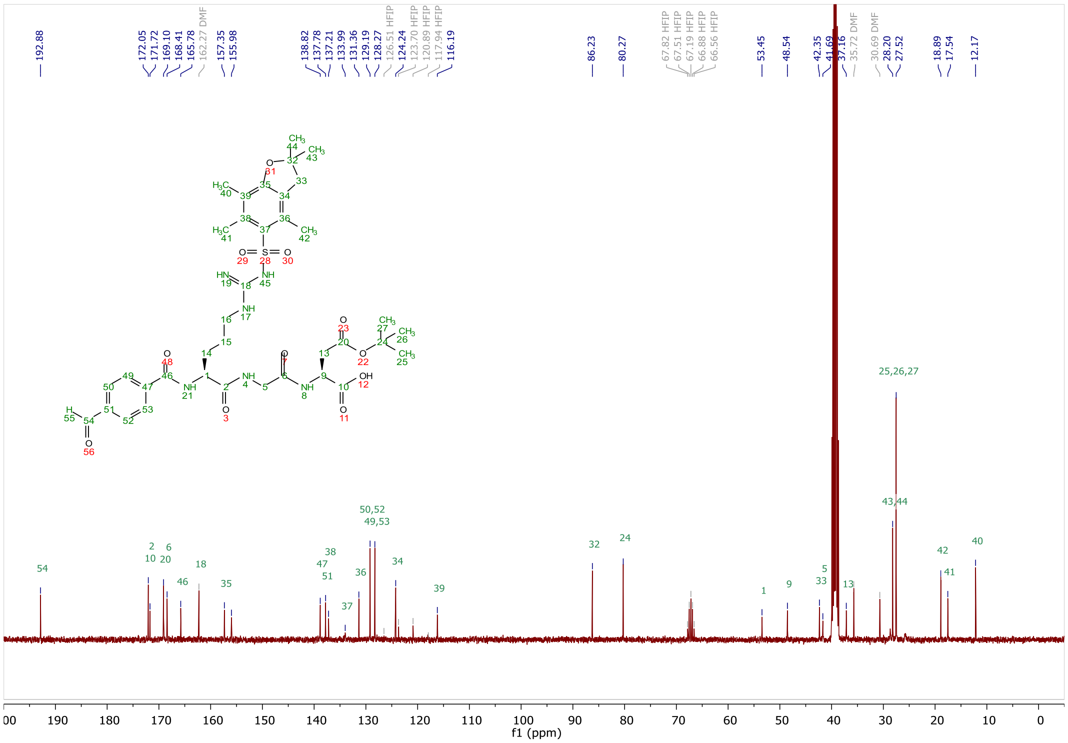

Figure S40. ^{13}C NMR spectrum of crude **1a**-Arg(Pbf)-Gly-Asp(^{t}Bu)-OH in DMSO-d_6.

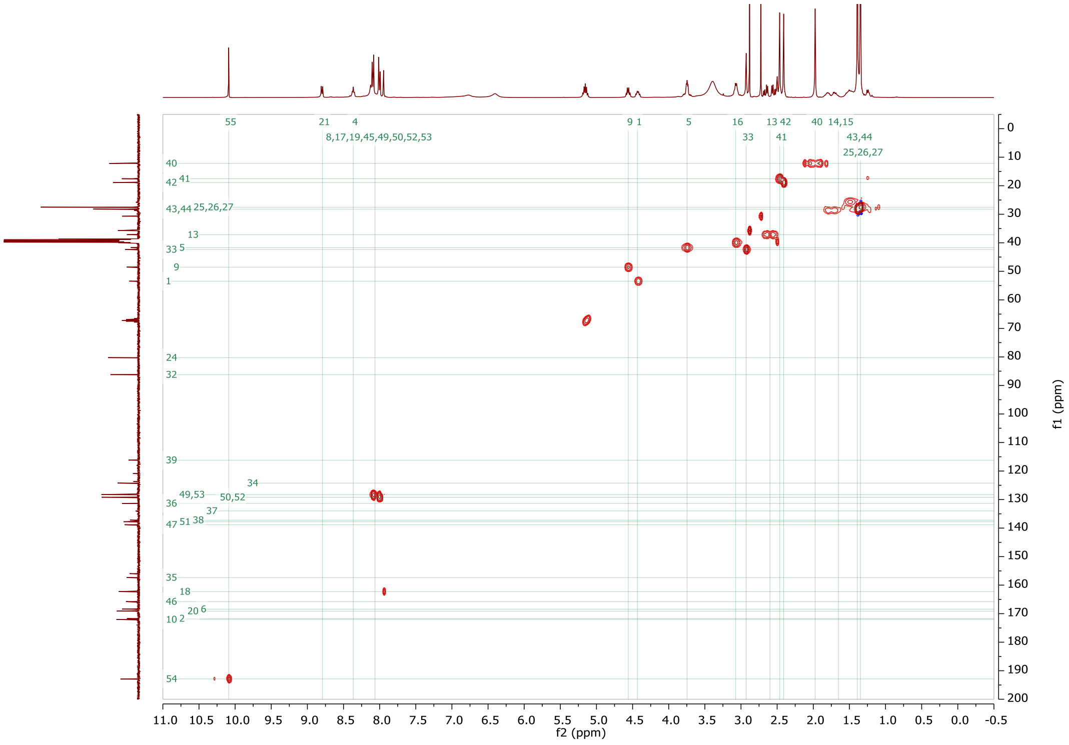

Figure S41. HSQC spectrum of crude **1a**-Arg(Pbf)-Gly-Asp(^{t}Bu)-OH in DMSO-d_6.

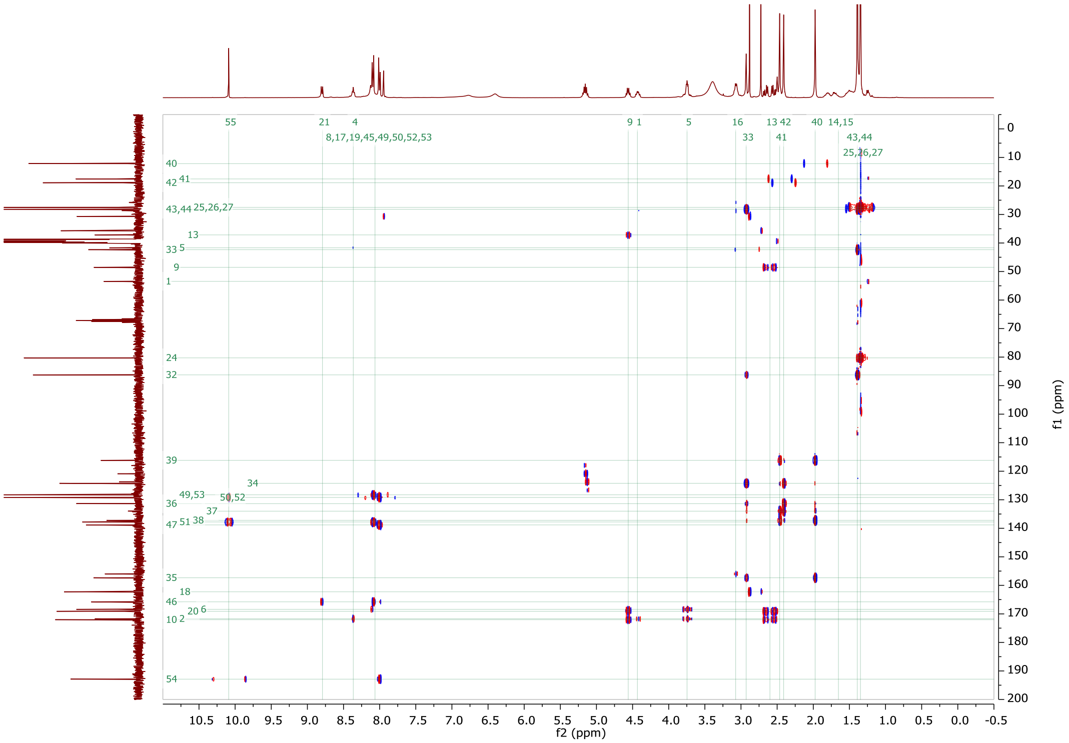

Figure S42. HMBC spectrum of crude **1a**-Arg(Pbf)-Gly-Asp(^{t}Bu)-OH in DMSO-d_6.

HPLC chromatograms and MALDI-TOF HRMS spectra of products

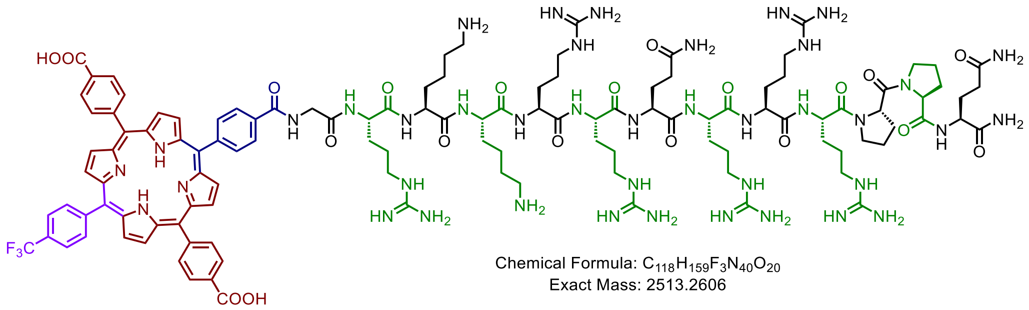

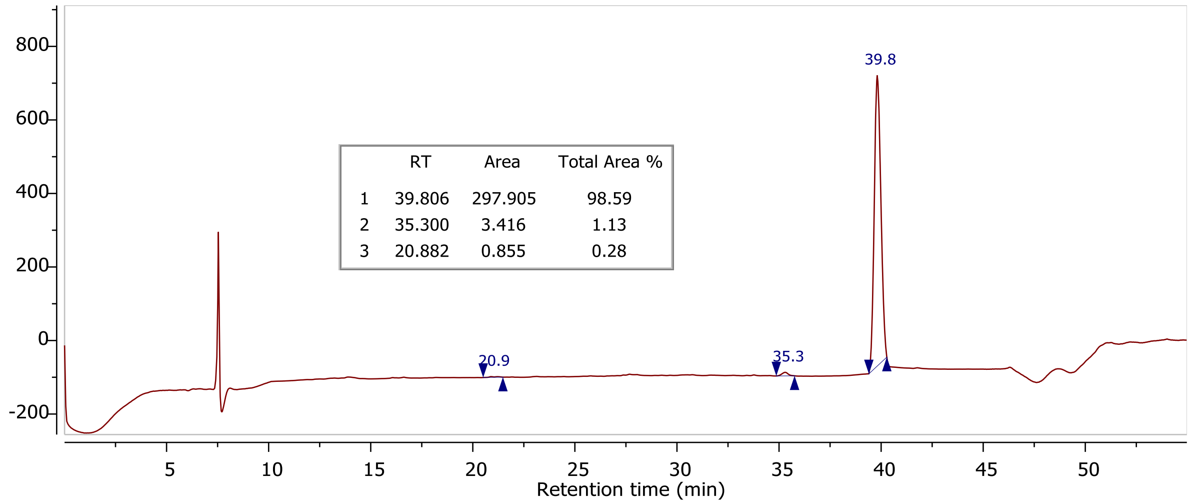

HPLC chromatogram of **4a**.

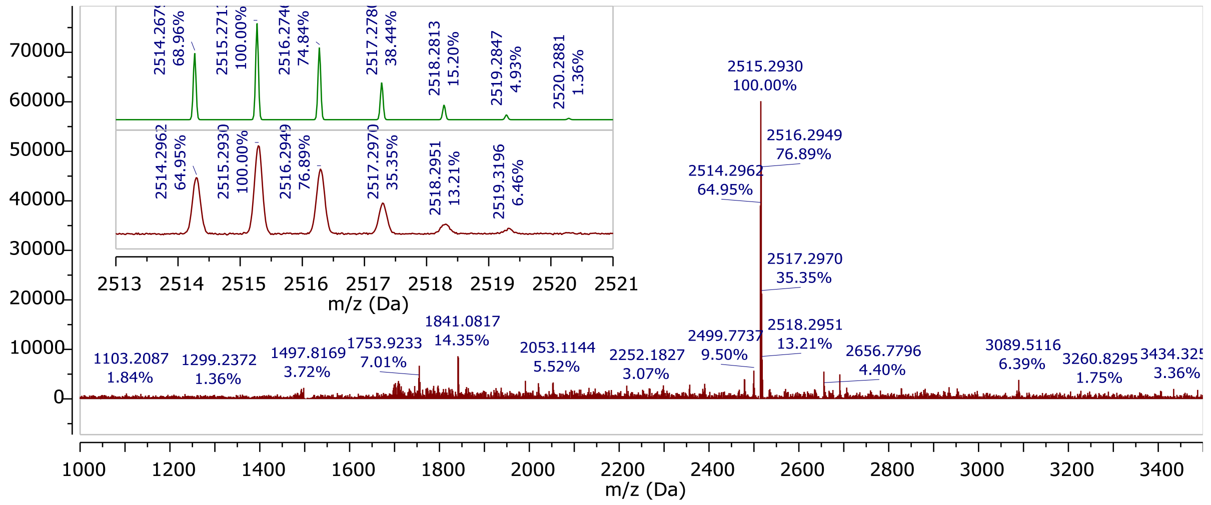

HRMS (MALDI-TOF) spectrum of **4a**.

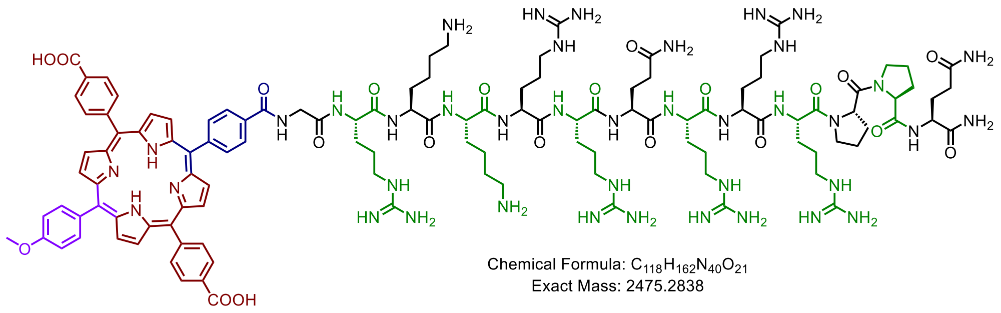

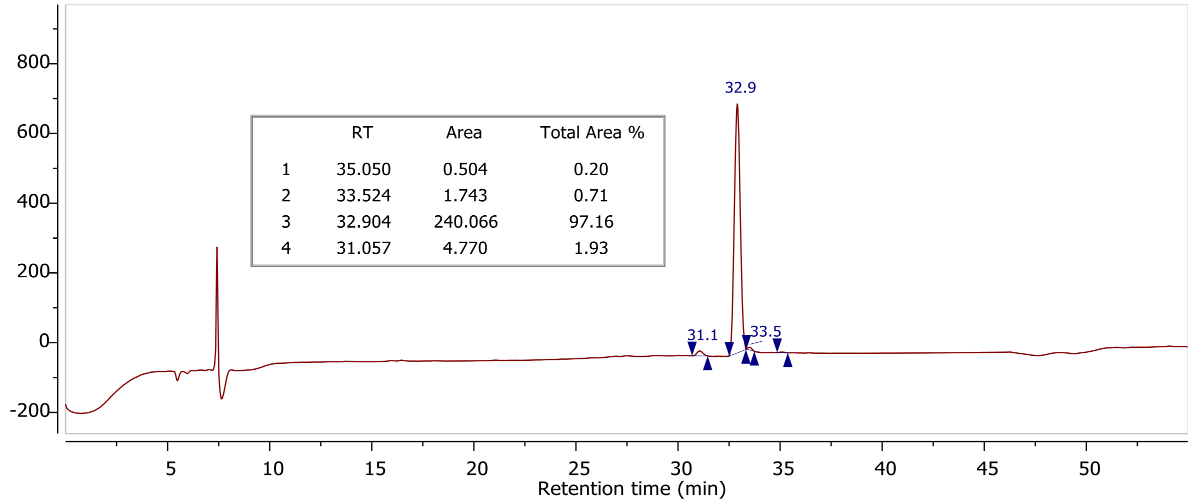

	RT	Area	Total Area %
1	35.050	0.504	0.20
2	33.524	1.743	0.71
3	32.904	240.066	97.16
4	31.057	4.770	1.93

HPLC chromatogram of **4b**.

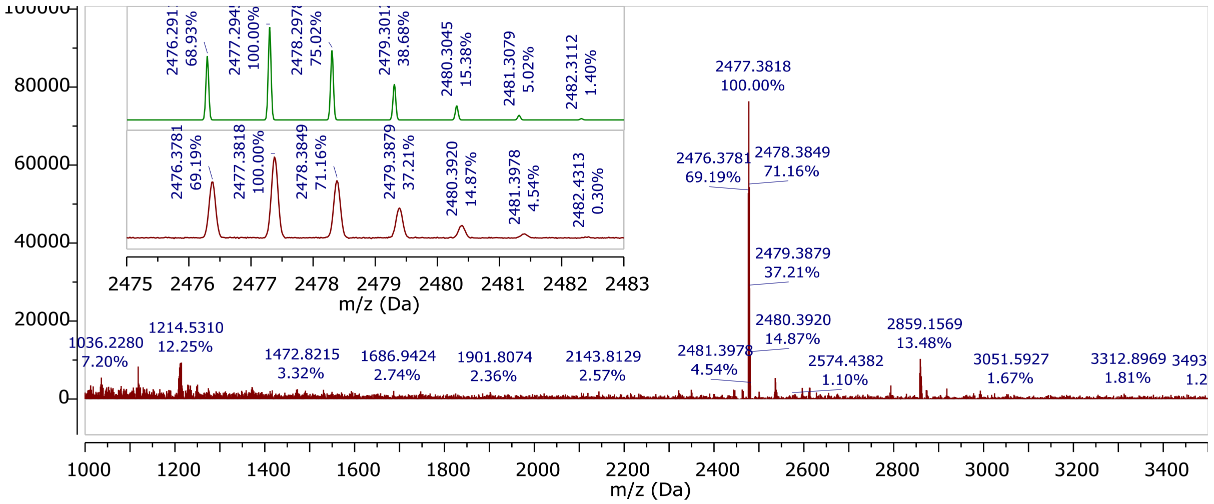

HRMS (MALDI-TOF) spectrum of **4b**.

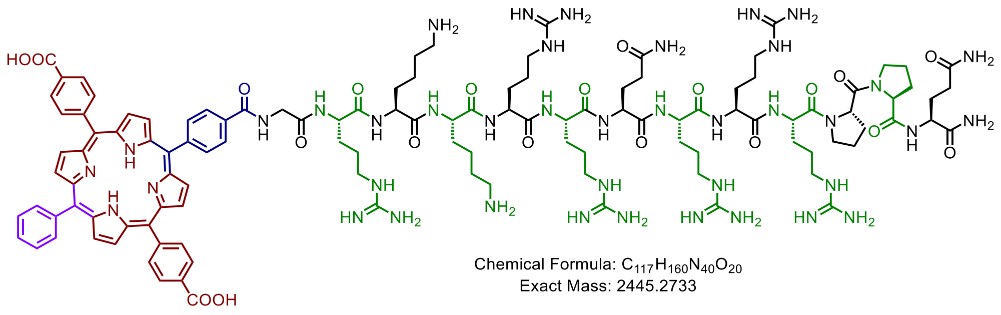

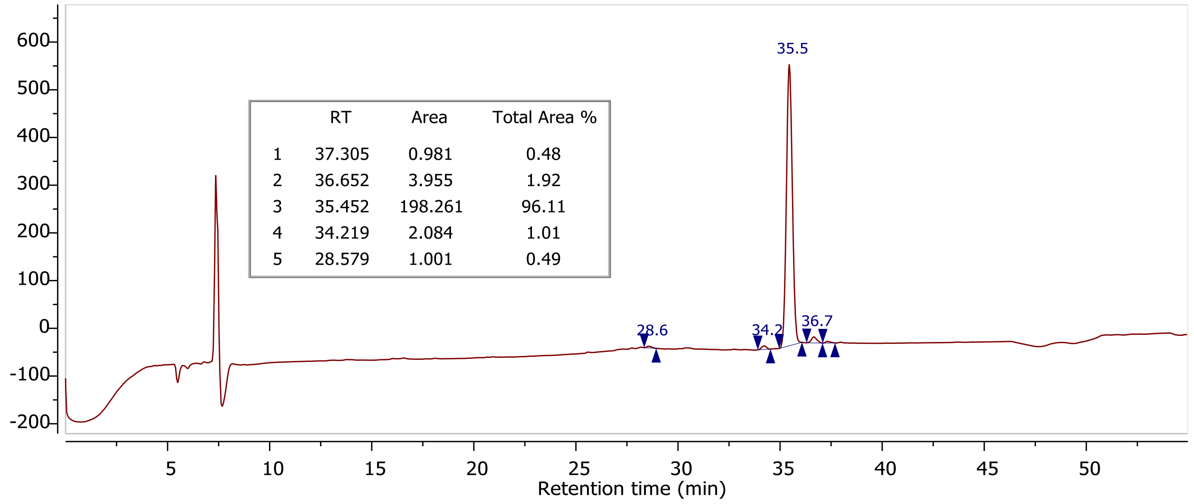

HPLC chromatogram of **4c**.

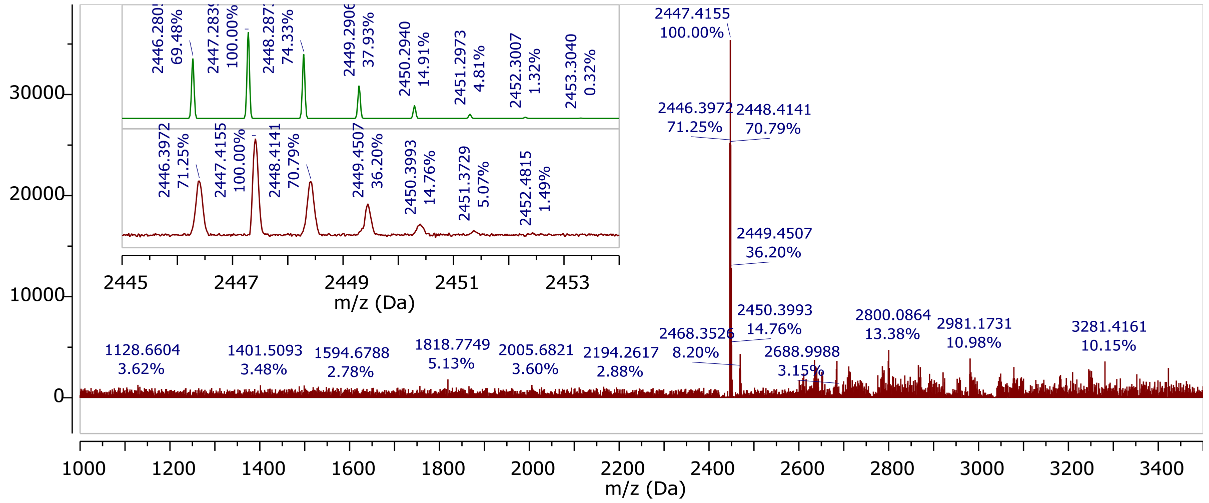

HRMS (MALDI-TOF) spectrum of **4c**.

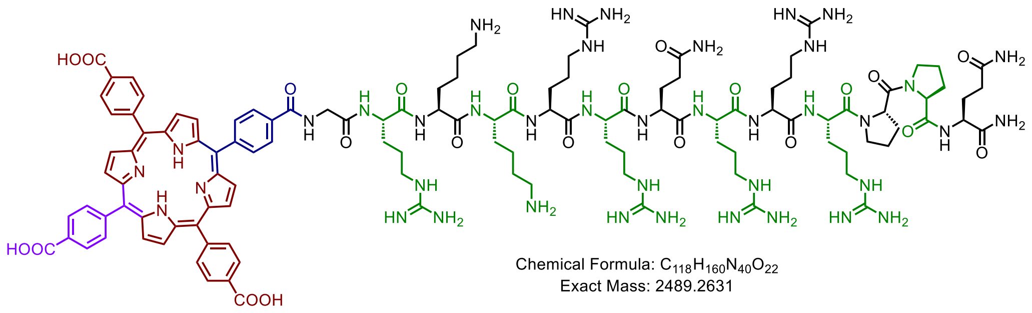

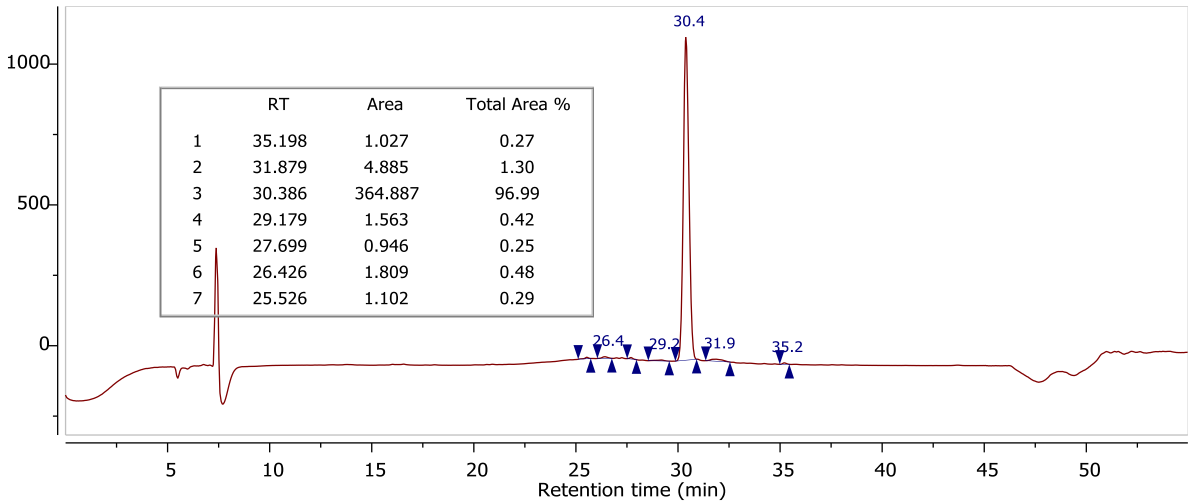

	RT	Area	Total Area %
1	35.198	1.027	0.27
2	31.879	4.885	1.30
3	30.386	364.887	96.99
4	29.179	1.563	0.42
5	27.699	0.946	0.25
6	26.426	1.809	0.48
7	25.526	1.102	0.29

HPLC chromatogram of **4d**.

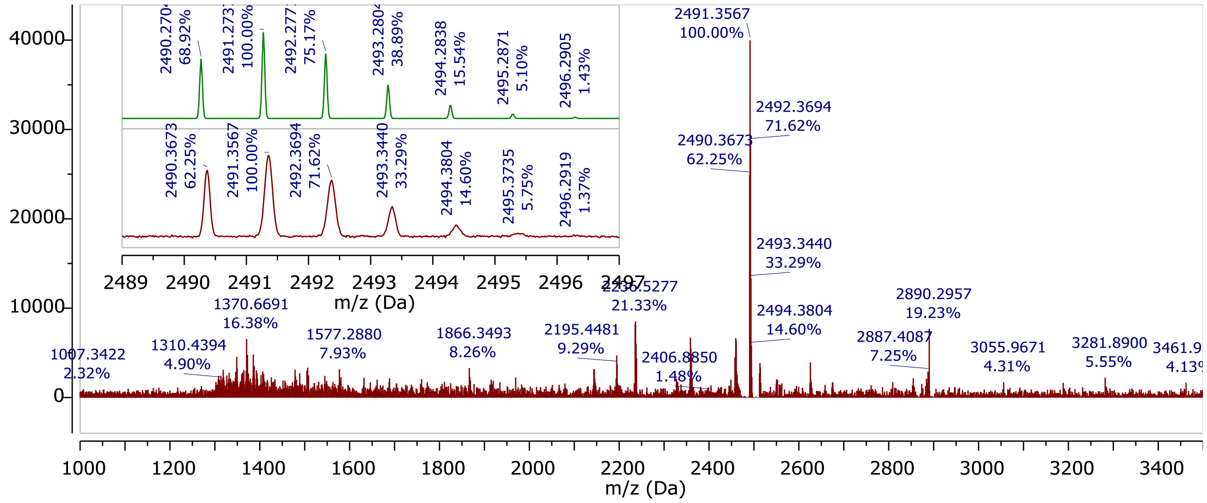

HRMS (MALDI-TOF) spectrum of **4d**.

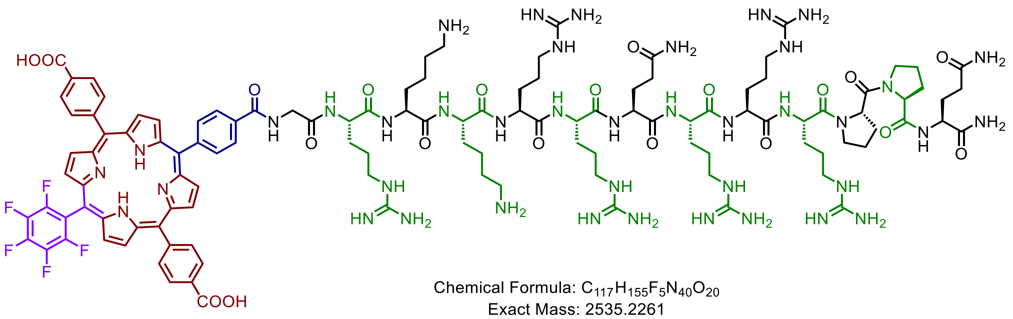

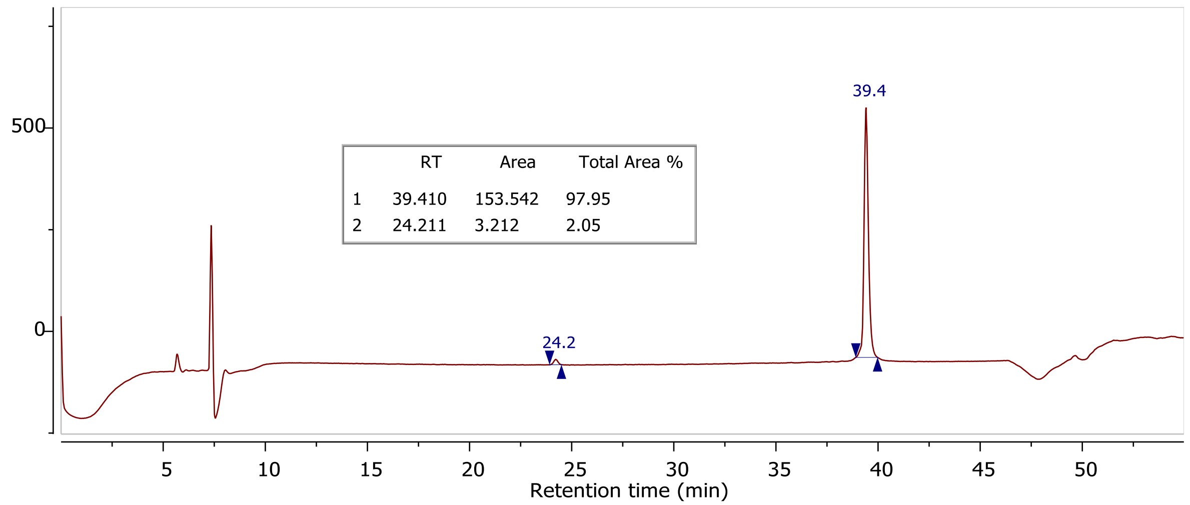

	RT	Area	Total Area %
1	39.410	153.542	97.95
2	24.211	3.212	2.05

HPLC chromatogram of **4e**.

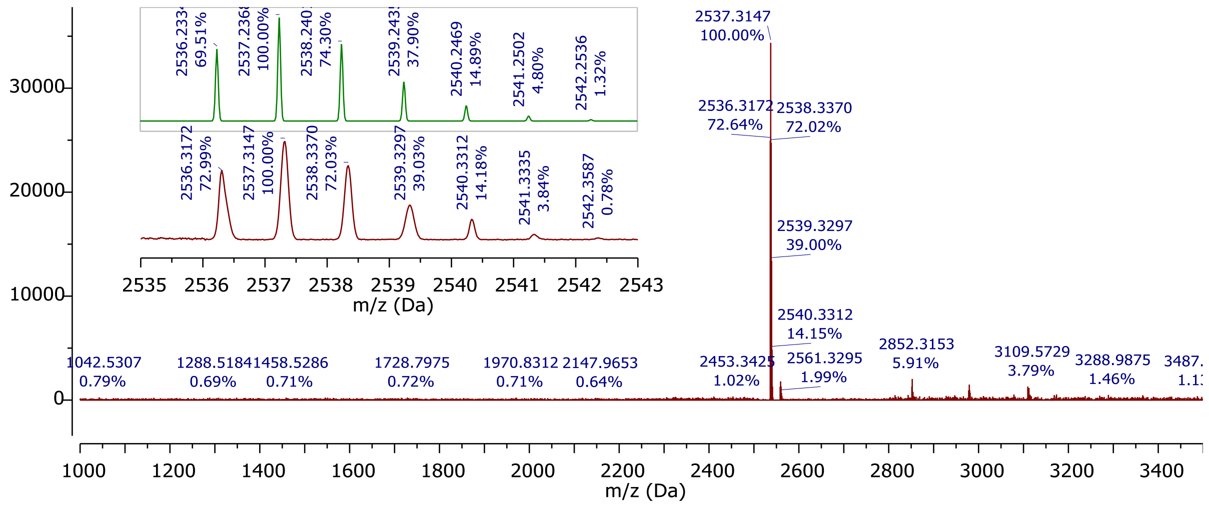

HRMS (MALDI-TOF) spectrum of **4e**.

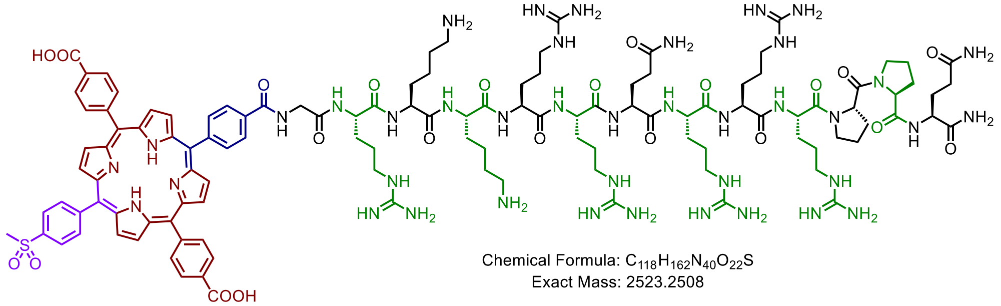

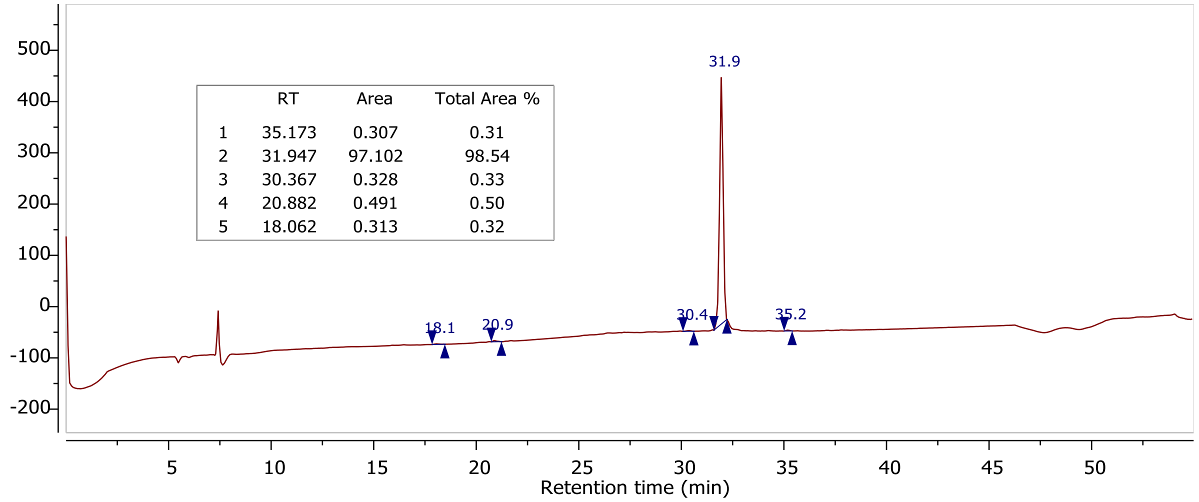

	RT	Area	Total Area %
1	35.173	0.307	0.31
2	31.947	97.102	98.54
3	30.367	0.328	0.33
4	20.882	0.491	0.50
5	18.062	0.313	0.32

HPLC chromatogram of **4f**.

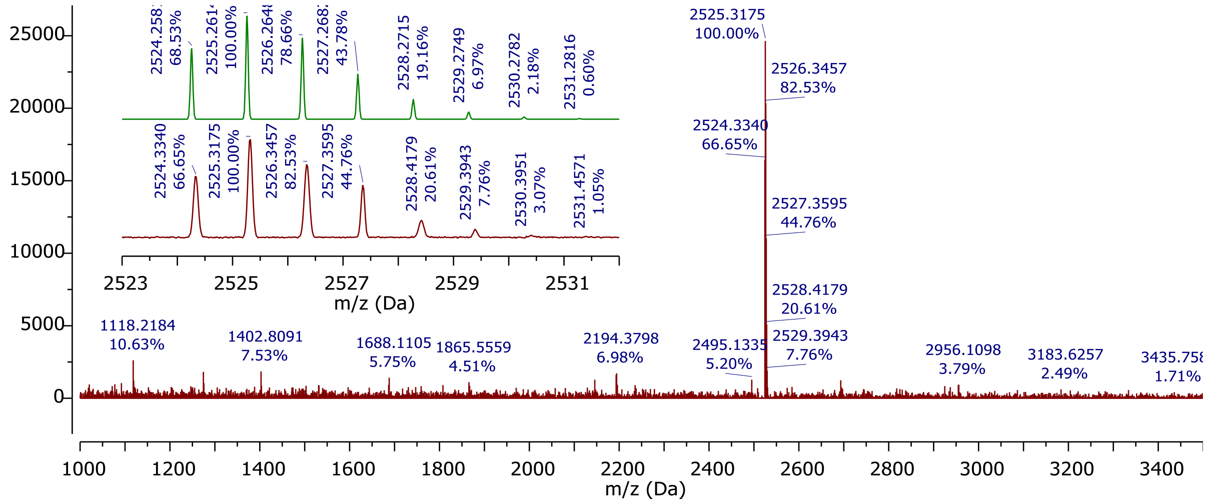

HRMS (MALDI-TOF) spectrum of **4f**.

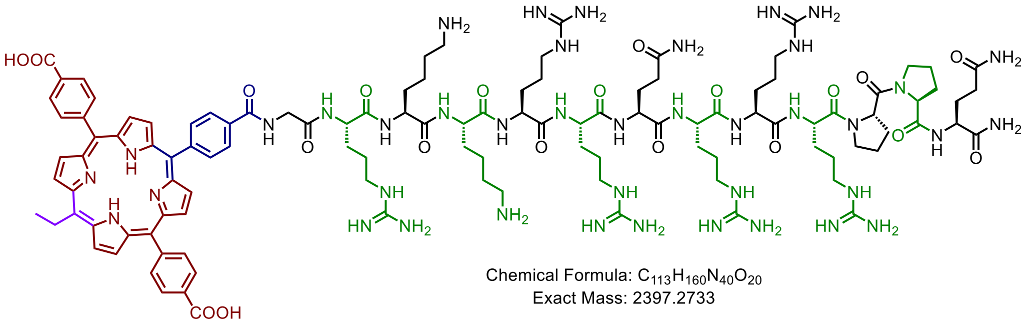

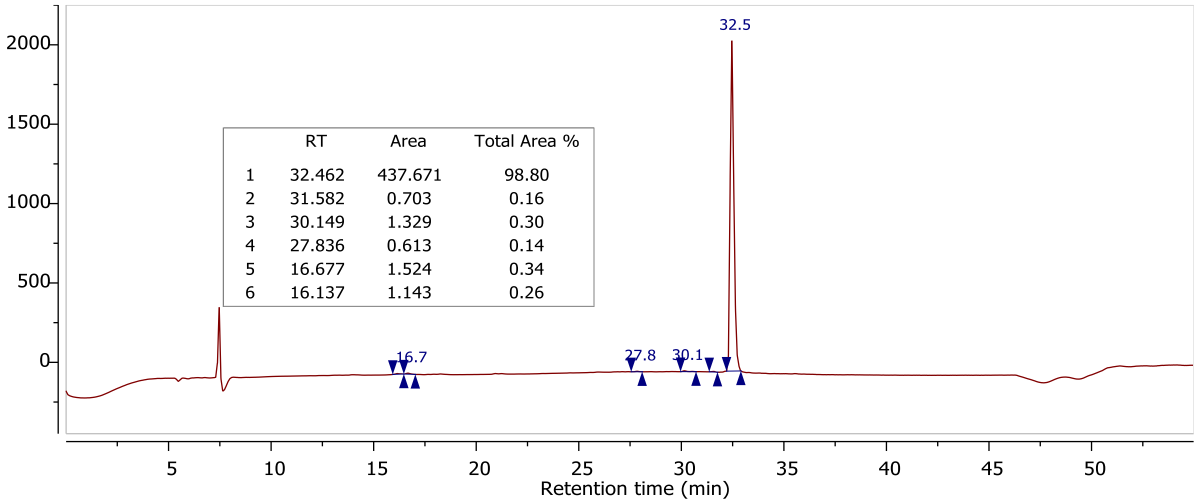

	RT	Area	Total Area %
1	32.462	437.671	98.80
2	31.582	0.703	0.16
3	30.149	1.329	0.30
4	27.836	0.613	0.14
5	16.677	1.524	0.34
6	16.137	1.143	0.26

HPLC chromatogram of **4g**.

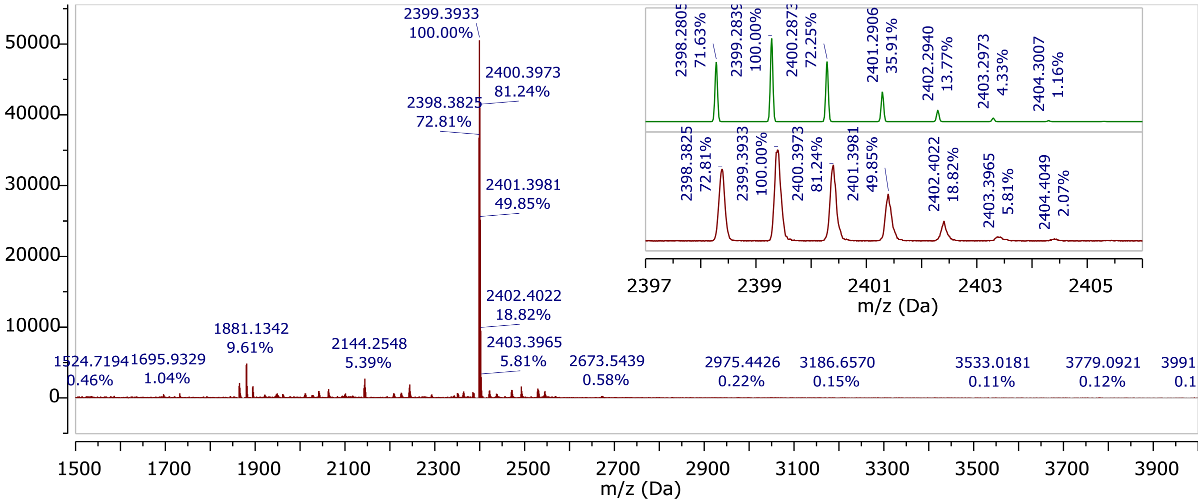

HRMS (MALDI-TOF) spectrum of **4g**.

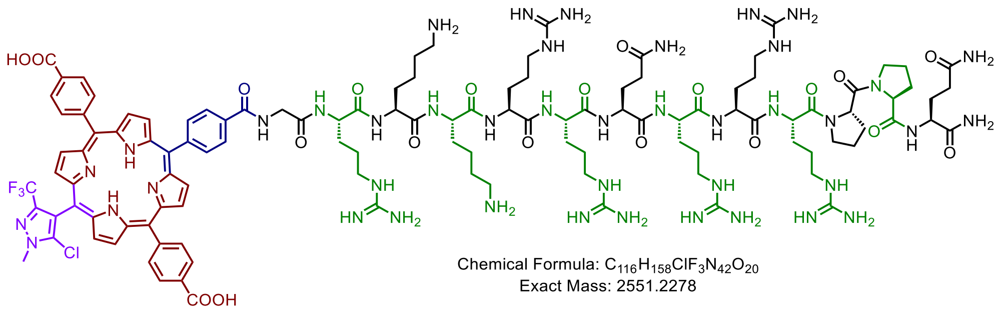

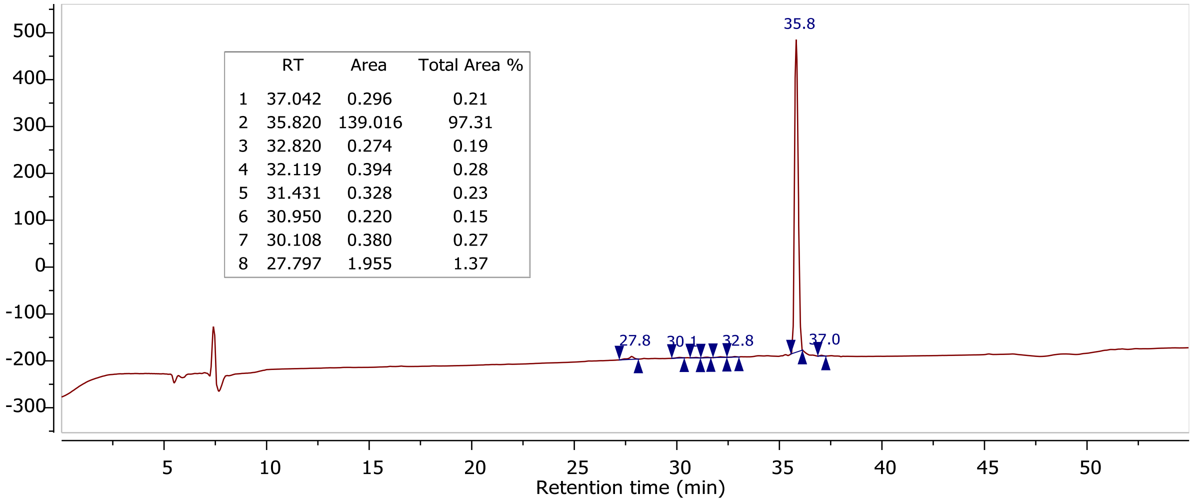

	RT	Area	Total Area %
1	37.042	0.296	0.21
2	35.820	139.016	97.31
3	32.820	0.274	0.19
4	32.119	0.394	0.28
5	31.431	0.328	0.23
6	30.950	0.220	0.15
7	30.108	0.380	0.27
8	27.797	1.955	1.37

HPLC chromatogram of **4h**.

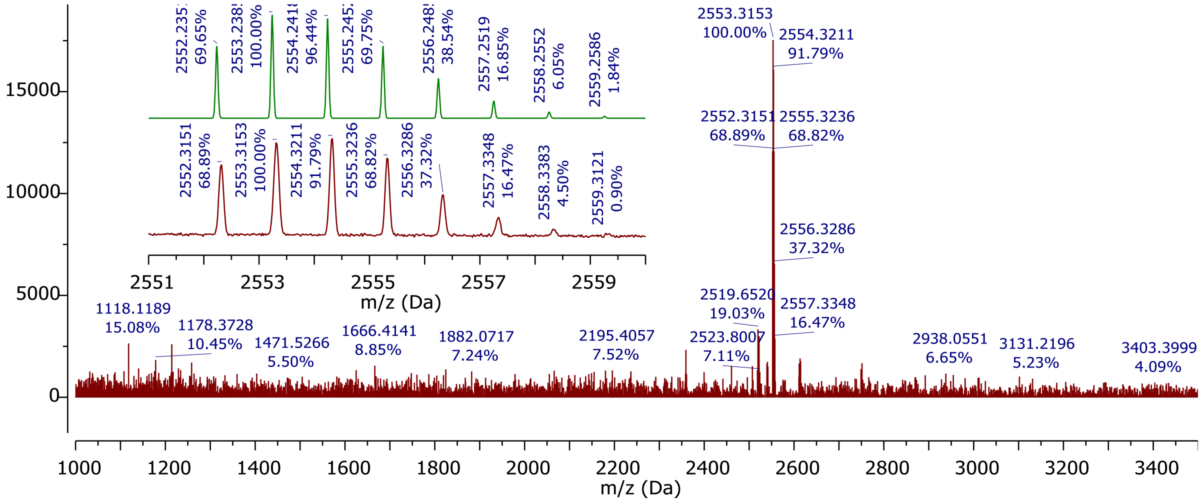

HRMS (MALDI-TOF) spectrum of **4h**.

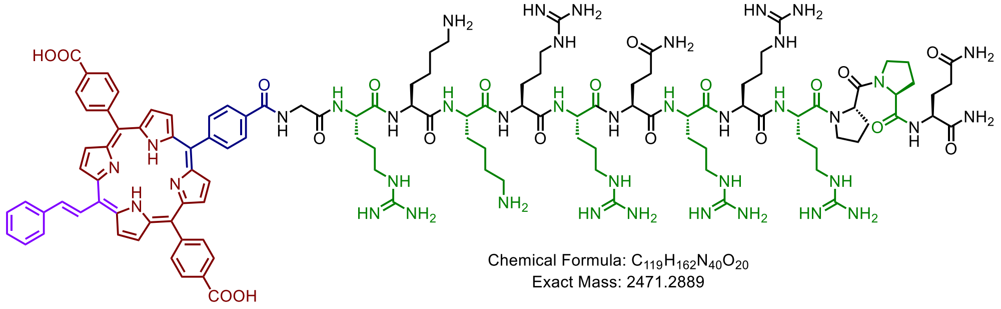

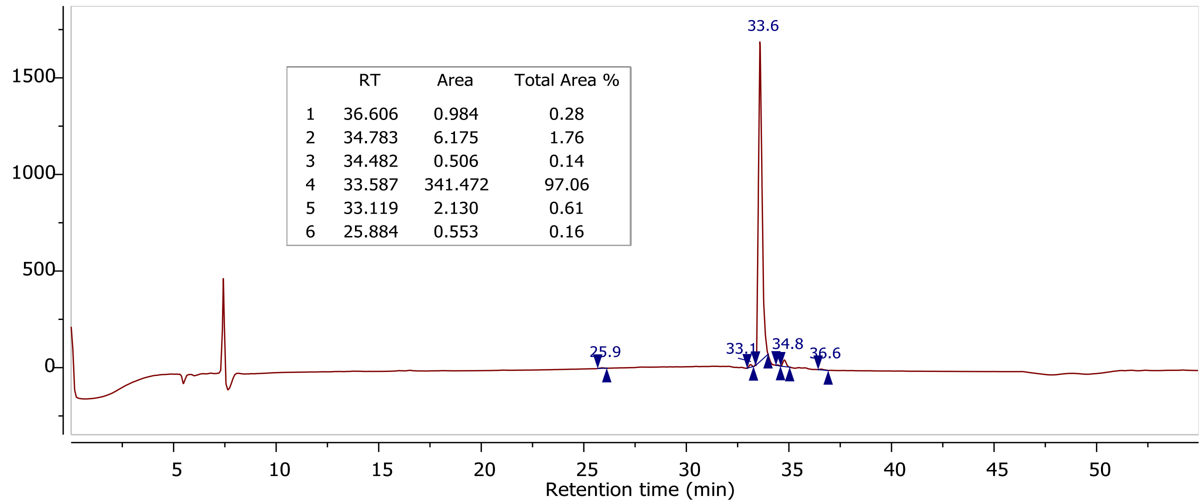

	RT	Area	Total Area %
1	36.606	0.984	0.28
2	34.783	6.175	1.76
3	34.482	0.506	0.14
4	33.587	341.472	97.06
5	33.119	2.130	0.61
6	25.884	0.553	0.16

HPLC chromatogram of **4i**.

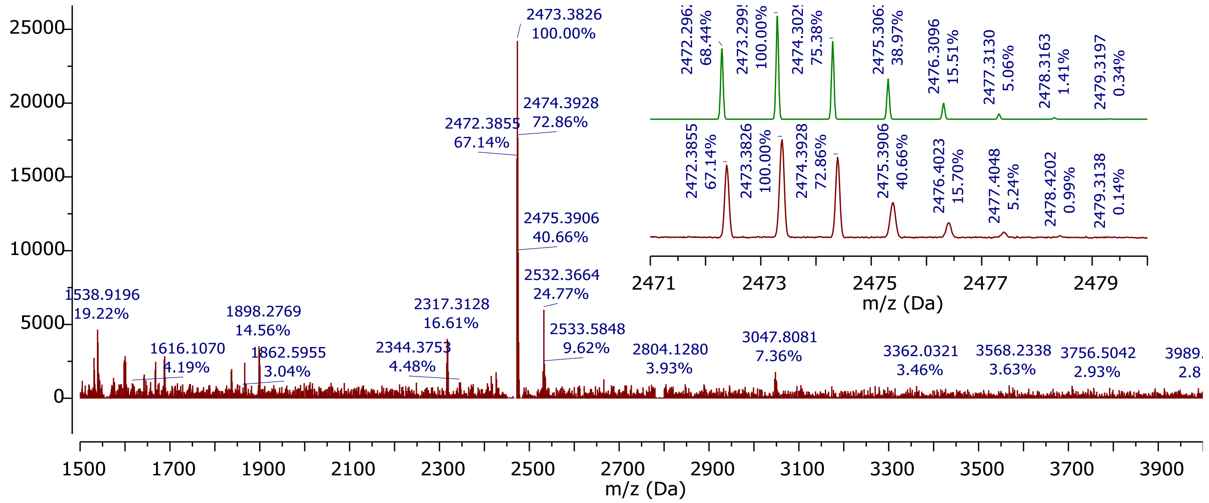

HRMS (MALDI-TOF) spectrum of **4i**.

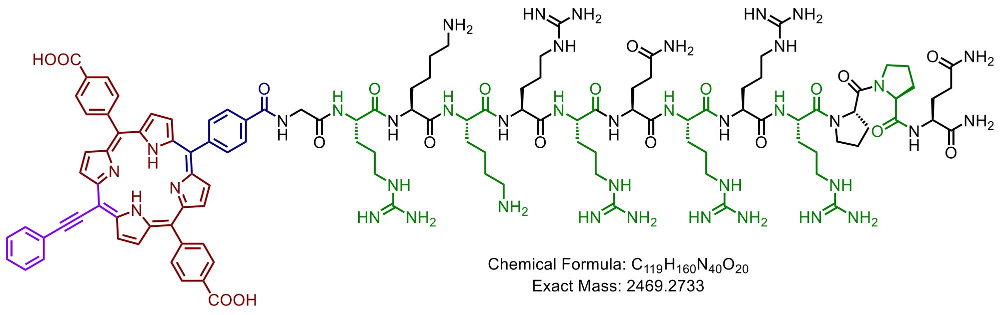

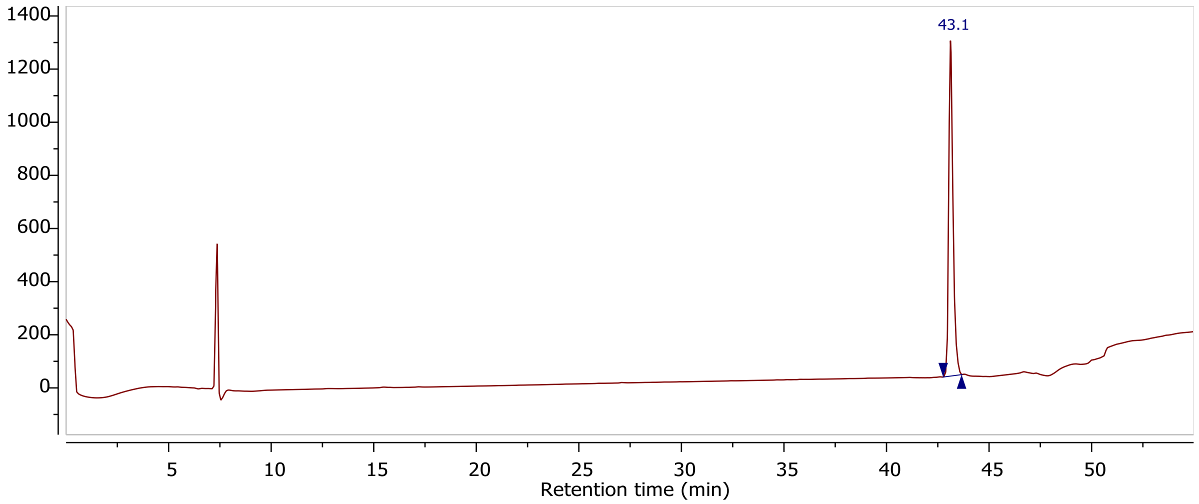

HPLC chromatogram of **4j**.

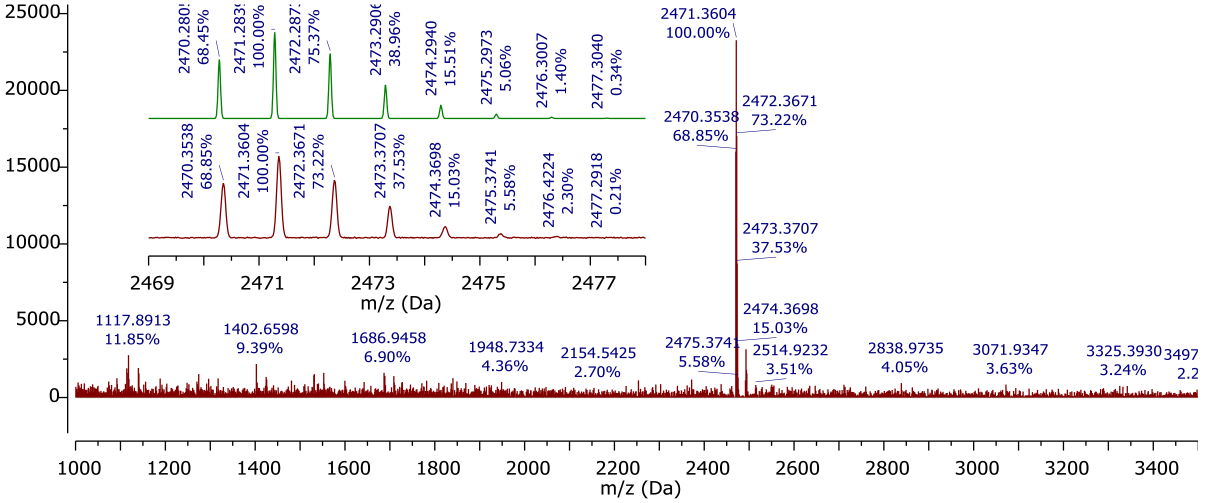

HRMS (MALDI-TOF) spectrum of **4j**.

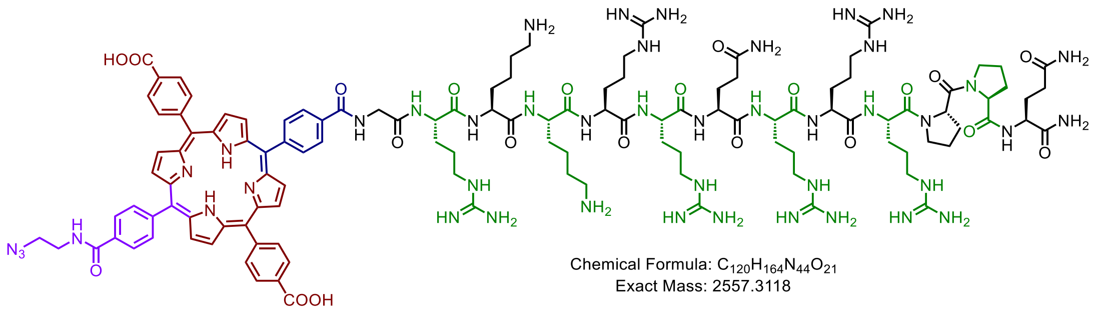

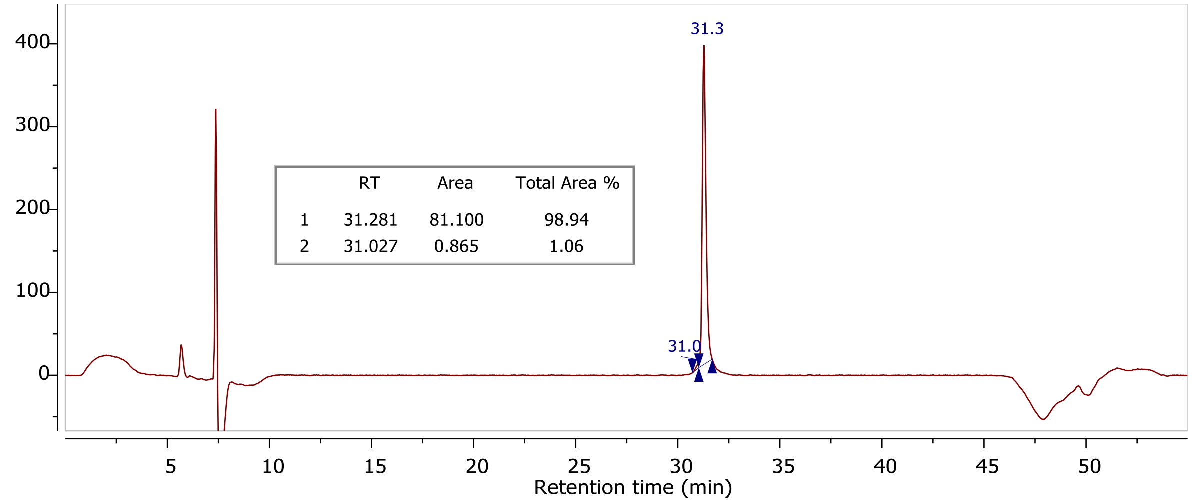

	RT	Area	Total Area %
1	31.281	81.100	98.94
2	31.027	0.865	1.06

HPLC chromatogram of **4k**.

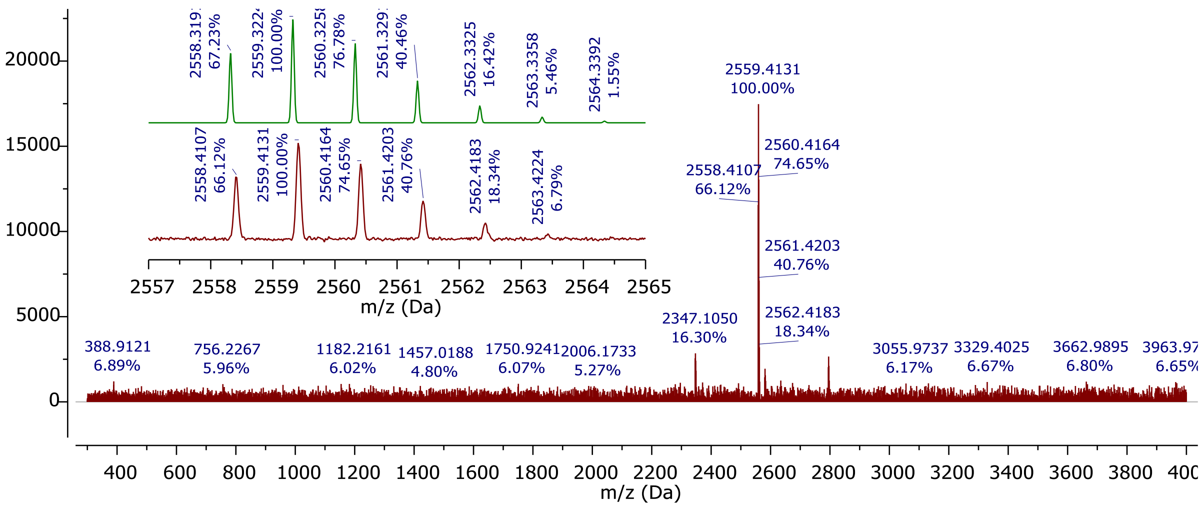

HRMS (MALDI-TOF) spectrum of **4k**.

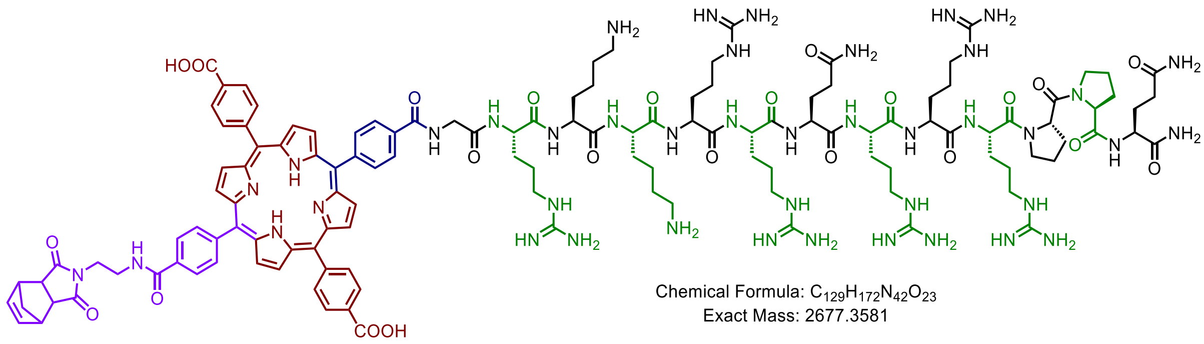

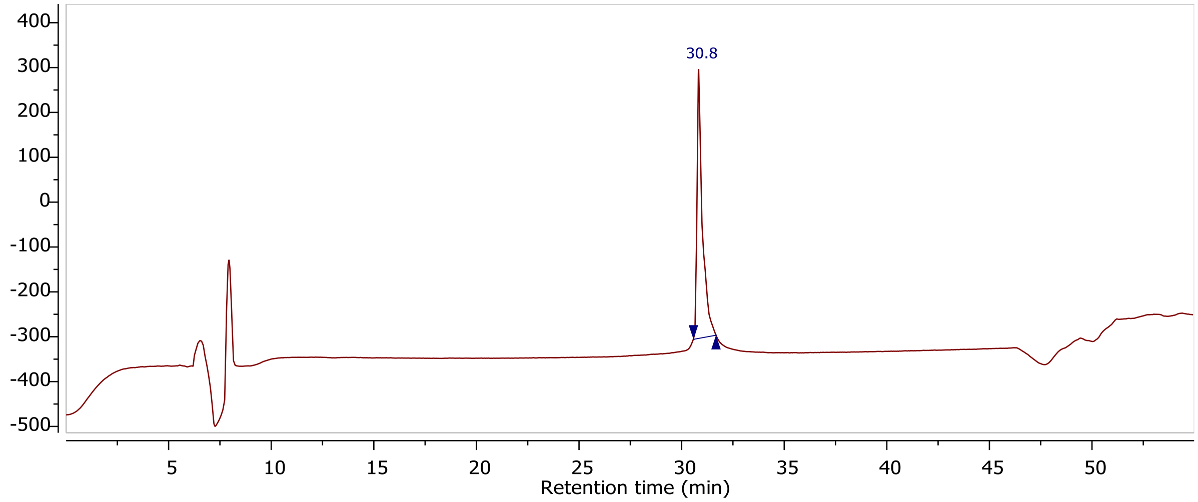

HPLC chromatogram of **4l**.

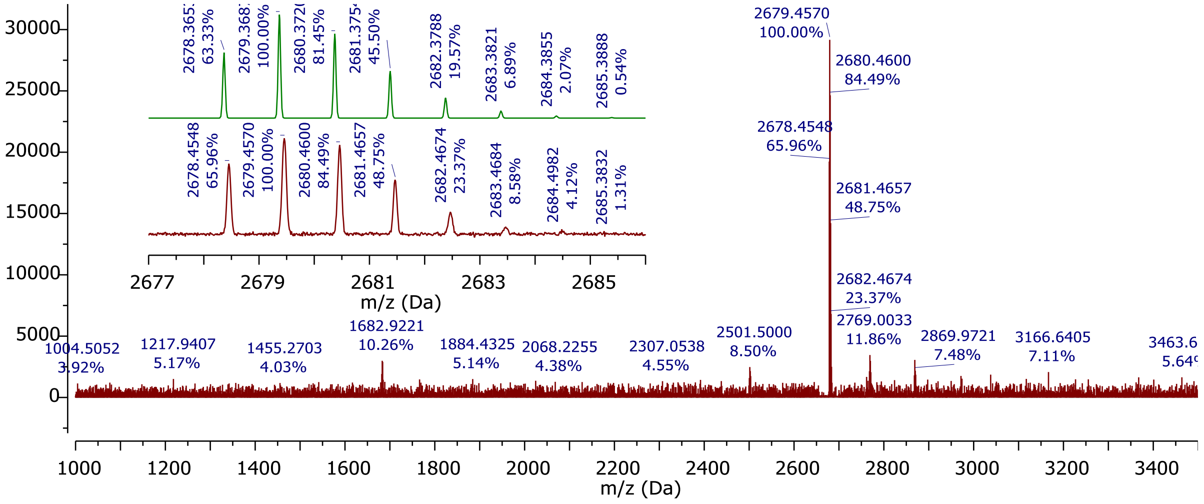

HRMS (MALDI-TOF) spectrum of **4l**.

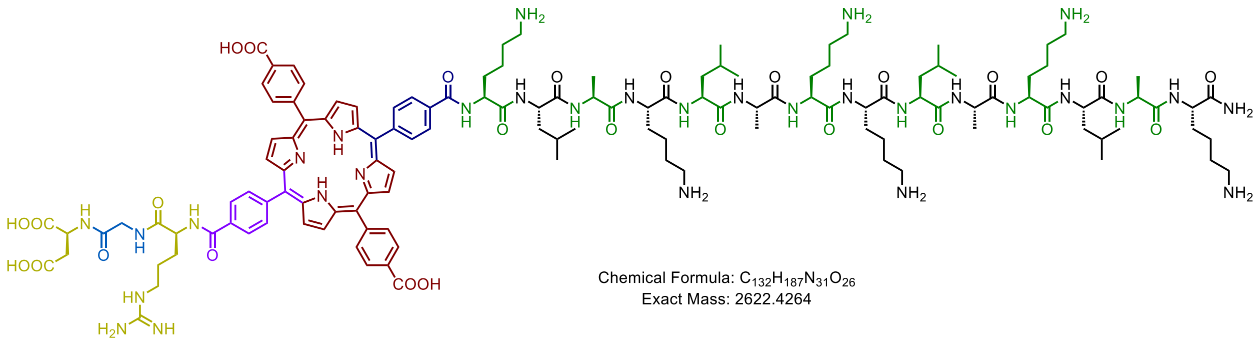

Chemical Formula: $C_{132}H_{187}N_{31}O_{26}$
Exact Mass: 2622.4264

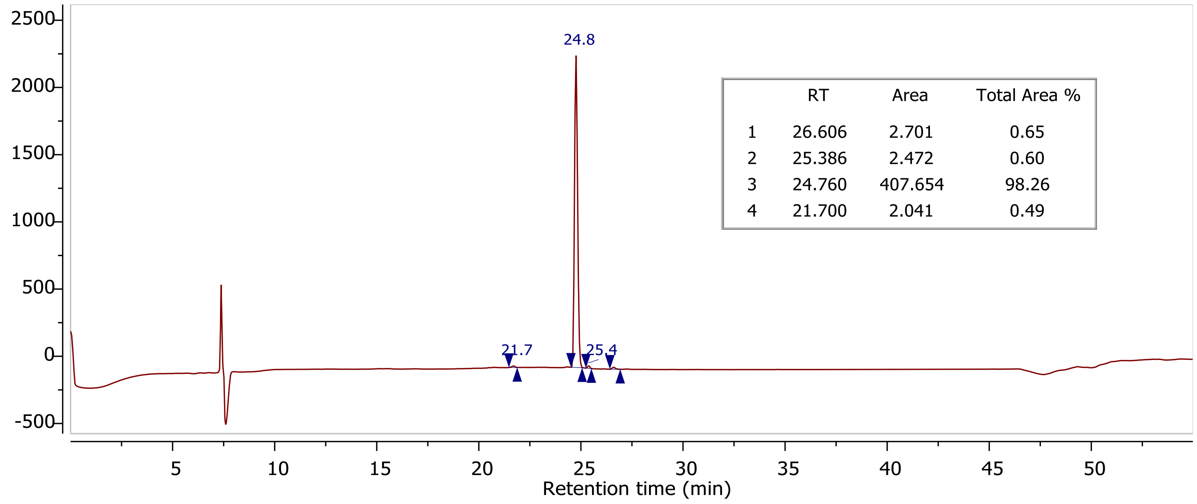

HPLC chromatogram of **4m**.

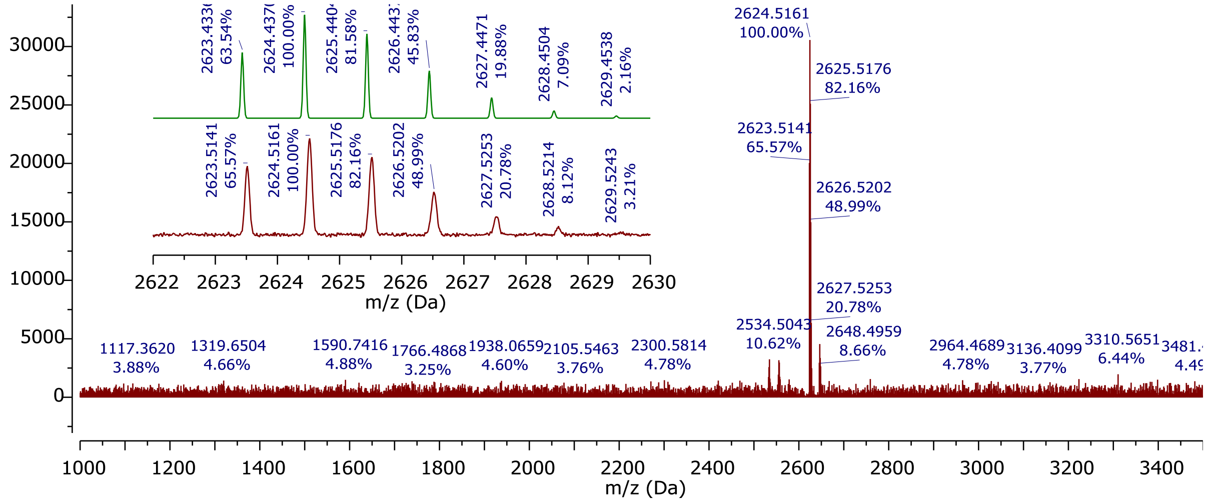

HRMS (MALDI-TOF) spectrum of **4m**.

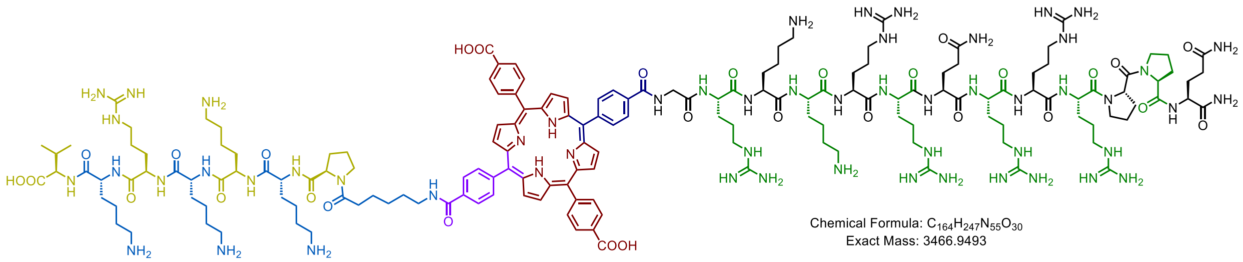

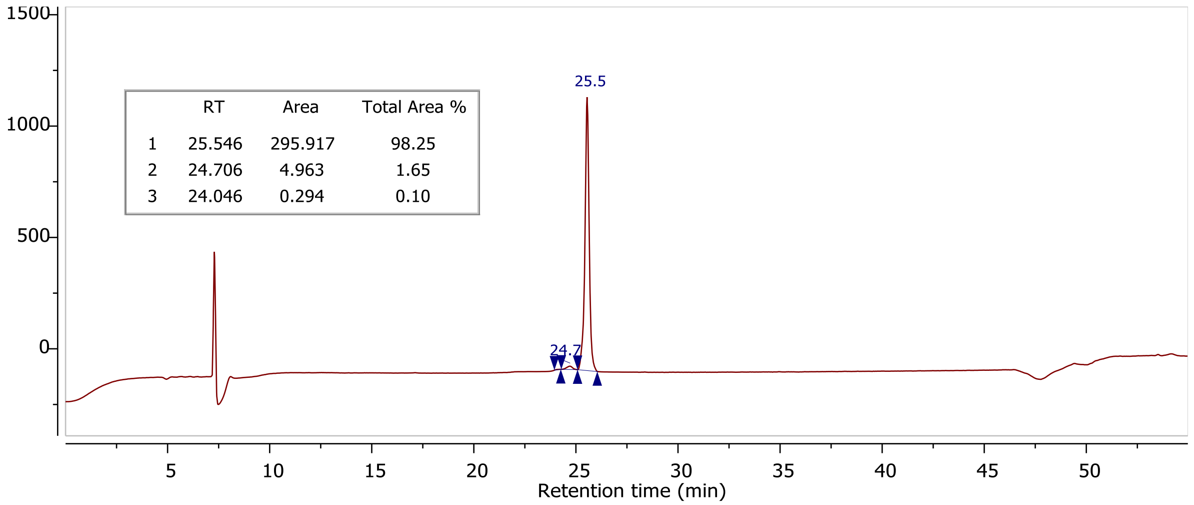

	RT	Area	Total Area %
1	25.546	295.917	98.25
2	24.706	4.963	1.65
3	24.046	0.294	0.10

HPLC chromatogram of **4n**.

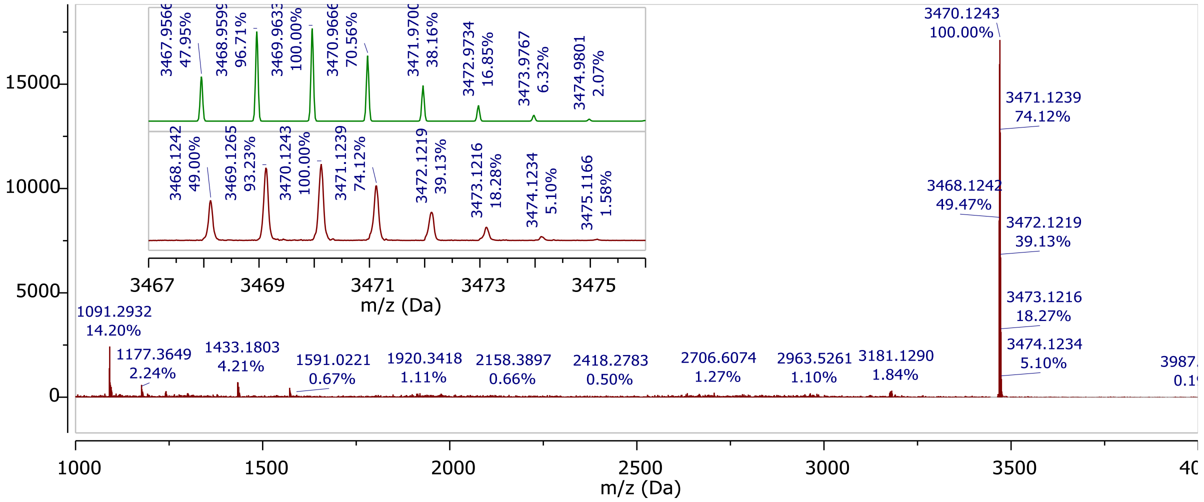

HRMS (MALDI-TOF) spectrum of **4n**.

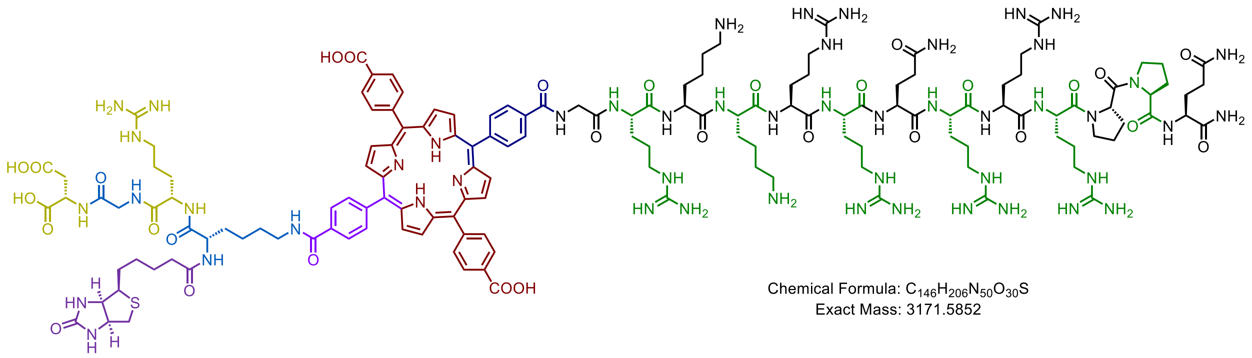

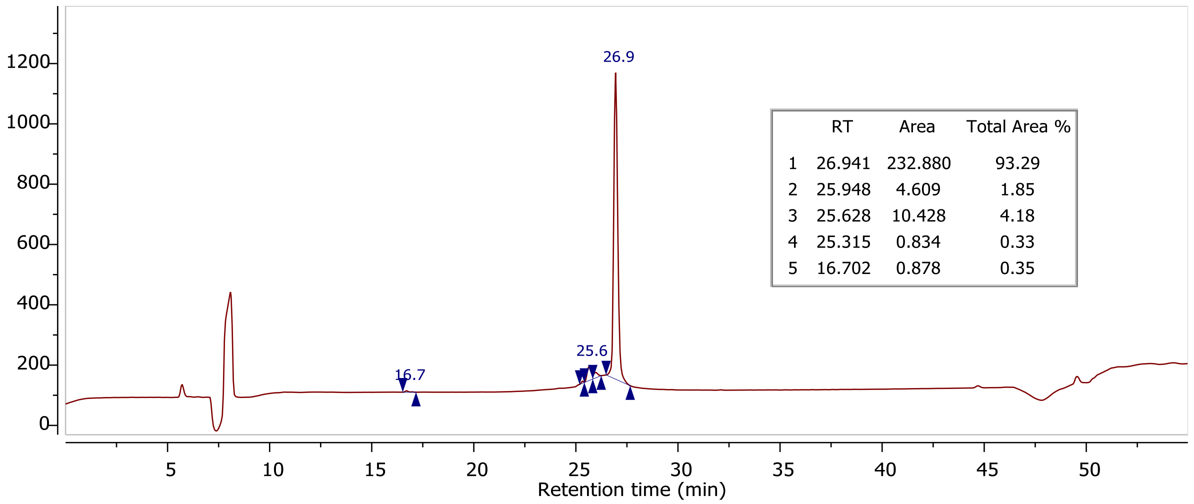

	RT	Area	Total Area %
1	26.941	232.880	93.29
2	25.948	4.609	1.85
3	25.628	10.428	4.18
4	25.315	0.834	0.33
5	16.702	0.878	0.35

HPLC chromatogram of **4o**.

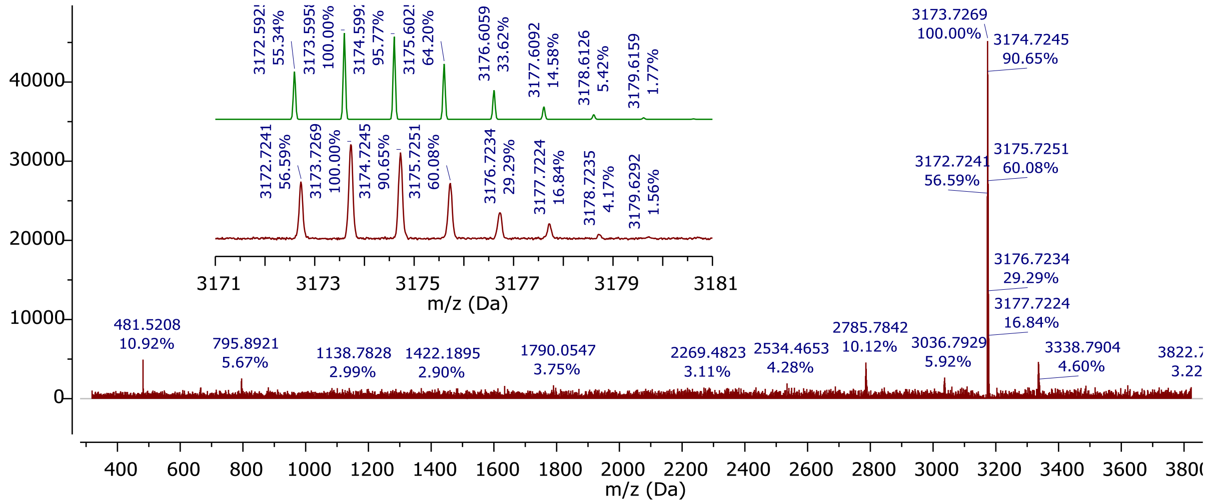

HRMS (MALDI-TOF) spectrum of **4o**.

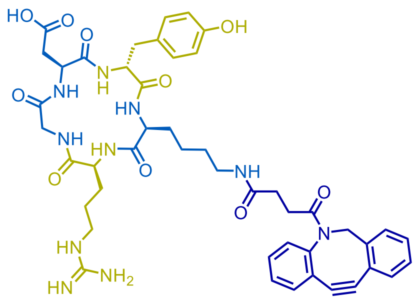

Chemical Formula: $C_{46}H_{54}N_{10}O_{10}$
Exact Mass: 906.4024

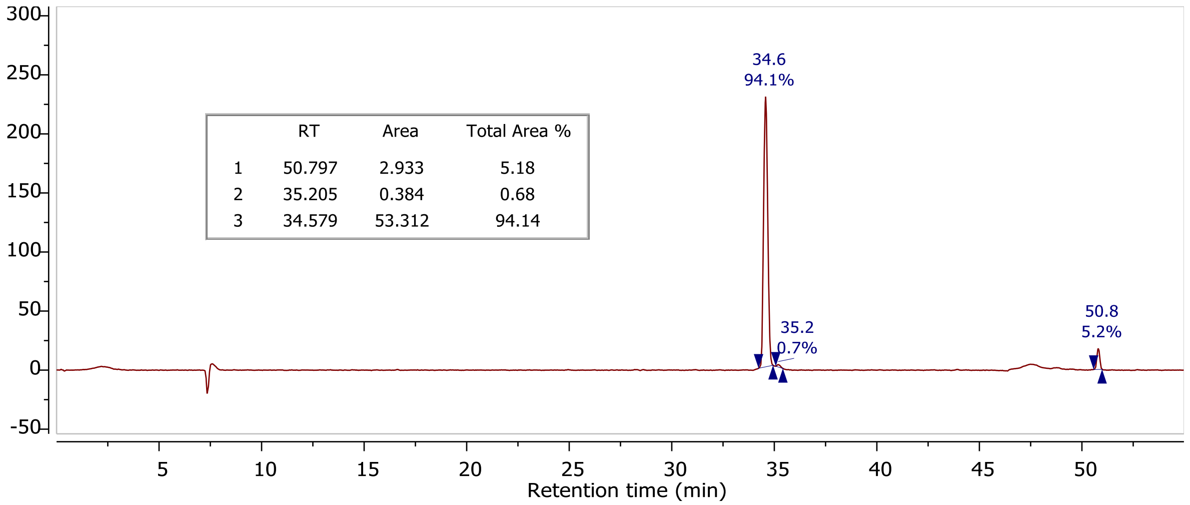

HPLC chromatogram of **DBCO-cRGD**.

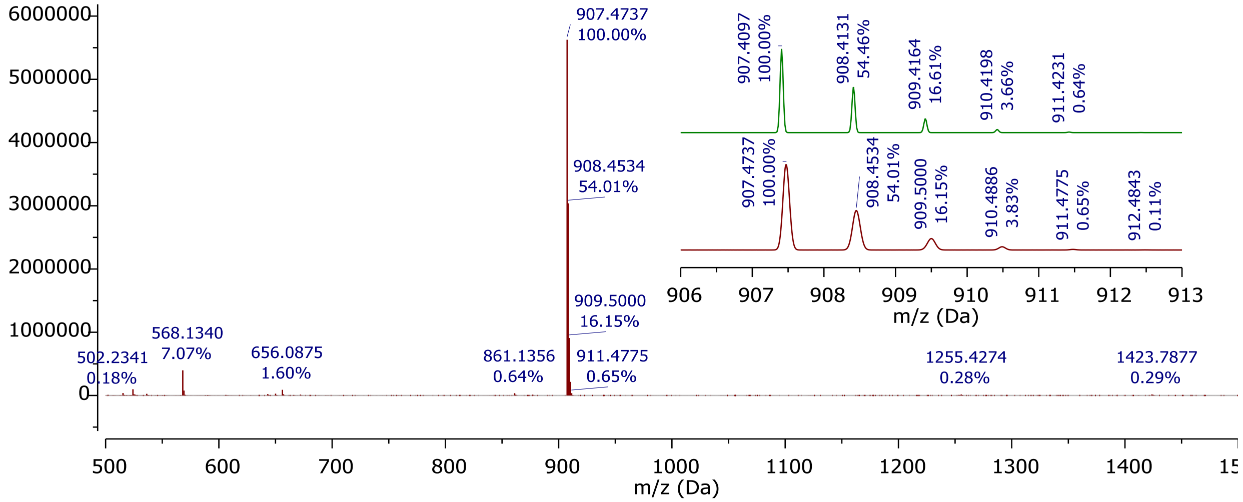

HRMS (MALDI-TOF) spectrum of **DBCO-cRGD**.
